# Trends in spatial patterns of heavy metal deposition on national park service lands along the Red Dog Mine haul road, Alaska, 2001–2006

**DOI:** 10.1371/journal.pone.0177936

**Published:** 2017-05-18

**Authors:** Peter N. Neitlich, Jay M. Ver Hoef, Shanti D. Berryman, Anaka Mines, Linda H. Geiser, Linda M. Hasselbach, Alyssa E. Shiel

**Affiliations:** 1 National Park Service, Alaska Regional Office, Anchorage, Alaska, United States of America; 2 National Oceanographic and Atmospheric Administration-National Marine Fisheries Service, Alaska Fisheries Science Center, Marine Mammal Laboratory, Seattle, Washington, United States of America; 3 United States Department of Agriculture-United States/Forest Service, Washington, District of Columbia, United States of America; 4 College of Earth, Ocean, and Atmospheric Sciences, Oregon State University, Corvallis, Oregon, United States of America; Council for Scientific and Industrial Research, INDIA

## Abstract

Spatial patterns of Zn, Pb and Cd deposition in Cape Krusenstern National Monument (CAKR), Alaska, adjacent to the Red Dog Mine haul road, were characterized in 2001 and 2006 using *Hylocomium* moss tissue as a biomonitor. Elevated concentrations of Cd, Pb, and Zn in moss tissue decreased logarithmically away from the haul road and the marine port. The metals concentrations in the two years were compared using Bayesian posterior predictions on a new sampling grid to which both data sets were fit. Posterior predictions were simulated 200 times both on a coarse grid of 2,357 points and by distance-based strata including subsets of these points. Compared to 2001, Zn and Pb concentrations in 2006 were 31 to 54% lower in the 3 sampling strata closest to the haul road (0–100, 100–2000 and 2000–4000 m). Pb decreased by 40% in the stratum 4,000–5,000 m from the haul road. Cd decreased significantly by 38% immediately adjacent to the road (0–100m), had an 89% probability of a small decrease 100–2000 m from the road, and showed moderate probabilities (56–71%) for increase at greater distances. There was no significant change over time (with probabilities all ≤ 85%) for any of the 3 elements in more distant reference areas (40–60 km). As in 2001, elemental concentrations in 2006 were higher on the north side of the road. Reductions in deposition have followed a large investment in infrastructure to control fugitive dust escapement at the mine and port sites, operational controls, and road dust mitigation. Fugitive dust escapement, while much reduced, is still resulting in elevated concentrations of Zn, Pb and Cd out to 5,000 m from the haul road. Zn and Pb levels were slightly above arctic baseline values in southern CAKR reference areas.

## Introduction

Cape Krusenstern National Monument (CAKR) is a US National Park Service (NPS) unit encompassing approximately 266,000 ha of coastal plain and alpine arctic tundra along the Chukchi Sea in northwest Alaska. Red Dog Mine, one of the world’s largest zinc (Zn) and lead (Pb) mines, is located approximately 50 km northeast of the Monument boundary and six miles from the boundary of Noatak National Preserve. The mine has operated year round since 1989 to produce Zn and Pb concentrates (approximately 50–55% concentration) in fine powder form (<40 μm, with 80% <20 μm). Concentrates are trucked 75 km from the Red Dog Mine Site (Mine Site) to the concentrate storage buildings at the Red Dog Port Site (Port Site) using eighty-ton haul trucks that travel the Red Dog Mine Haul Road (or Delong Mountain Transportation System, DMTS). The concentrates are then conveyed to barges for shipping during the short ice-free shipping season each summer (July to September).

The DMTS traverses approximately 32 km of CAKR lands. Haul trucks and other vehicular traffic had been dispersing fugitive dusts onto NPS lands for approximately 17 years at the time of the contaminant re-measurement field work in 2006 [1, 2]. These dusts were enriched with Zn, Pb, cadmium (Cd), and sulfur (S), all in reduced sulfide forms [3]. Metal-laden muds and dusts originate in the mine pit, waste rock areas, and concentrate loading and unloading facilities [3]. Once attached to vehicles, they may become dispersed onto the roadbed and surrounding tundra on the 75 km trips to and from the mine and port sites [4]. For example, mud sampled in 2006 from the wheel wells of a personnel transport vehicle contained between 18,000–23,000 mg/kg Zn and 10,000 mg/kg Pb. NPS’s pickup truck (stored and occasionally used at the mine) bore mud containing 13,000–16,000 mg/kg Zn and 3,000–4,000 mg/kg Pb. Samples collected in 2006 from the roadbed surface contained approximately 500–600 mg/kg Zn and 100 mg/kg Pb [4].

From 1991–2000, Pb and Zn concentrates in haul trucks were covered only with tarps. In 2001, the mine deployed a new fleet of trucks with hydraulically sealed lids, and in 2003 over $20 million of structural modifications were incorporated at the mine and port facilities to reduce concentrate dust losses during unloading and loading operations [5]. Gentle truck rinsing was implemented for the four months of the year with above-freezing temperatures, which helped reduce fugitive dust dispersal during those months. Traffic controls were instituted to segregate rinsed vehicles from those bearing higher surface loads of mine site muds. In addition, after 2001, the mine began to use calcium chloride as a road dust control agent to reduce dust dispersion.

Beginning in 1999, researchers began to use the feather moss *Hylocomium splendens* (Hedw.) Schimp. as a passive sampler to document patterns of airborne heavy metal deposition on NPS lands from Red Dog mining operations. In 2001, Ford and Hasselbach [2] reported elevated concentrations of Cd (>10 mg/kg) and Pb (> 400 mg/kg) in *H*. *splendens* along the haul road corridor from samples collected in 2000. Based on 2001 samples, Hasselbach et al. [1] documented strong gradients in Zn, Pb, and Cd deposition in CAKR related to the DMTS, the Port Site and the Mine Site. Heavy metal levels in moss were highest immediately adjacent to the haul road (Zn ≤ 3207 mg/kg, Pb ≤ 912 mg/kg, Cd ≤ 24.3 mg/kg). Between 0–100 m from the road, average modeled 2001 Zn, Pb and Cd concentrations in moss were 8.5 mg/kg, 366 mg/kg, and 1230 mg/kg respectively.

Analysis of subsurface soil suggested that patterns of heavy metal deposition portrayed by moss concentrations were attributable to airborne fugitive dusts and not to the subsurface soils in the study area [1]. Further, Pb concentrations in moss throughout the northern half of the study area (on both sides of the haul road) were higher than background concentrations previously reported from other Arctic Alaskan sites [6]. Collectively, these findings indicated mine-related heavy metal deposition throughout the northern portion of CAKR.

A full understanding of the fugitive dust issue also requires consideration of legal, operational and economic contexts. In 1984, Congress granted the Northwest Alaska Native Association (NANA) a 99 year easement through CAKR by Public Law 99–96 USC 43.1629. In this law and its Exhibit B (which details easement stipulations) were four clauses relevant to the fugitive dust issue. First, the US Government would retain ownership of easement lands, but the land would be subject to the easement terms “as if the lands had been conveyed to NANA”. This gave NANA and its proxy, the Alaska Industrial Development and Export Authority (AIDEA), full rights to use the lands as desired within the terms of the easement. Second, the easement required NANA to protect fish and wildlife and their habitat during mining operations. Third, the easement required NANA to “use all reasonable means” to protect both air and water quality and to comply with Alaska Department of Environmental Conservation (ADEC) standards for these resources. Fourth, the easement owner was required to take dust control measures, in consultation with NPS, to protect air quality as required by ADEC. On the federal side, the enabling legislation of CAKR [7] established this NPS unit in part “to protect habitat for and populations of, birds, and other wildlife, and fish resources; and to protect the viability of subsistence resources.” NPS’s Organic Act [8] and subsequent NPS Management Policies [9] requires of all NPS units that “the National Park Service will preserve and protect the natural resources, processes, systems, and values of units of the national park system in an unimpaired condition to perpetuate their inherent integrity and to provide present and future generations with the opportunity to enjoy them.”

The mine is the economic engine for northwest Alaska and the region’s largest employer. Since mining began in 1989, NANA has received $1.3 billion in net proceeds from the mine and has distributed approximately $820 million to other regional Native corporations under the regional profit-sharing provisions of the Alaska Native Claims Settlement Act [10]. NANA has distributed approximately $221 million in dividends to shareholders from royalties. These profits are extremely important to local, largely subsistence-based communities and have helped offset the high prices in the region including heating fuel and gasoline in excess of $12.00/gallon ($3.12/L) in small, remote villages.

NPS, NANA and the mine operator, Teck, Inc., have cooperated extensively over the past 17 years on fugitive dust mitigation, as well as research on the effects of Zn, Pb and Cd deposition on the NPS lands. The mine operator has solicited a large amount of public input on the fugitive dust issue, has commissioned a significant amount of research on biological effects of the dusts (e.g., Exponent 2007’s Ecological Risk Assessment [3]), has studied the safety of subsistence foods extensively, and has an ongoing interest in mitigation and public-private partnerships. Because of the industrial uses envisioned by the public law and easement conditions, the easement—while remaining in long-term federal ownership—has been managed according to ADEC industrial standards.

In 2006, NPS conducted an intensive study of the effects of contaminants on sensitive lichen communities and other vegetation along the DMTS in CAKR. Contaminant levels were determined in *H*. *splendens* tissue at each of 107 surveyed sites. Using Bayesian posterior predictions (e.g., [11]), consisting of the posterior predictive distribution (e.g., [12]) and a prediction grid to which both the 2001 and 2006 data sets were fitted, we compared the spatial patterns of heavy metal distribution in 2001 to that in 2006. Our primary questions were:

1. How did spatial patterns and elemental concentrations of Zn, Pb, and Cd in moss tissue change from 2001 to 2006?
2. How did these temporal changes in heavy metal concentrations vary by distance class and side of the road?

## Methods

### Study area

**Fig 1:**
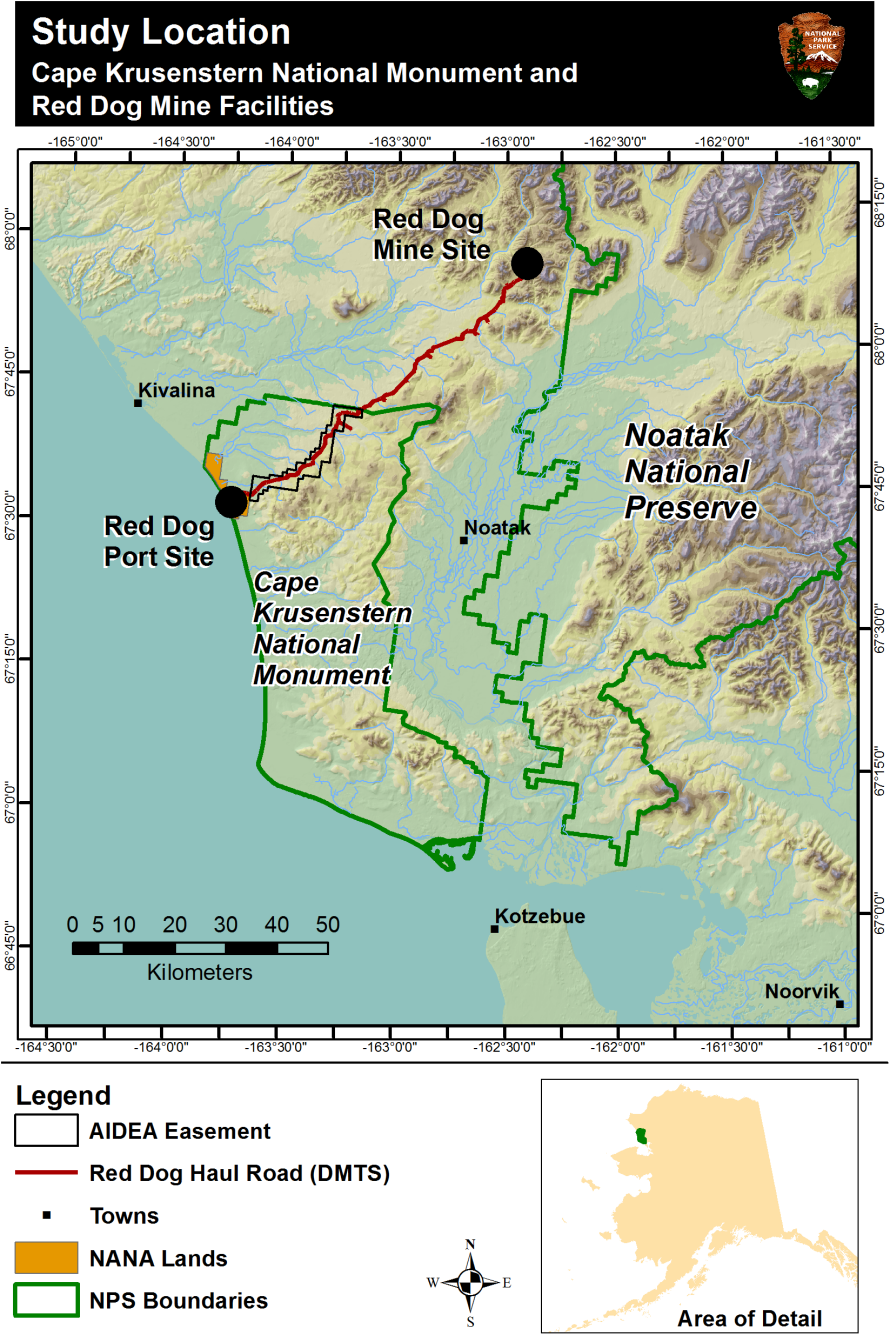
Study area for the 2001 and 2006 moss samplings. Cape Krusenstern National Monument, Red Dog Haul Road (DMTS), Red Dog Mine and Port Site, and surrounding area. Geographic coordinates are N for latitude and W for negative longitude.

CAKR is located along the Chukchi Sea, just north of Kotzebue, Alaska (Fig 1). The study area within the monument is located between 67–67.75°N latitude and 163–164.75°W longitude. The Monument is located on a coastal plain tundra ecosystem dominated by an open, low, mixed shrub-cottongrass (*Eriophorum* L.) tussock tundra [13] interspersed with well-drained hills supporting a variety of alpine lichens, forbs, and dwarf shrub species.

The climate in the study area is classified as cold-continental [14]. The mean annual temperature ranges from –7°C along the DMTS to –6°C in southern CAKR [15]. Summer temperatures in the region typically fluctuate between 2° and 18°C, while temperatures along the Chukchi Sea coast range from 4° to 13°C [3]. Winter temperatures typically range from −26° to −15°C [3]. The 1961–1990 mean January temperatures in the two closest villages of Kivalina and Noatak were –20° and –23°C, while the mean July temperatures were 11° and 14°C, respectively [15]. The modeled mean annual precipitation along the DMTS in CAKR ranges from 320 to 380 mm, with montane areas receiving up to 510 mm [16]. More than one-half the annual precipitation occurs as rain from July through September; August is the wettest month [3]. Snowfall has been recorded in every month of the year, but persistent snow cover generally lasts from mid-October to mid-May. The Chukchi Sea is covered in ice from mid-November or December through May or June [3].

Bedrock is predominantly calcareous and of Paleozoic age. Soils are poorly developed due to the cold climate, low precipitation, and the presence of continuous permafrost. Lowland soils of the monument are typically of the order Gelisol, suborder Turbel (with ice wedge terrain), and most frequently of the group Aquiturbels [13]. The DMTS haul road bisects the northern portion of the Monument. Immediately south of the haul road, the Tahinichok Mountains rise from the flat coastal plain to a maximum elevation of 502 m. South of this mountain range, Monument lands are predominantly wet rolling hills and coastal plain. The area north of the haul road is generally low-lying and gently sloping, draining into the Wulik River approximately 10 km north of the Monument boundary.

The CAKR coast typically receives high winds though wind patterns are influenced by local topography [3]. Mean monthly winds at Kotzebue, about 100 km south of the port site, average above 10 knots from September through April and blow from the east [17]. Mean wind speeds are comparable during the summer months (with an average of 10.5 knots) but are from the west. August and September are the windiest months, while the most extreme winds are associated with winter storms. Winds at the Port Site are typically from the northeast in winter months. In summer, Port Site winds are highly variable and strong winds from the south-southwest are common [3]. At the mine site, winds are predominantly from the northeast and southeast in winter months and highly variable in summer months [3]. Because of the NE-SW orientation of the road (Fig 1), prevailing easterly winds tend to drive dust to the north side of the road, which has made the north side a zone of higher contaminant accumulation than the south side in past studies [1, 2, 3].

### Study design

Re-measurement of Zn, Pb and Cd concentrations in moss tissue was nested within a larger study examining the effects of contaminants on vegetation in CAKR. Hasselbach et al. [1] found that deposition of these metals decreased logarithmically as a function of distance from the DMTS road. To address the question of vegetation community change as a function of contaminant concentrations (in a separate component of this study), we established permanent plots using a stratified-random design based on the two dominant land cover classes and several distance-from-road classes. Our overarching goals were to choose a sampling design that would reflect the exponential pattern of heavy metal decrease with increasing distance from the road while controlling for natural differences in vegetation community types and elevation. The most common vegetation types along the DMTS were Upland Moist Dwarf Birch-Ericaceous Shrub and Upland Moist Dwarf Birch-Tussock Shrub [13]. Together, these closely related classes accounted for 66% of the area along the DMTS road corridor out to a distance of 4000 m. Both of these community types are fairly rich in the lichens and bryophytes sensitive to Zn, S, and alumino-silicate road dust rich in crustal elements. To avoid confounding the pollution signal with the natural variation in vegetation due to elevation, we avoided sampling in any alpine communities and restricted our sampling to between 60 and 250 m in elevation ($\bar{x}$ = 130 m).

Sampling distances from the road were chosen to obtain an even spread of values along the heavy metals deposition gradient models reported by Hasselbach et al. [1]. Twelve non-linear “transects” were created according to the distances in Table 1, with points at 10, 50, 100, 300, 1000, 2000, and 4000 m. To help model fine-scale spatial autocorrelation, one additional plot (termed an “autocorrelation plot”) was established in each transect at a random distance 10 to 20 m from the 1,000, 2,000, or 4,000 m plot in each transect. Each “transect” consisted of a group of points within a land cover class at specified distance classes. Points were chosen at random in ArcGIS 9.2 [18] in close geographic proximity to a line, but did not represent a strictly linear feature, per se. Random selections were determined using a random number generator assigned to up to 10 pixel groups of interest closest to the transect line at a particular distance. Linear transects were tested in the planning stages, but rejected as it was not possible to find both the correct land cover classes and distances along a straight line.

**Table 1:** Number of plots by distance from the DMTS haul road in 2006 sampling.

Distance from Road (m)	Plots (N)	Plot Type	Tissue Samples (N)
3	4	Moss Tissue Only	4
10	12	Standard	17
50	12	Standard	11
100	12	Standard	13
300	12	Standard	14
1,000	12	Standard	14
1,000	8	Autocorrelation	8
2,000	11	Standard	13
2,000	1	Autocorrelation	1
4,000	11	Standard	15
4,000	3	Autocorrelation	3
42,000–48,000	4	Reference 1	5
60,000–64,200	6	Reference 2	7

Standard and autocorrelation plots sampled both vegetation and moss tissue. A tissue sample, and in some cases a field duplicate sample, was collected at each plot (with the exception of one plot at 50m in which no tissue was available). The four plots closest to the road sampled moss tissue only. Autocorrelation plots were established to provide autocorrelation estimation in the posterior predictions model. All of the 125 samples from 104 plots and 4 additional tissue sample locations in 2006 were used in the current model.

Two groups of reference plots were chosen at random from points in CAKR with sufficient representation of the two desired land cover classes, and at a distance of at least 40 km from the DMTS. Each reference area had either 2 or 3 replicates of each land cover class, with a total of 10 plots (Table 1).

**Fig 2:**
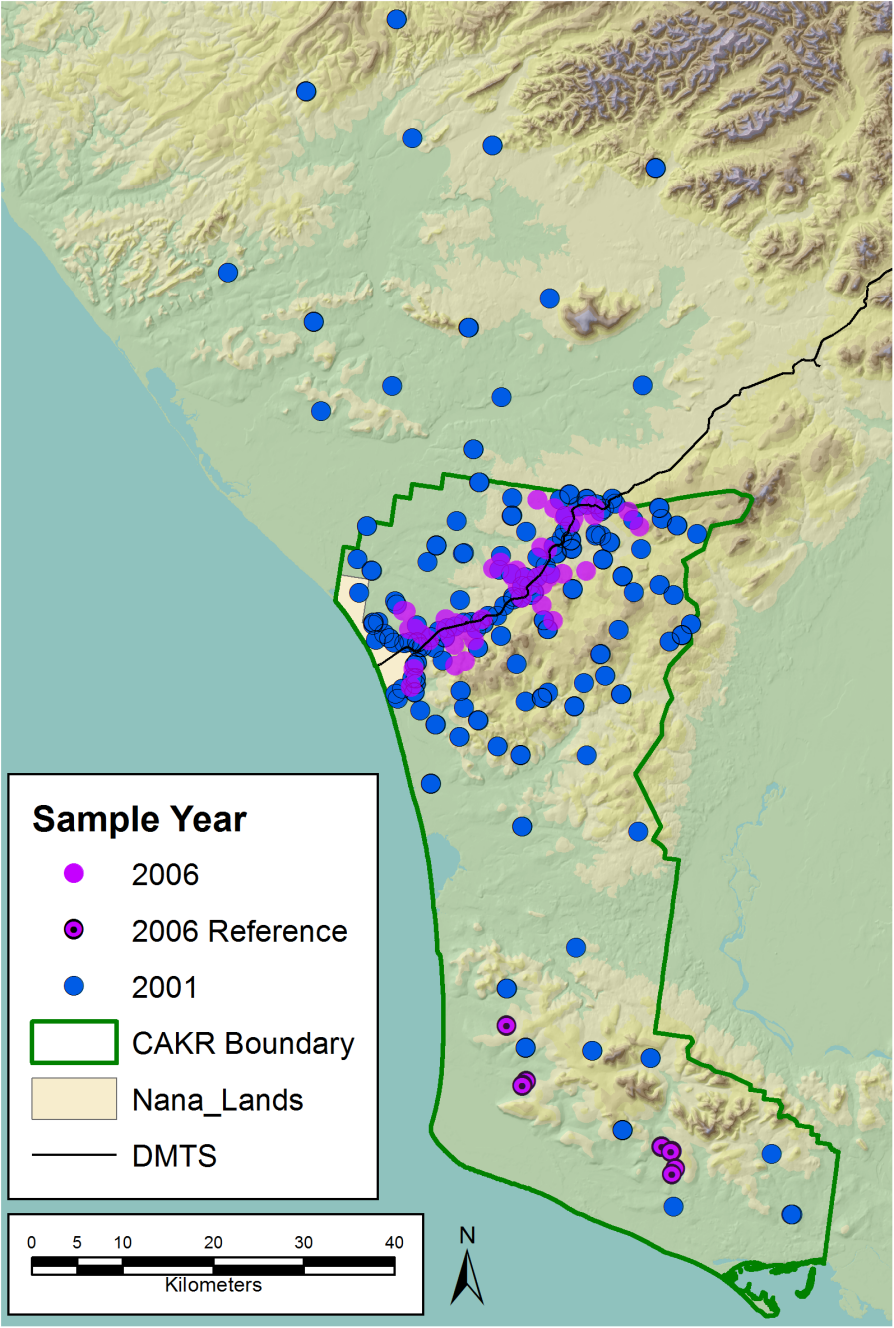
Moss tissue sample points, 2001 and 2006. Sample points for *Hylocomium splendens* in the vicinity of CAKR and the DMTS haul road from 2001 [1] and 2006 studies. The 2006 points in southern CAKR were established as reference sites.

The 2001 sampling methods are reported in Hasselbach et al. [1] and summarized in Table 2. Note that the statistical design strata are defined differently in the two studies: in addition to road-based distances, Hasselbach et al. [1] uses Port Site -based distances (strata 4, 5). Sampling points from both projects are shown in Fig 2. The 2001 and 2006 sampling efforts each occurred during the month of July.

**Table 2:** Stratified sampling design from 2001 moss tissue sampling [1].

Stratum	Distance from Road or Port	Sample Locations (N)
1	0–10 m from road	12
2	10–250 m from road	13
3	250–1,000 m from road	18
4	0–100 m from NANA^a^ Port Site boundary	8
5	100–1,000 m from NANA^a^ Port Site boundary	8
6	1,000–16,000 m from road	66
7	Southern portion of Monument (> 16,000 m south of road)	12
8	North of Monument boundary (> 16,000 m north of road)	14

All 244 samples from 151 sites (classed by N) sampled in 2001 were used in the current model. Sampling strata represent geographic locations from which moss tissue was obtained. Strata are defined here in a statistical sense as components of a stratified study design, rather than as components of a soil profile.

^a^ NANA Regional Corporation Lands. Note that Strata 4 and 5 used Port Site-based distances rather than road-based distances.

### Field methods

After sample plot locations were selected in GIS, precise coordinates (error ≤ 1 cm horizontal) were uploaded into Trimble GeoExplorer XH and GeoExplorer XT GPS’s [19]. Researchers located each point on the ground with an estimated real-time horizontal position error ranging from 10 to 200 cm and permanently marked and recorded each location in GPS. GPS plot data were later post-processed for sub-meter accuracy (10–50 cm in most cases). Locations for plots 10 and 50 m from the DMTS were adjusted for greater precision using a measuring tape from the roadbed’s toe slope contact point with the tundra.

Within 10 m of each plot (but at the same distance to the DMTS), approximately 2 L (fairly compacted and yielding 40–80 g dry weight) of the moss *Hylocomium splendens* was collected. Field duplicates were collected from 10% of the sample sites and during the moss tissue cleaning process, an additional 10% of samples were subdivided into laboratory duplicates for laboratory quality assurance.

### Laboratory analysis

The 2001 moss sample analysis is summarized in Hasselbach et al. [1]. The most recent 3–5 years of the 2006 moss tissue were collected by snipping the moss at the corresponding node demarcating annual growth segments. This tissue was then ground, homogenized and analyzed at the same laboratory (University of Minnesota Research Analytical Laboratory, St. Paul, MN) using nearly identical protocols. Moss tissue was analyzed for total sulfur, total nitrogen and a suite of 15 metals. Detailed laboratory analysis methods are provided in S1 File.

### Bayesian spatial regression and prediction

Spatial regression models, using Bayesian methods [12], were fit to Zn, Pb, and Cd concentration in *H*. *splendens* tissue from the two independent sampling efforts in 2001 [1] and 2006. After model fitting, posterior predictive distributions [11] were used to simulate predictions from each model on a common spatial grid, which allowed spatial comparison between the two years to assess changes for each element.

The Bayesian regression modeling followed Hasselbach et al. [1] (2005). The measured concentration of Zn, Pb and Cd were transformed to natural logarithm values. Let *Y*_*i*_ denote the transformed value for the *i*th location, then the regression model was

$$Y_{i}=\beta_{0}+\beta_{1}d_{1}+\beta_{2}s_{1}+\beta_{3}d_{i}:s_{i}+U_{i}+Z_{i}+\epsilon_{i,}$$ (1)

where *d*_*i*_ is log (distance from road), *s*_*i*_ is a binary indicator of north or south of the road, *d*_*i*_: *s*_*i*_ is their interaction, *β*_*0*_, *β*_*1*_, *β*_*2*,_ and *β*_*3*_ are regression parameters, *U*_*i*_ is a random effect for site (to account for duplicate samples per site) with variance *θ*_*1*_, *Z*_*i*_ is a spatially autocorrelated random effect, and ϵ_*i*_ is uncorrelated (independent) error, which includes the replicate laboratory sampling, with variance *θ*_0_. The spatial autocorrelation for *Z*_*i*_ among locations was modeled with the exponential autocovariance model,

$$C(h)=\theta_{2}exp(-h/\theta_{3}),$$ (2)

where *h* is the distance (m) between any two locations. There are four covariance parameters: the nugget *θ*_0_, the variance of the random effect for location *θ*_1_, the partial sill *θ*_2_, and the range *θ*_3_. We used flat priors on *β*_*0*_, *β*_*1*_, *β*_*2*,_ and *β*_*3*_ and the reference prior [20] on *θ*_0_, *θ*_1_, *θ*_2_, and *θ*_3_. The posterior distribution of all parameters was sampled using Markov chain Monte Carlo methods (MCMC, [21]). We used a “burn-in” of 4,000 iterations and then retained each 10th iteration of the next 2000, leaving a sample from the posterior distribution of 200 iterations for further inference. A separate model was fit for each element, Zn, Pb, or Cd, to the data from each year, 2001 and 2006.

**Fig 3:**
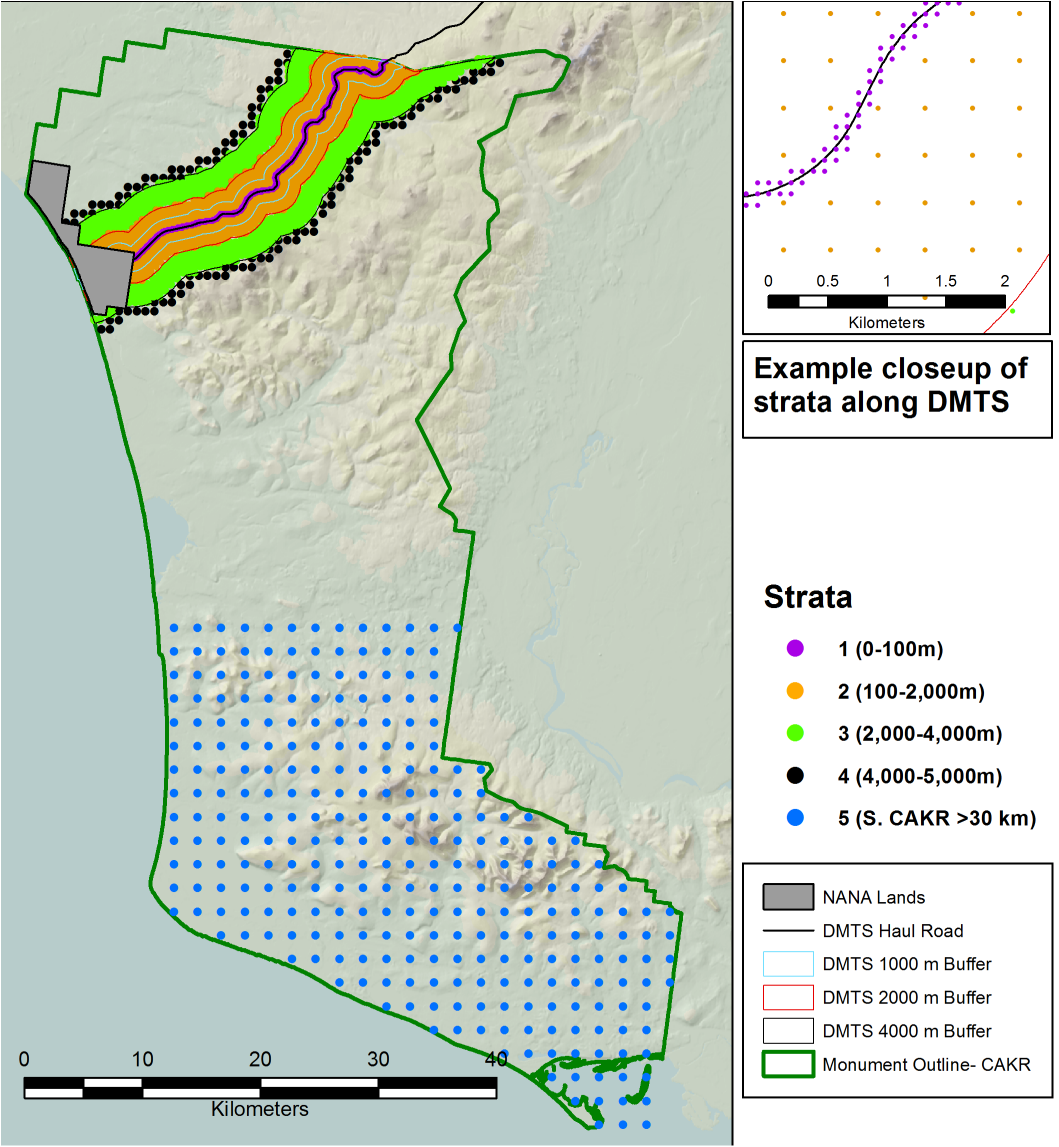
Prediction grid used to model the contaminant concentrations from sample points shown in Fig 2. Upper right panel shows an example of a close up view of the DMTS haul road with portions of strata 1, 2 and 3.

A prediction grid of 2,374 points, consisting of five strata (Table 3) was generated by defining grid points that varied in density among strata. Denser prediction grids were better able to capture more rapid changes in Zn, Pb, and Cd nearer to the haul road. We placed as many points in each stratum as was computationally feasible (Fig 3). For each of the 200 MCMC iterations, the sampled parameters and observed data were used to predict values at the prediction grid locations yielding 200 prediction surfaces for each element and each year. All prediction values were back-transformed to the original scale for further summaries of the posterior predictive distributions, which we denote as ${Ŷ}_{year,j,}^{\left[k\right]}$ for the *k*th simulated prediction at the *j*th prediction site for year equal to 2001 or 2006. The point-wise predictions, across all strata, were used for mapping. In addition, block averages were computed within each stratum, for each element, each year, and each of the 200 prediction surfaces. We computed the mean stratum concentration as

**Table 3:** Stratified sampling design for Bayesian posterior predictions prediction grid.

Stratum	Distance from Road	N
1	0–100 m from road	697
2	100–2,000 m from road	743
3	2,000–4,000 m from road	507
4	4,000–5,000 m from road	121
5	South CAKR, >30,000 m from road	289

N provides the number of prediction points in the grid representing each stratum. The model was developed with the posterior predictions grid (Fig 3) using the samples from 2006 (Table 1) and 2001 (Table 2 [1]).

$${\hat{B}}_{{year,stratum}_{t}}^{\left[k\right]}=\frac{\sum_{{j\epsilon stratum}_{t}}{Ŷ}_{year,j}^{\left[k\right]}}{N_{{stratum}_{t}}},$$ (3)

for the *k*th prediction surface for stratum *t*, where $N_{{stratum}_{t}}$ was the number of prediction grid points in the *t*th stratum (Table 3). The mean estimate for each stratum, then was the average of ${\hat{B}}_{{year,stratum}_{t}}^{\left[k\right]}$ over the *k* = 1,…, 200 simulations, the standard error was the standard deviation among the 200 values, and the 2.5^th^ and 97.5^th^ percentiles were computed from the ordered values of ${\hat{B}}_{{year,stratum}_{t}}^{\left[k\right]}$. We also computed the average change from 2001 to 2006 as the average of ${\hat{B}}_{{2006,stratum}_{t}}^{\left[k\right]}-{\hat{B}}_{{2001,stratum}_{t}}^{\left[k\right]}$ over the *k* = 1,…,200 simulations, and the percent change as the average of 100 *$({\hat{B}}_{{2006,stratum}_{t}}^{\left[k\right]}-{\hat{B}}_{{2001,stratum}_{t}}^{\left[k\right]})/{\hat{B}}_{{2001,stratum}_{t}}^{\left[k\right]}$.

Again, the standard errors were computed from the standard deviation among the 200 values, and the 2.5th and 97.5th percentiles were computed from the ordered values from the 200 simulations of the average change and percent change. The probability of decrease was computed as $\frac{1}{200}\sum_{k=1}^{200}I\;({\hat{B}}_{{2006,stratum}_{t}}^{\left[k\right]}<{\hat{B}}_{{2001,stratum}_{t}}^{\left[k\right]})$, where *I(a)* is the indicator function (equal to 1 if the expression *a* is true, otherwise it is 0. Both the prediction grid and the model output maps were created using ArcGIS software [17]. To provide a measure of the uncertainty of each point prediction (on each year and for each element), the reciprocal of the coefficient of variation (CV), computed as the estimate/standard-error-of-estimate, was divided into four quantiles which were mapped with increasing size. Thus, not only was each point prediction color-coded by concentration class, but was sized according to the CV, where the largest dots had the lowest CV (the most certainty).

## Results

### Regression of distance to road vs. concentrations

**Fig 4:**
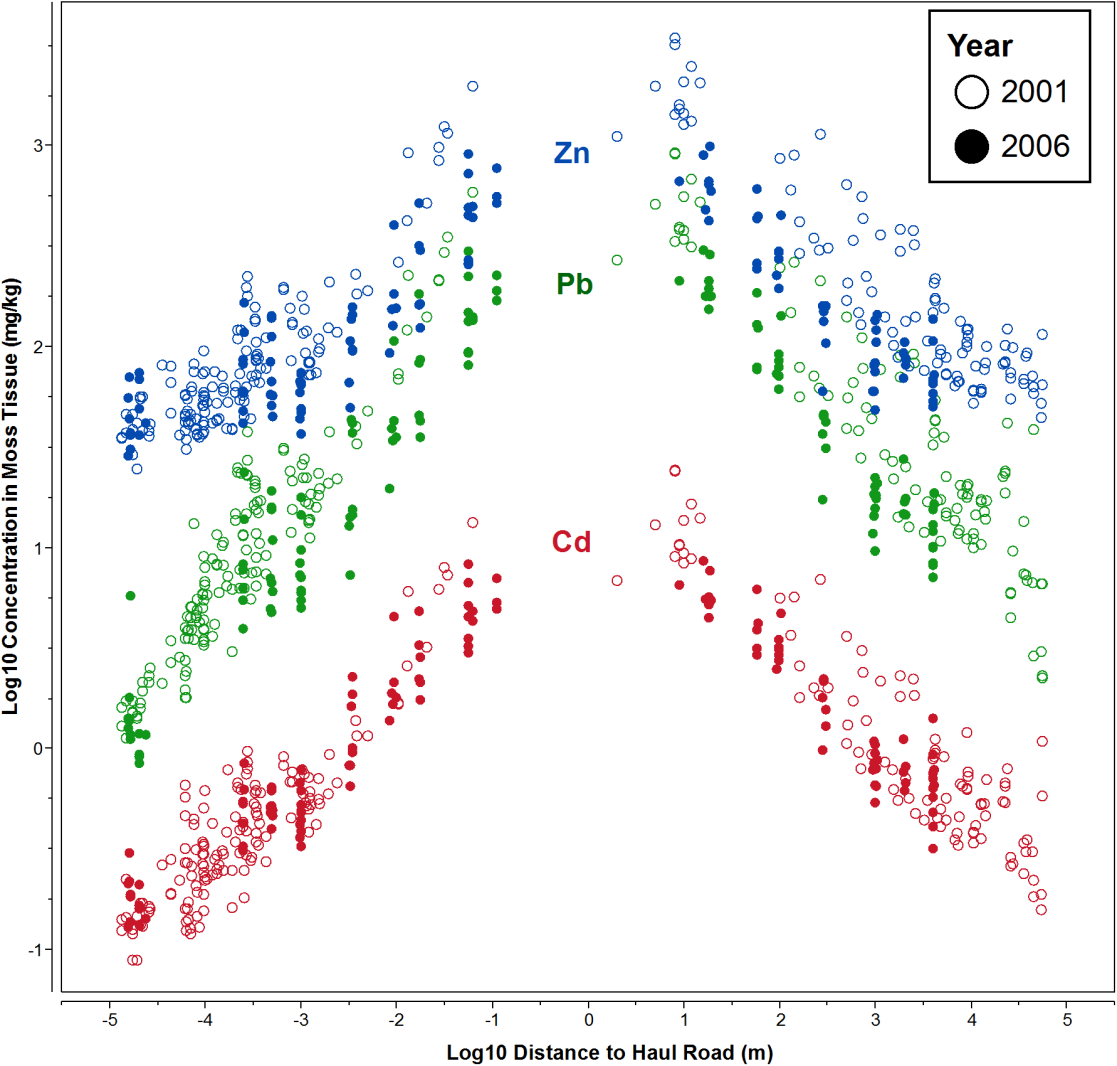
Concentrations of Zn, Pb, and Cd away from haul road, 2001 and 2006. Elemental concentrations of Zn, Pb, and Cd in *Hylocomium splendens* tissue vs. distance to the Red Dog haul road plotted on a log_10_-log_10_ scale. Each dot represents a moss sample point in either 2001 (open circles) or 2006 (solid circles). Sample points north of the road are plotted as positive distances, while those south of the road are negative. Table 4 displays the regression coefficients of this plot using natural logarithms.

Hasselbach et al. (2005) graphed the unmodeled moss tissue concentrations of Zn, Pb and Cd in field samples against the distance from the DMTS haul road. We present the same data in Fig 4, updated with the 2006 samples. A reduction of concentration is apparent for the 2006 points relative to the 2001 points, with spatial regression coefficients provided in Table 4. All elements showed a significant decrease with increasing distance from road, as seen by the credible intervals which are far from zero. Changes from 2001 to 2006 are apparent, with either a lowering of the intercept values (Cd and Zn), or a steeper slope (Pb and Zn), indicating lower concentrations of Cd, Pb, and Zn in 2006. Also, there is generally a difference due to side-of-road, either by causing a shift in the intercept or through a significant interaction with slope.

**Table 4:** Summaries of posterior distributions for regression coefficients in Eq 1.

**Cd**								
	2001				2006			
	Est.	SE	Lower	Upper	Est.	SE	Lower	Upper
*β*_*0*_	3.948	0.379	3.222	4.637	3.513	0.305	2.935	4.128
*β*_*1*_	-0.479	0.030	-0.542	-0.420	-0.485	0.031	-0.546	-0.419
*β*_*2*_	0.177	0.269	-0.324	0.759	-0.499	0.197	-0.910	-0.091
*β*_*3*_	-0.108	0.048	-0.235	-0.031	0.022	0.036	-0.057	0.086
**Pb**								
	2001				2006			
*β*_*0*_	7.505	0.683	5.930	9.006	7.778	0.471	7.020	8.905
*β*_*1*_	-0.516	0.024	-0.563	-0.470	-0.622	0.038	-0.696	-0.548
*β*_*2*_	0.619	0.269	0.046	1.127	-0.513	0.290	-1.154	0.077
*β*_*3*_	-0.196	0.042	-0.287	-0.121	-0.025	0.052	-0.129	0.070
**Zn**								
	2001				2006			
*β*_*0*_	9.406	1.523	5.375	13.613	9.193	2.738	4.197	12.939
*β*_*1*_	-0.453	0.019	-0.486	-0.414	-0.527	0.034	-0.596	-0.464
*β*_*2*_	0.271	0.253	-0.266	0.774	-0.686	0.247	-1.198	-0.220
*β*_*3*_	-0.131	0.046	-0.231	-0.047	0.031	0.052	-0.072	0.129

Est. is the mean and SE is the standard deviation of the posterior distribution. Lower and Upper are the 2.5% and 97.5% endpoints of the 95% credible intervals. Note that while these coefficients were calculated using natural logarithms, Fig 4 is displayed using log_10_ for greater interpretability.

### Spatial patterns of heavy metals distribution model

#### Pointwise mapping

**Fig 5:**
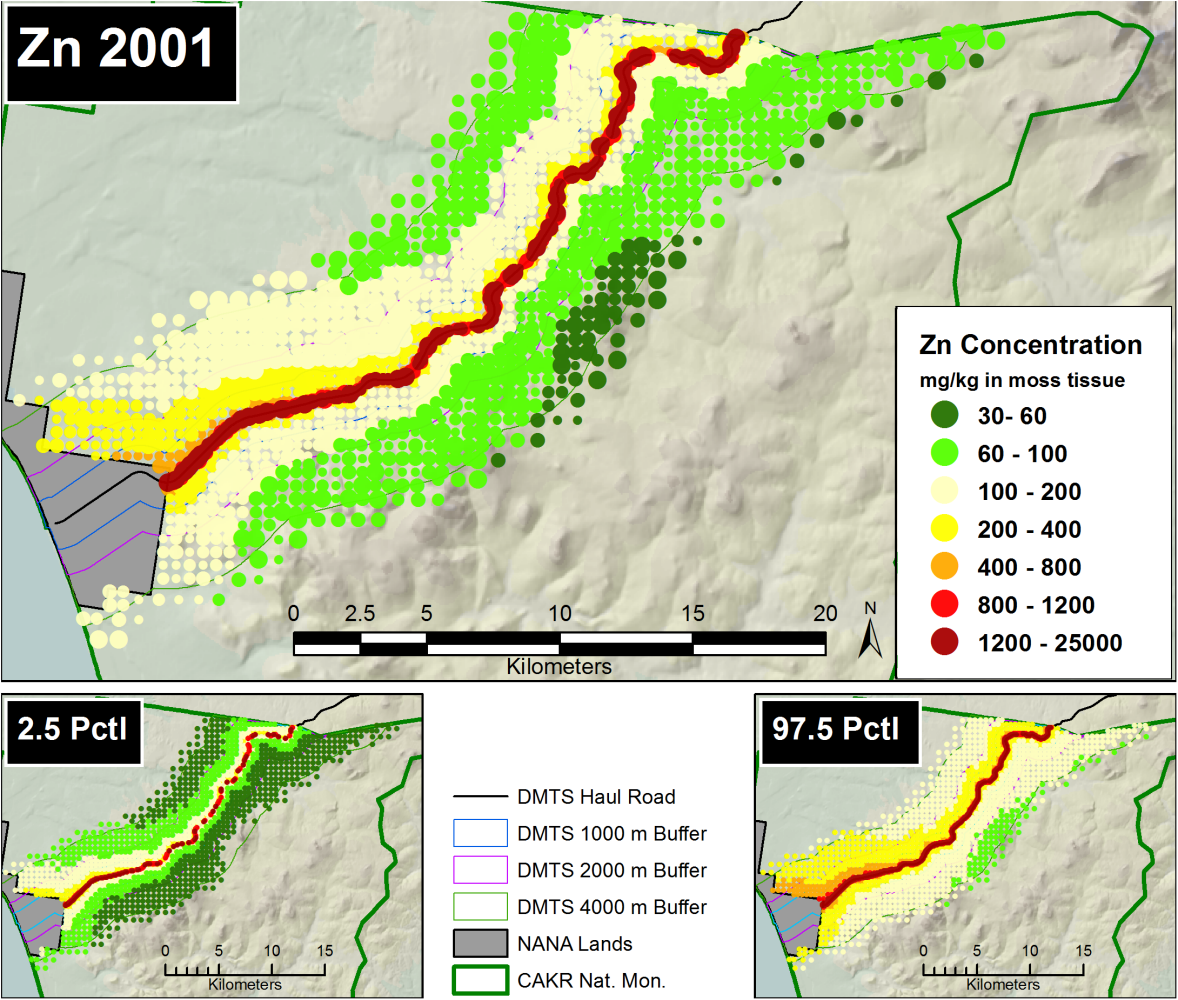
Modeled 2001 Zn moss tissue concentrations along the DMTS. The 2.5^th^ and 97.5^th^ percentiles (lower and upper bound of the 95% interval) of the modeled concentrations are shown at right. Dots on the main graph are sized proportionally in four classes by the quartile distributions of the reciprocal of the CV.

**Fig 6:**
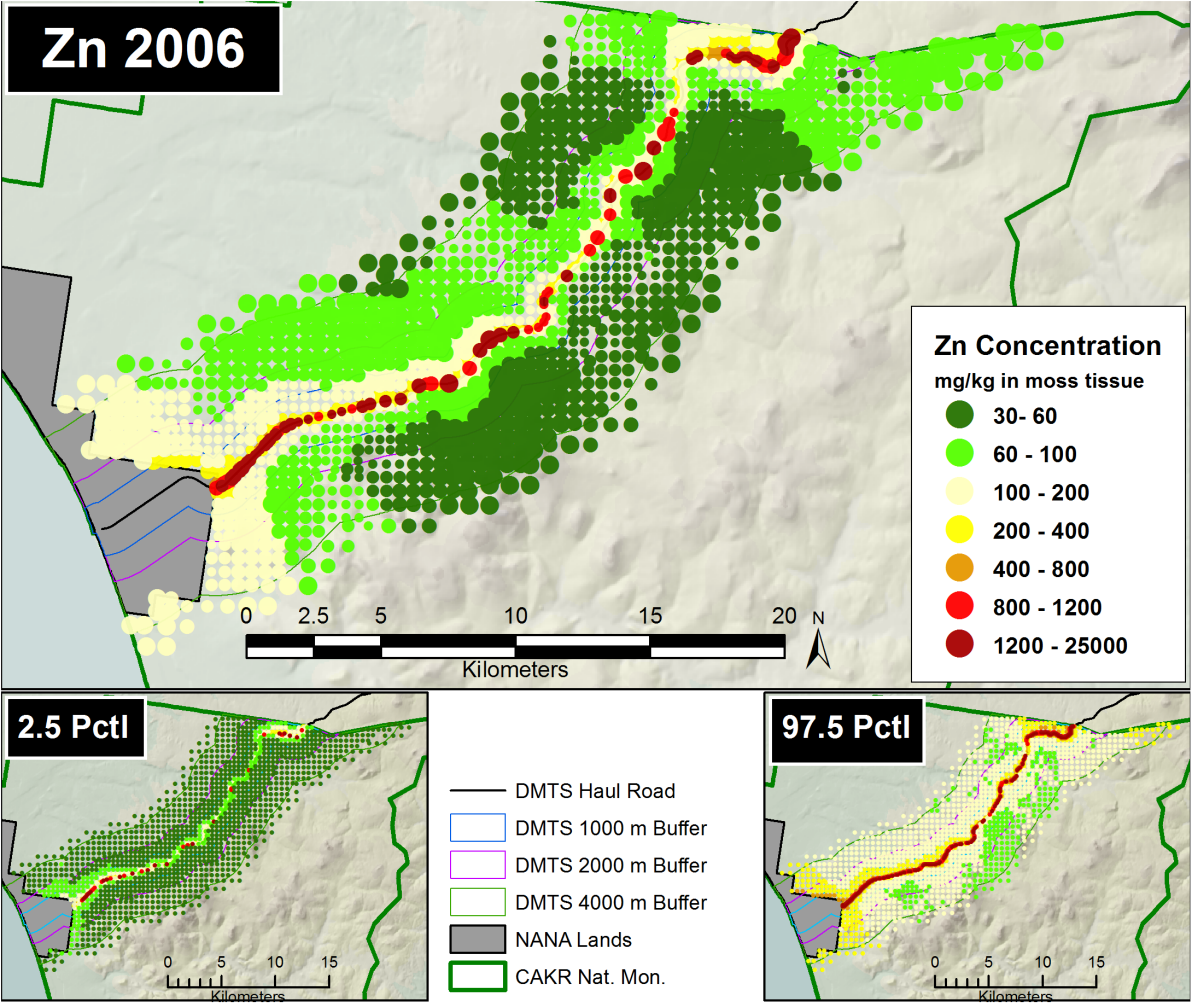
Modeled 2006 Zn moss tissue concentrations along the DMTS. The 2.5^th^ and 97.5^th^ percentiles (lower and upper bound of the 95% interval) of the modeled concentrations are shown at right. Dots on the main graph are sized proportionally in four classes by the quartile distributions of the reciprocal of the CV.

**Fig 7:**
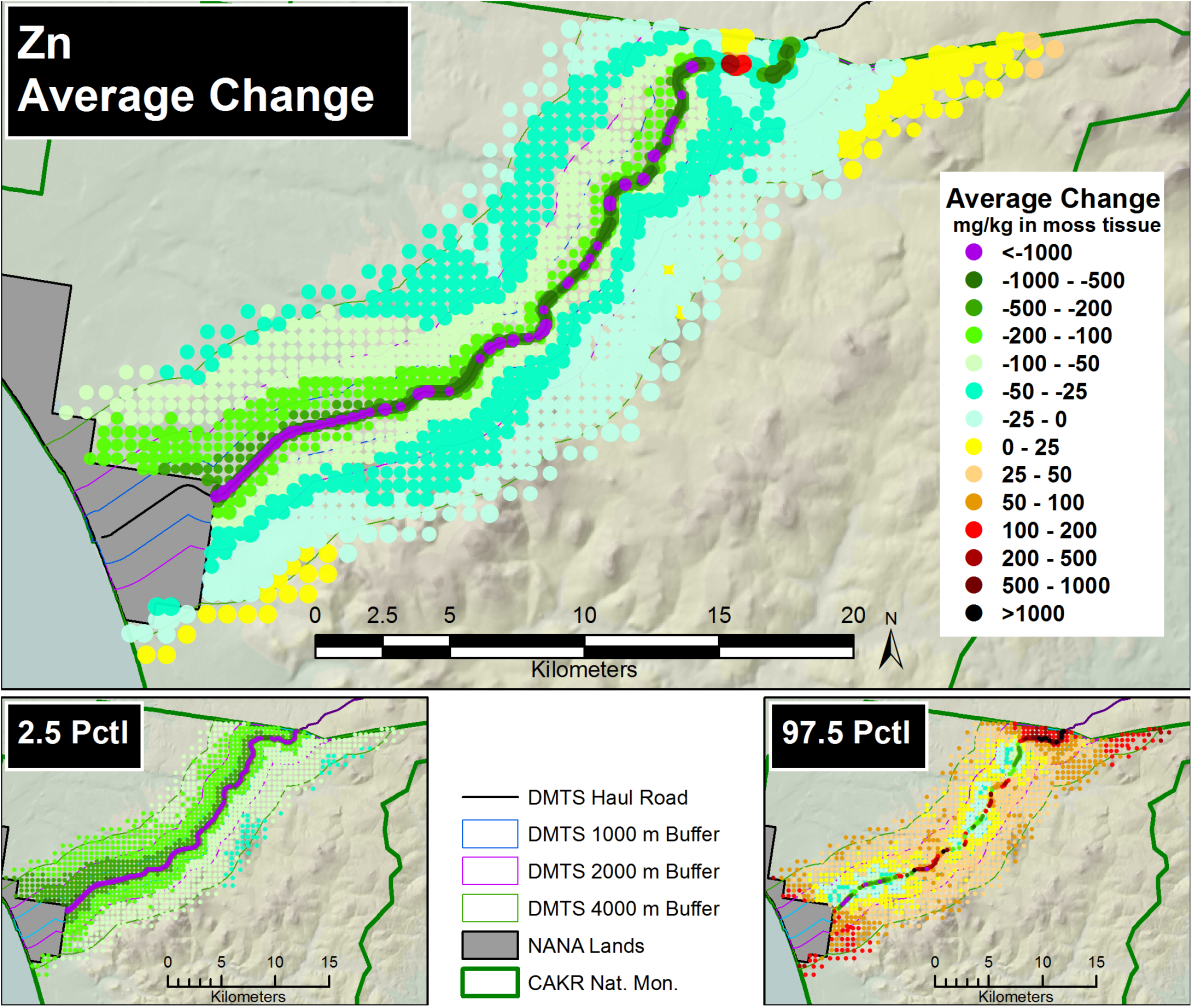
Average change in Zn concentrations, 2001–2006. Average change between modeled 2001 and 2006 Zn moss tissue concentrations along the DMTS. The 2.5^th^ and 97.5^th^ percentiles (lower and upper bound of the 95% interval) of the modeled concentrations are shown at right. Dots on the main graph are sized proportionally in four classes by the quartile distributions of the reciprocal of the CV.

**Fig 8:**
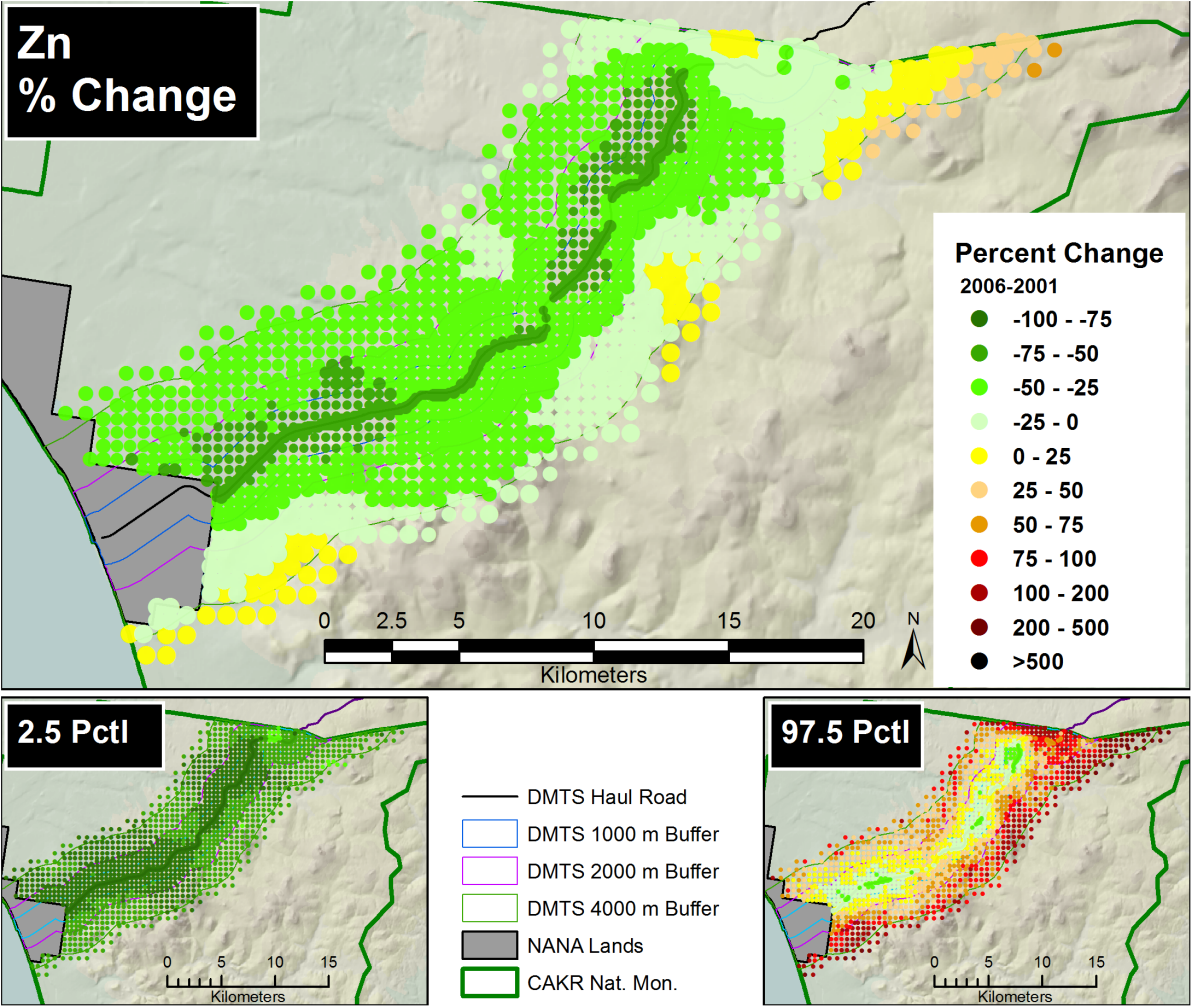
Percent change in Zn concentrations, 2001–2006. Percent change between 2001 and 2006 Zn moss tissue concentrations along the DMTS. The 2.5^th^ and 97.5^th^ percentiles (lower and upper bound of the 95% interval) of the modeled concentrations are shown at right. Dots on the main graph are sized proportionally in four classes by the quartile distributions of the reciprocal of the CV.

**Fig 9:**
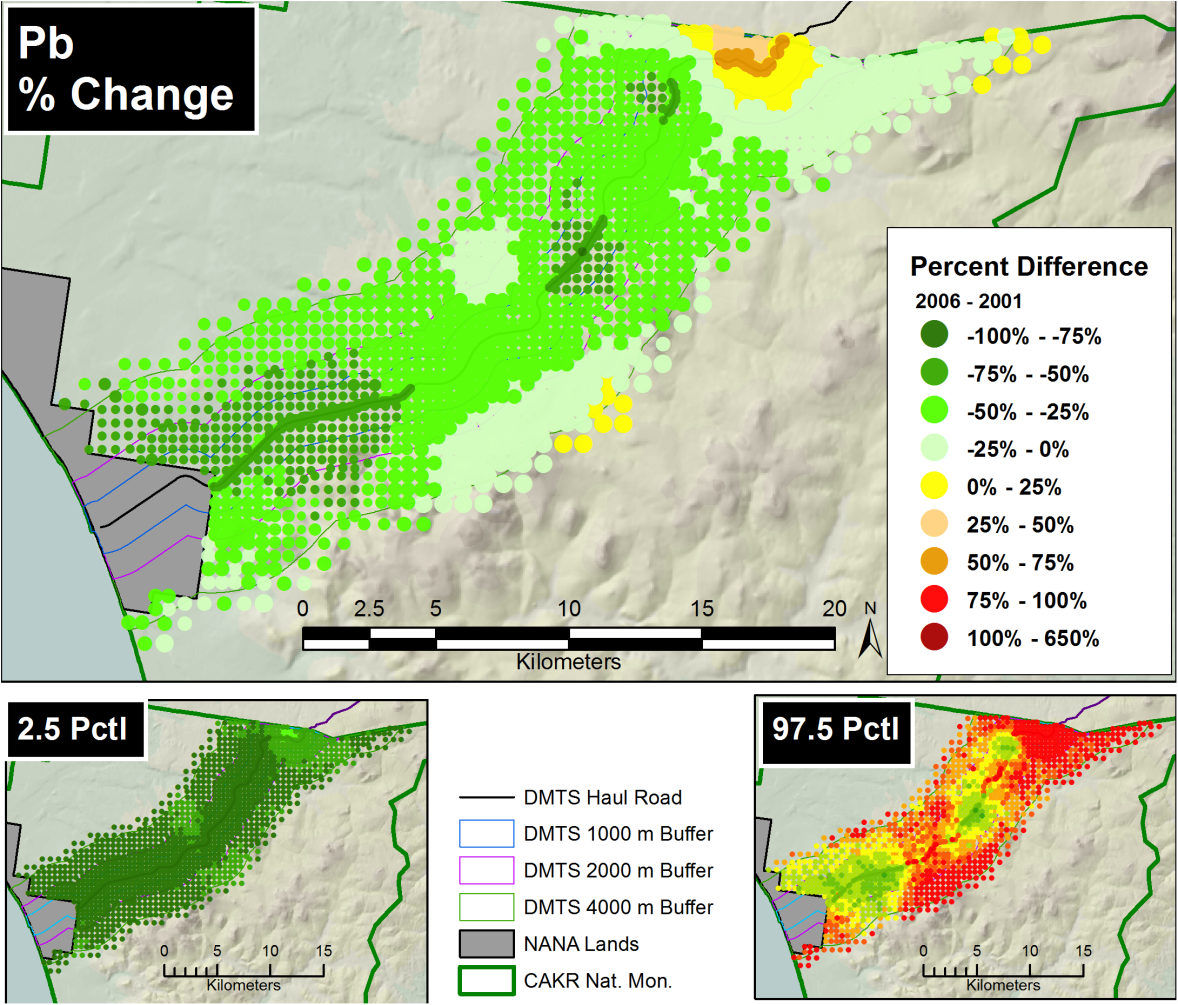
Percent change in Pb concentrations, 2001–2006. Percent change between 2001 and 2006 Pb moss tissue concentrations along the DMTS. The 2.5^th^ and 97.5^th^ percentiles (lower and upper bound of the 95% interval) of the modeled concentrations are shown at right. Dots on the main graph are sized proportionally in four classes by the quartile distributions of the reciprocal of the CV.

**Fig 10:**
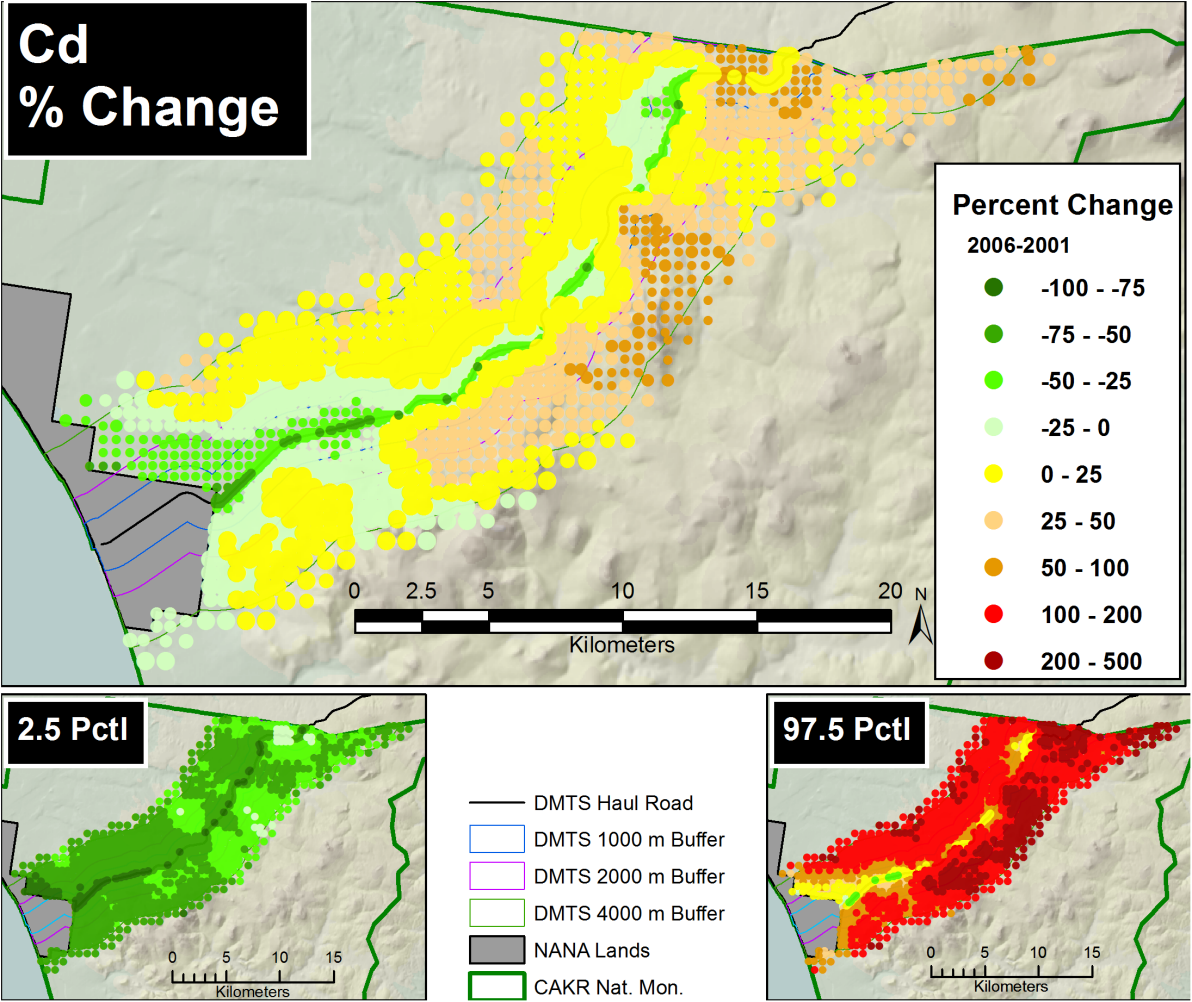
Percent change in Cd concentrations, 2001–2006. Percent change between 2001 and 2006 Cd moss tissue concentrations along the DMTS. The 2.5^th^ and 97.5^th^ percentiles (lower and upper bound of the 95% interval) of the modeled concentrations are shown at right. Dots on the main graph are sized proportionally in four classes by the quartile distributions of the reciprocal of the CV.

Prediction of Zn, Pb and Cd moss concentrations was modeled at two scales—a fine-scale, pointwise model designed for mapping and a coarse-scale, stratum-based model designed for summarization by stratum. The pointwise predictions of the 3 elemental moss concentrations for 2001 and 2006 were then mapped at two scales on the prediction grid: for the DMTS haul road corridor (Figs 5 and 6, S5, S6, S12 and S13 Figs) at 1:240:000, and for CAKR as a whole at 1:550,000 (S1 and S2 Figs, S8, S9, S15 and S16 Figs).The average difference in Zn, Pb and Cd predicted values between years (Fig 7, S3, S7, S10, S14 and S17 Figs) and percent change between years (Figs 8–10, S4, S11 and S18 Figs) were mapped at these same scales. At a coarse grid scale, all three elements showed sizeable decreases in mean values on the order of 20–75% immediately adjacent to the DMTS (Stratum 1, 0–100 m). Zn and Pb showed larger decreases in this stratum (typically 40–75%), while Cd showed moderate decreases (20–75% with the greatest decreases close to the Port Site).

**Fig 11:**
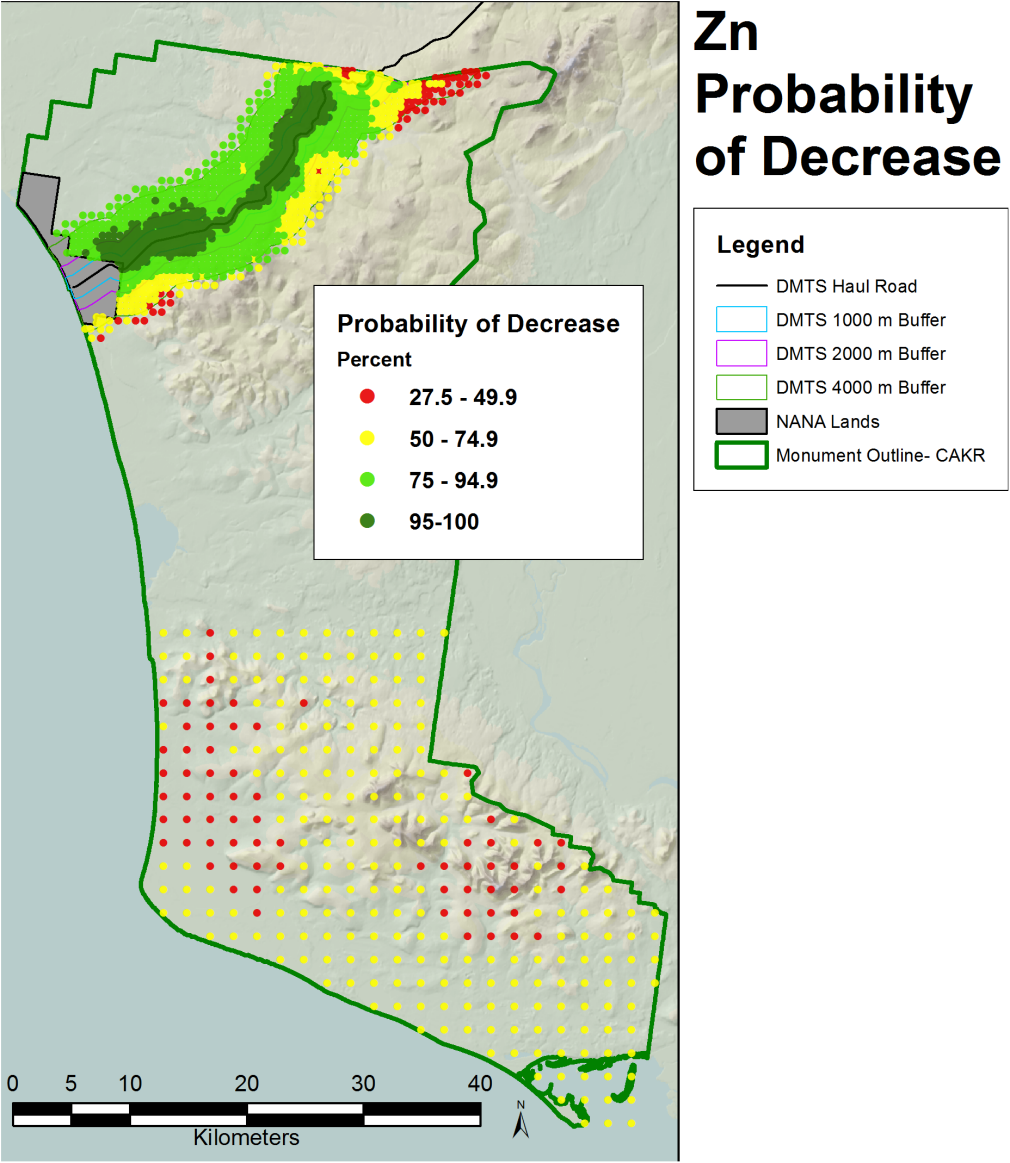
Probability of Zn decrease, 2001–2006. Probability of decrease between 2001 and 2006 Zn moss tissue concentrations based on the percentage of negative values of change among the 200 iterations at each prediction point.

**Fig 12:**
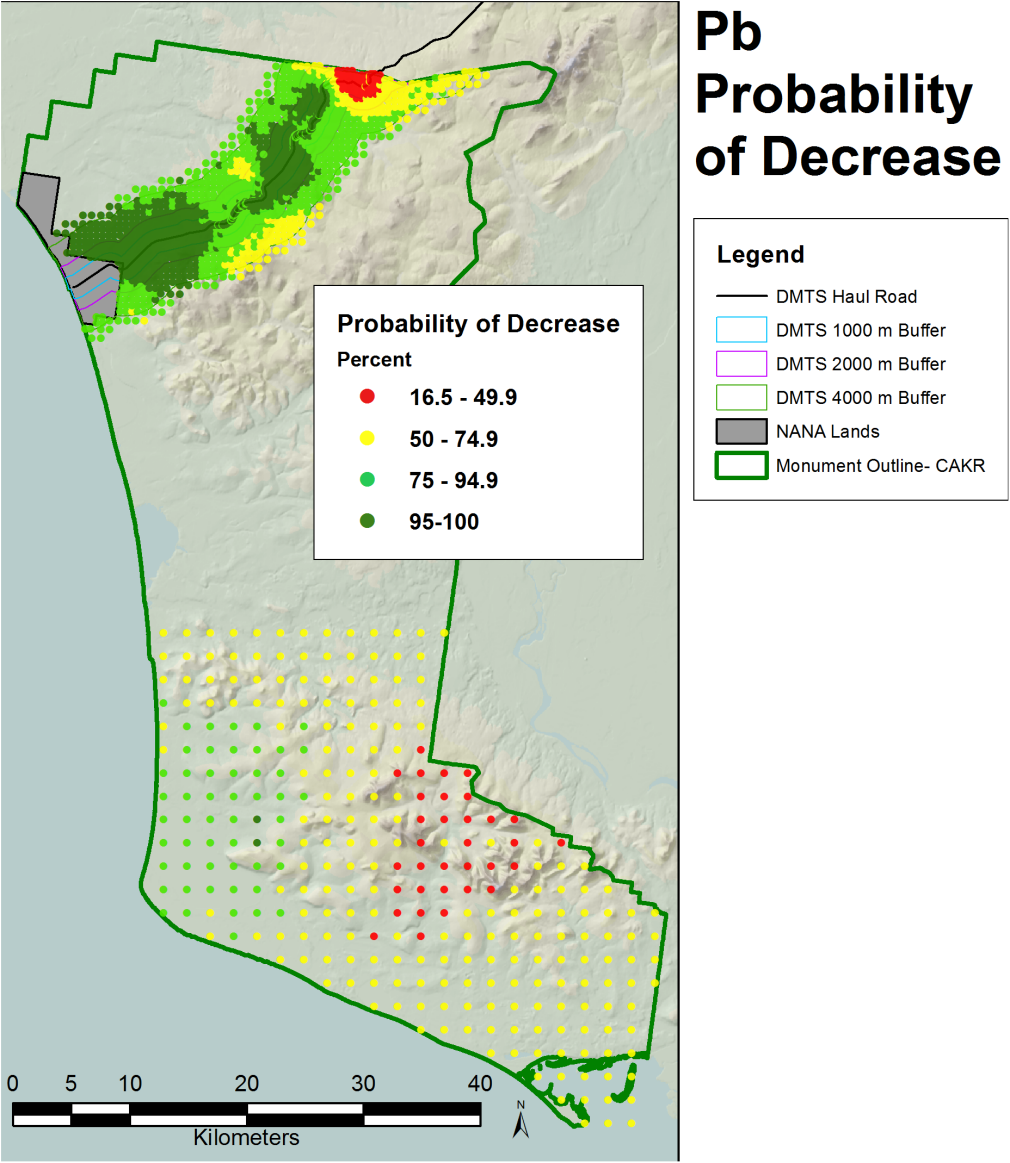
Probability of Pb decrease, 2001–2006. Probability of decrease between 2001 and 2006 Pb moss tissue concentrations based on the percentage of negative values of change among the 200 iterations at each prediction point.

**Fig 13:**
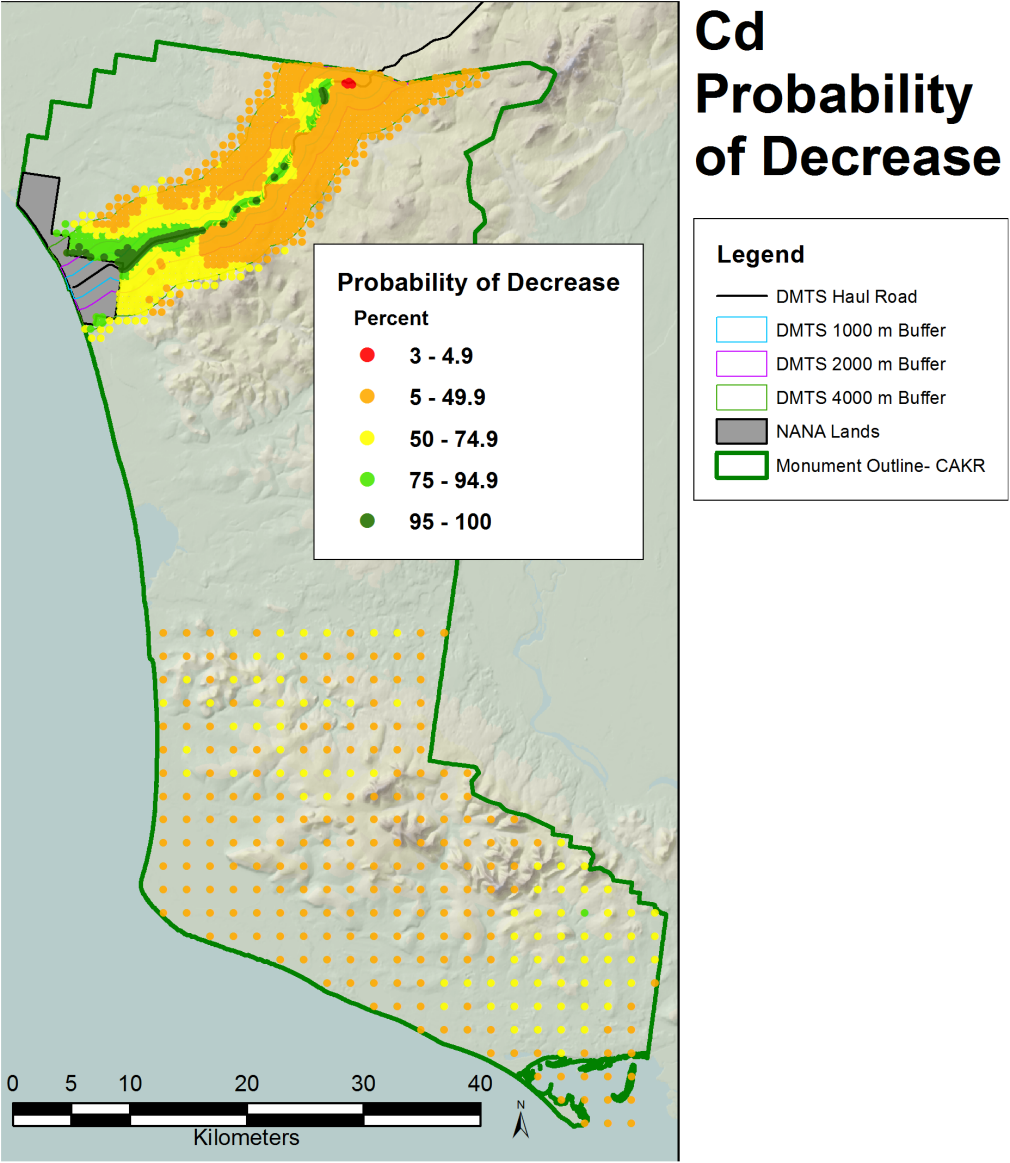
Probability of Cd decrease, 2001–2006. Probability of decrease between 2001 and 2006 Cd moss tissue concentrations based on the percentage of negative values of change among the 200 iterations at each prediction point.

For Zn and Pb, the average change and the percent change values decreased gradually with distance from the road but became less predictable beyond 2,000 m. Beyond 4,000 m to the south of the DMTS, the model showed zones of mean concentration increases for all 3 elements, though these increases should be considered suggestive rather than significant based both on intermediate probabilities of decrease (see below, Figs 11–13) and because the 95% prediction interval (the values between the 2.5 and 97.5 percentiles) contained both positive and negative values of percent change. The presence of both positive and negative values in the prediction interval necessitates that the probability of decrease was <95%, the value we use to separate significant change from nonsignificant moderate or high probability of change. Cd showed the greatest concentration increases (nonsignificant) at distances close to the DMTS (>1,000 m), while for Pb and Zn, increases (nonsignificant) did not appear until >4,000 m with a few localized exceptions. Mean increases for Pb and Zn were rarely observed to the north side of the DMTS, which captures more of the total deposition. Isolines of percent change extended farther from the road near the Port Site for all elements.

The probability of decrease was mapped at the CAKR scale by assessing the percent negative change values at each point out of the 200 iterations (Figs 11–13). Pb and Zn both showed large areas with >95% probability of decrease in moss tissue concentrations, while Cd had a very narrow band with >95% probability of decrease. Cd had large areas with intermediate probabilities of decrease between 5–75%. Probabilities of decrease >95% for Cd were most pronounced closest to the Port Site and adjacent to the DMTS haul road with a slight skew of this highest probability class to the north of the road.

The 2.5^th^ and 97.5^th^ percentiles for the four primary metrics (mean values in 2001 and 2006, average change, percent change) show expectedly divergent outcomes from the estimates of the means for these metrics (Figs 5–10, S1–S18 Figs). While the 2.5^th^ percentiles show a much cleaner study area and far higher percentages of decrease between 2001 and 2006, the 97.5^th^ percentile shows much higher levels of pollution and frequently increased loads in 2006 in more distant strata. We performed a model check and validation by plotting empirical tissue concentration observations on top of predictions for each element and have presented the results for Zn (S19 and S20 Figs). As expected, the 2001 and 2006 tissue concentrations for all elements matched the predicted surfaces well.

#### Stratum-based summarization

Predictions were aggregated (averaged) by stratum and side of road using Eq 3, and average change, percent change, and probability of decrease computed for the stratum as a whole (Tables 5–7). The results are consistent with the pointwise mapping. For all 3 elements, high and significant decreases in moss tissue concentration in 2006 relative to 2001 were observed immediately adjacent to the road (Stratum 1), and probabilities of increases <95% were noted in the reference areas in southern CAKR as well as Stratum 4 for Zn and Cd. The most notable decreases in moss elemental concentrations occurred in Stratum 1. Between 2001 and 2006, Zn decreased most dramatically among the 3 elements, declining 666 mg/kg from 1230 mg/kg in 2001 to 563 mg/kg in 2006. During this period, Pb decreased by 167 mg/kg to 199 mg/kg while Cd also decreased by 3.2 mg/kg to 5.25 mg/kg. The percent decrease in concentration was similar for the 3 elements, reducing Zn, Pb and Cd concentrations to 45%, 55% and 62% of 2001 levels, respectively. Summarized for both sides of the road, Pb decreased by 40–46% in Strata 1–4, with a 100% probability of decrease. (Please note that these probabilities are not the same as p values typically presented in statistical tests of significance—i.e., the probability of an observed outcome arising by chance—but rather the simple percentage of negative change values observed among the 200 simulation means in each stratum.) Pb showed a moderately high probability (85%) of a decrease, with the decrease averaging 0.4 mg/kg (or -18% change) in Stratum 5. Zn showed similar decreases from 17 to 54% in Strata 1–4 (with probabilities of decrease 92–100%), but little trend in Stratum 5 (a 58% probability of an increase averaging 19%). Cd again presented different overall patterns. In Stratum 1, Cd decreased by 38% (with a 100% probability of decrease). In Stratum 2, Cd showed a fairly high probability (89%) of a decrease averaging 16% north of the haul road and a 70% chance of an increase averaging 6% south of the haul road. In Strata 3–5, Cd had a 56–81% probability of an increase averaging 5–7%. Cd had its highest likely percent increase (an 85% probability of an increase averaging 14%) in Stratum 3 south of the haul road.

**Table 5:** Modeled concentrations (mg/kg dry weight) of Cd in *Hylocomium splendens* moss tissue in 2001 and 2006, average change, percent change, and probability of decrease for 5 strata.

Cd																		
**ALL**		**2001**				**2006**				**Average Change**				**Percent Change**				**Pr. Dec**
Stratum	N	**Mean**	SD	2.5 PCTL	97.5 PCTL	**Mean**	SD	2.5 PCTL	97.5 PCTL	**Mean**	SD	2.5 PCTL	97.5 PCTL	**Mean**	SD	2.5 PCTL	97.5 PCTL	%
1	697	**8.46**	0.63	7.32	9.83	**5.25**	0.41	4.51	6.18	**-3.21**	0.76	-4.82	-1.64	**-38**	7.0	-50	-21.0	**100**
2	743	**1.08**	0.05	0.99	1.18	**0.98**	0.05	0.89	1.09	**-0.09**	0.07	-0.24	0.04	**-8.4**	6.4	-21	4.3	**89**
3	507	**0.54**	0.03	0.49	0.60	**0.56**	0.04	0.49	0.64	**0.02**	0.05	-0.08	0.12	**4.8**	9.6	-13	24.1	**32**
4	121	**0.46**	0.03	0.41	0.51	**0.49**	0.04	0.42	0.59	**0.03**	0.05	-0.05	0.15	**6.7**	10.9	-11	31.3	**29**
5	289	**0.14**	0.02	0.12	0.18	**0.15**	0.02	0.11	0.20	**0.01**	0.03	-0.04	0.06	**5.5**	19.8	-25	49.9	**44**
**NORTH**		**2001**				**2006**				**Average Change**				**Percent Change**				**Pr. Dec**
Stratum	N	**Mean**	SD	2.5 PCTL	97.5 PCTL	**Mean**	SD	2.5 PCTL	97.5 PCTL	**Mean**	SD	2.5 PCTL	97.5 PCTL	**Mean**	SD	2.5 PCTL	97.5 PCTL	%
1	351	**9.96**	0.76	8.49	11.49	**6.43**	0.68	5.29	8.03	**-3.53**	1.04	-5.52	-1.37	**-35**	8.7	-50	-14.9	**100**
2	360	**1.43**	0.08	1.27	1.58	**1.19**	0.08	1.04	1.35	**-0.24**	0.11	-0.47	-0.02	**-16**	7.2	-31	-1.8	**100**
3	215	**0.70**	0.06	0.59	0.83	**0.68**	0.06	0.56	0.81	**-0.02**	0.09	-0.19	0.13	**-2.6**	12.2	-25	22.6	**60**
4	46	**0.56**	0.05	0.47	0.68	**0.58**	0.08	0.45	0.78	**0.02**	0.10	-0.15	0.26	**4.3**	17.8	-23	53.7	**45**
**SOUTH**		**2001**				**2006**				**Average Change**				**Percent Change**				**Pr. Dec**
Stratum	N	**Mean**	SD	2.5 PCTL	97.5 PCTL	**Mean**	SD	2.5 PCTL	97.5 PCTL	**Mean**	SD	2.5 PCTL	97.5 PCTL	**Mean**	SD	2.5 PCTL	97.5 PCTL	%
1	346	**6.94**	0.94	5.24	8.80	**4.05**	0.42	3.35	4.95	**-2.89**	1.03	-4.80	-0.80	**-41**	10.6	-56	-13.0	**100**
2	383	**0.75**	0.04	0.68	0.83	**0.80**	0.06	0.70	0.90	**0.05**	0.07	-0.09	0.19	**6.4**	10.2	-11	27.5	**30**
3	292	**0.42**	0.02	0.38	0.48	**0.48**	0.04	0.40	0.56	**0.06**	0.05	-0.04	0.15	**14**	12.4	-10	40.1	**15**
4	75	**0.40**	0.03	0.35	0.46	**0.43**	0.04	0.35	0.53	**0.04**	0.05	-0.08	0.13	**10**	13.2	-17	34.6	**25**
5	289	**0.14**	0.02	0.12	0.18	**0.15**	0.02	0.11	0.20	**0.01**	0.03	-0.04	0.06	**5.5**	19.8	-25	49.9	**44**

Results were tabulated for the whole data set and on both the north and south sides of the road. The final column (Pr. Dec) presents the probability of decrease in concentration between 2001 and 2006 based on the percent of simulations in which the Average Change values were negative.

**Table 6:** Modeled concentrations of Pb (mg/kg dry weight) in *Hylocomium splendens* moss tissue in 2001 and 2006, average change, percent change, and probability of decrease for 5 strata.

Pb																		
**ALL**		**2001**				**2006**				**Average Change**				**Percent Change**				**Pr. Dec**
Stratum	N	**Mean**	SD	2.5 PCTL	97.5 PCTL	**Mean**	SD	2.5 PCTL	97.5 PCTL	**Mean**	SD	2.5 PCTL	97.5 PCTL	**Mean**	SD	2.5 PCTL	97.5 PCTL	%
1	697	**366**	38	299	457	**199**	25	153	256	**-167**	46	-255	-84	**-45**	8.9	-61	-26	**100**
2	743	**38**	1.8	35	42	**21**	1.4	18	24	**-18**	2.1	-22	-13.6	**-46**	4.1	-54	-38	**100**
3	507	**18**	1.0	16	20	**10**	0.9	8.6	12	**-8**	1.4	-10	-5.0	**-44**	6.2	-54	-30	**100**
4	121	**14**	0.8	13	16	**9**	0.9	6.8	11	**-6**	1.2	-8	-3.2	**-40**	7.4	-52	-23	**100**
5	289	**1.9**	0.2	1.6	2.5	**1.5**	0.3	1.0	2.5	**-0.4**	0.4	-1	0.6	**-18**	22	-49	39	**85**
**NORTH**		**2001**				**2006**				**Average Change**				**Percent Change**				**Pr. Dec**
Stratum	N	**Mean**	SD	2.5 PCTL	97.5 PCTL	**Mean**	SD	2.5 PCTL	97.5 PCTL	**Mean**	SD	2.5 PCTL	97.5 PCTL	**Mean**	SD	2.5 PCTL	97.5 PCTL	%
1	351	**411**	44	334	496	**249**	41	186	349	**-162**	62	-283	-40	**-39**	12	-59	-9.9	**99**
2	360	**53**	3.2	47	59	**28**	2.4	23	33	**-25**	3.9	-33	-19	**-47**	5.3	-58	-38	**100**
3	215	**26**	2.1	22	30	**13**	1.6	10	17	**-13**	2.8	-18	-6.8	**-49**	8.2	-62	-29	**100**
4	46	**21**	1.8	17	25	**11**	1.8	7.9	15	**-10**	2.5	-15	-4.8	**-47**	9.7	-63	-25	**100**
**SOUTH**		**2001**				**2006**				**Average Change**				**Percent Change**				**Pr. Dec**
Stratum	N	**Mean**	SD	2.5 PCTL	97.5 PCTL	**Mean**	SD	2.5 PCTL	97.5 PCTL	**Mean**	SD	2.5 PCTL	97.5 PCTL	**Mean**	SD	2.5 PCTL	97.5 PCTL	%
1	346	**321**	56	226	445	**148**	21	115	195	**-173**	58	-306	-71	**-52**	10	-70	-29	**100**
2	383	**25**	1.5	22	28	**14**	1.4	12	18	**-11**	2.1	-15	-6.2	**-42**	6.7	-54	-27	**100**
3	292	**12**	0.6	11	13	**7.6**	0.8	6.4	9.4	**-4**	1.0	-6	-2.1	**-35**	7.6	-47	-19	**100**
4	75	**10**	0.7	8.9	12	**7.1**	0.9	5.3	9.2	**-3**	1.1	-5	-0.5	**-30**	10	-47	-5.1	**100**
5	289	**1.9**	0.2	1.6	2.5	**1.5**	0.3	1.0	2.5	**-0.4**	0.4	-1	0.6	**-18**	22	-49	39	**85**

Results were tabulated for the whole data set and on both the north and south sides of the road. The final column (Pr. Dec) presents the probability of decrease in concentration between 2001 and 2006 based on the percent of simulations in which the Average Change values were negative.

**Table 7:** Modeled concentrations of Zn (mg/kg dry weight) in *Hylocomium splendens* moss tissue in 2001 and 2006, average change, percent change, and probability of decrease for 5 strata.

Zn																		
**ALL**		**2001**				**2006**				**Average Change**				**Percent Change**				**Pr. Dec**
Stratum	N	**Mean**	SD	2.5 PCTL	97.5 PCTL	**Mean**	SD	2.5 PCTL	97.5 PCTL	**Mean**	SD	2.5 PCTL	97.5 PCTL	**Mean**	SD	2.5 PCTL	97.5 PCTL	%
1	697	**1230**	81	1097	1409	**563**	50	471	674	**-666**	96	-858	-490	**-54**	5.1	-64	-43	**100**
2	743	**188**	6.8	175	202	**99**	5.9	87	113	**-88**	8.5	-105	-71	**-47**	3.5	-53	-40	**100**
3	507	**99**	4.2	93	109	**68**	5.5	59	82	**-31**	7.1	-44	-16	**-31**	6.4	-42	-17	**100**
4	121	**84**	3.6	77	92	**70**	8.5	55	87	**-15**	9.3	-32	5.2	**-17**	11	-36	6	**92**
5	289	**41**	3.6	35	48	**49**	20	26	97	**7.6**	20	-18	61	**19**	50	-42	161	**42**
**NORTH**		**2001**				**2006**				**Average Change**				**Percent Change**				**Pr. Dec**
Stratum	N	**Mean**	SD	2.5 PCTL	97.5 PCTL	**Mean**	SD	2.5 PCTL	97.5 PCTL	**Mean**	SD	2.5 PCTL	97.5 PCTL	**Mean**	SD	2.5 PCTL	97.5 PCTL	%
1	351	**1490**	106	1307	1702	**724**	80	578	931	**-766**	141	-1033	-490	**-51**	6.7	-62	-36	**100**
2	360	**244**	12	221	269	**122**	10	105	145	**-121**	15	-149	-94	**-50**	4.6	-58	-41	**100**
3	215	**128**	8.6	115	145	**74**	9.0	57	97	**-54**	13	-77	-28	**-42**	8.2	-56	-23	**100**
4	46	**107**	7.1	95	123	**70**	13	48	96	**-38**	15	-63	-4.4	**-35**	13	-54	-4.3	**99**
**SOUTH**		**2001**				**2006**				**Average Change**				**Percent Change**				**Pr. Dec**
Stratum	N	**Mean**	SD	2.5 PCTL	97.5 PCTL	**Mean**	SD	2.5 PCTL	97.5 PCTL	**Mean**	SD	2.5 PCTL	97.5 PCTL	**Mean**	SD	2.5 PCTL	97.5 PCTL	%
1	346	**965**	118	783	1226	**400**	44	324	505	**-565**	123	-846	-357	**-58**	6.5	-70	-45	**100**
2	383	**136**	5.8	126	147	**78**	6.7	66	93	**-57**	8.5	-75	-41	**-42**	5.3	-52	-32	**100**
3	292	**78**	3.3	72	86	**64**	6.3	53	76	**-15**	7.2	-30	-0.2	**-18**	8.8	-36	-0.3	**99**
4	75	**70**	3.3	64	77	**70**	10	53	91	**-0.5**	11	-20	23	**-0.4**	16	-27	34	**56**
5	289	**41**	3.6	35	48	**49**	20	26	97	**7.6**	20	-18	61	**19**	50	-42	161	**42**

Results were tabulated for the whole data set and on both the north and south sides of the road. The final column (Pr. Dec) presents the probability of decrease in concentration between 2001 and 2006 based on the percent of simulations in which the Average Change values were negative.

While both the lower and upper bounds of the 95% prediction interval contained all negative values of change for Stratum 1 (all elements), Strata 2–3 (Pb, Zn) and Strata 4 (Pb), instances of both positive and negative change values within this prediction interval occurred in the strata farther from the road (Cd: Strata 2–5, Pb: Stratum 5, and Zn: Strata 4–5). The greatest certainties accompanied the two-thirds of the probability of decrease values of that attained 100% and several other high probability values with entirely negative upper and lower bounds (Tables 5–7).

The mean 2006 concentrations of Zn, Pb and Cd on NPS lands were mapped with an overlay of the NANA industrial easement (S21–S23 Figs). The highest modeled concentrations of these elements in moss tissue on NPS lands outside the easement were 263 mg/kg of Zn, 53 mg/kg of Pb, and 1.6 mg/kg of Cd. These values were considerably higher than those found in the reference areas of southern CAKR, and between 7 and 88 times the concentrations of mean Alaska background levels [6]. NPS lands north of the Port Site had the highest concentrations outside of the easement, followed by lands south of the Port Site and then by lands immediately adjacent to the easement (both north and south).

### Maxima and minima in raw data

The 2001 and 2006 ranges of Cd, Pb, and Zn in the tissue data set (unmodeled) are presented in Table 8 alongside earlier ranges from the Arctic Contaminants Research Program [6]. The 2006 maxima for these three elements (24, 301, and 993 mg/kg respectively) were generally about one-third of 2001 values. The 2006 minima were slightly higher than the 2001 minima for Cd and Zn, and slightly lower for Pb. Notably, the 2006 Pb maximum dropped below the Alaska Department of Environmental Conservation’s recommendation of 400 mg/kg in soil cleanup value for residential areas and well below the 800 mg/kg in industrial areas [22].

**Table 8:** Ranges of elemental concentrations (mg/kg dry weight) in *Hylocomium splendens* moss tissue in raw (unmodeled) data, previous background study [6], and State of Alaska cleanup levels [22].

	N	Cd	Pb	Zn
***All Strata***				
2001 All Samples	219	0.08–24.3	1.1–918	24.6–3442
2006 All Samples	125	0.13–8.6	0.8–301	28.4–993
***Strata 1–4***				
2001 Stratum 1	23	1.65–24.3	69.2–918	261–3442
2001 Stratum 2	53	0.38–6.89	11.1–263	71.9–1136
2001 Stratum 3	32	0.18–2.22	5.5–91.2	43.9–377
2001 Stratum 4	17	0.30–1.11	6.2–54.2	54.2–218
2006 Stratum 1	38	1.74–8.60	35.4–300	123–993
2006 Stratum 2	47	0.32–4.67	5.0–143	36.7–452
2006 Stratum 3	24	0.32–1.40	3.9–23.6	50.1–165
2006 Stratum 4	4	0.31–0.79	6.3–18.6	41.8–72.7
***Stratum 5 (Reference)***				
2001 Reference Sites^a^	13	0.09–0.22	1.1–2.1	24.6–56.6
2006 Reference Sites	12	0.13–0.30	0.8–5.7	28.4–74.2
***Arctic Contaminants Research Program***				
1990–1992 Arctic Alaska Background^b^ [6]	32	0.02–0.98	0.4–2.3	10.4–66.3
1990–1992 Arctic Alaska Background Median^b^ [6]	32	0.15	0.6	37.4
***State of Alaska Cleanup Levels***				
Alaska Department of Environmental Conservation Cleanup Level^c^ [22]		110	400-Residential 800-Industrial	41,000

2001 raw data [1] were assigned to current study’s model strata groups based on distance from DMTS haul road. Reference sites for 2001 were assigned for this table based on their geographic location (see footnote a. below). N is the number of samples.

a. 13 samples from 7 southernmost sites in 2001 sampling [1] from southern CAKR (Fig 2), >40 km from DMTS haul road, in vicinity of 2006 reference sites, and further south than the northernmost 2006 reference site.

b. 24 sites from US Environmental Protection Agency’s Arctic Contaminant Research Program [6] between 1990 and 1992.

c. Alaska Department of Environmental Conservation 18 AAC 75, Table B1, Method Two—Soil Cleanup Levels, Arctic Zone/Human Health. Lead cleanup levels are based on land use; for residential land use, the soil cleanup level is 400 mg/kg. For commercial or industrial land use, as applied in 18 AAC 75.340(e)(3), the soil cleanup level is 800 mg/kg; through an approved site-specific risk assessment.

### Differences between North and South sides of road

The differences in Zn, Pb and Cd concentrations between the north and south side of the road observed for the 2001 modeled data (Tables 5–7) and in Hasselbach et al. [1] were still evident in the 2006 data. In 2006, the modeled moss tissue concentrations on the north side of the road exceeded those in the south side in every stratum for every element except for one which was equal. Within-strata mean modeled moss tissue concentrations ranged from 0 to 100% greater on the north side in Strata 1–4 with an average of 53% for all elements in all strata (S1 Table). The mean percentage by which concentrations-within-strata on the north side of the road exceeded those on the south side ranged from 74% (range 55–100%) for Pb to 46% (range 35–59%) for Cd and 38% (range 0–81%) for Zn. The 2006 elemental concentration differences between north and south were generally highest in Strata 1 and 2.

The comparison of percent change over time by side of road told a different story. Percent decreases were higher on the south side in Stratum 1 for all elements. Because the 95% credibility interval spanned zero in average change for some element-strata-side of road combinations, the change in north and south sides over time and the percent exceedance of north-to-south sides contain moderate to high levels of uncertainty for these combinations (S1 Table). For those combinations in Strata 2–4 in which the 95% credibility intervals were entirely negative for average change, both Zn and Pb concentrations decreased more on the north side than the south between 2001 and 2006 (12–57% for Pb and 19–133% for Zn). For strata in which the 95% credible interval for average change did not span zero, the percentage decrease between 2001 and 2006 ranged from –42 to –51% (N) vs –18 to– 58% (S) for Zn, and –39 to –47 (N) vs. –30 to –52 (S) for Pb.

In Stratum 1, the mean north:south ratio of pooled moss tissue concentrations of Zn, Pb and Cd was 1.5 in 2001 and 1.8 in 2006 (S2 Table). This pattern of increase was reversed for strata 2 through 4. In these strata, the north:south ratio, while still above 1 (ranging from 1.1 to 1.6) decreased in 2006 compared to 2001. In sum, for Stratum 1 while elemental concentrations were much lower in 2006 than in 2001, the north side improved less than the south side. For strata 2–4, the north side improved more than the south side.

## Discussion

### Changes in deposition patterns

There was a substantial reduction of dust-borne contaminant deposition between 2001 and 2006, and reductions were greatest in areas where heavy metals concentrations were previously highest–Stratum 1, the area surrounding the Port Site, and the north side of the road (Figs 8–10, S4, S11 and S18 Figs). The several miles surrounding the Port Site had previously been a high deposition zone because standard operations generally left the doors of the Concentrate Storage Buildings open prior to 2001, thus allowing the steady winds to disperse 55% Zn and Pb concentrates onto the surrounding tundra [3]. This is reflected in the mine’s 2003 sampling of NANA-owned lands adjacent to the Port Site, which revealed moss tissue concentrations at the Port Site of 1,720 mg/kg of Pb and 8,120 mg/kg of Zn [3]. Vegetation at the port site was reported in 2003 to be highly stressed and/or dead. Tundra soils in this area reached maxima for Cd, Pb and Zn of 3,600, and 11,500 and 15,000 mg/kg, respectively [3]. The north side of the DMTS haul road had previously accumulated high deposition because of the prevailing winter easterly winds that carry road dust preferentially to the north of the road during the 8 winter months. Concentration contours of all three contaminants still showed a moderate bulge near the Port Site in 2006, but much of the area in this zone had dropped into lower tissue concentration classes than in 2001, presumably due to dust control measures (see below).

Generally, the initially high decreases in modeled tissue concentrations between 2001 and 2006 lessened with distance from the road, and Cd and Zn both eventually showed moderate (though nonsignificant at the 95% level) probabilities of positive percent change with increasing distance. While the diminishing decreases in concentrations occurred in strata 2–4 for Zn and Pb, Cd showed no significant trends beyond Stratum 1 and moderate probabilities (56–85%) of increase in strata 3–5 on the south side of the road. The reasons for these increases in Cd—if real—are unknown; possibilities could include a greater mobility of Cd relative to Zn or Pb, changes in the Cd concentrations in the Zn and Pb concentrates over time, or other factors. In 2003, Cd represented 0.12% of the Pb concentrate and 0.33% of the Zn concentrate (Exponent 2007), thus production differences could potentially play a role.

Spatial variability of modeled concentrations was low in Stratum 1 with the exception of one area of increase near the northern boundary of CAKR, which attained percent change values up to +18% (with probabilities of increase from 51–97%). Convincing support for this hotspot comes from probabilities of increase: Cd showed a probability of increase of ≥95%, while Pb and Zn ranged from 49–83% and 51–74% respectively, with the highest probabilities immediately adjacent the road. It is conceivable that this increase is a relict of a 1992 Pb concentrate spill of 15 tons [23] and/or a 2004 spill of 1.4 tons of gravel contaminated with concentrate that occurred over a two mile stretch of the haul road in this vicinity. Both 2001 and 2006 sampling points fell within 300 m of these spill sites, thus the potential to detect them was higher than at other spill sites without sample points in close proximity. Spatially intensive sampling of former spill sites would be required to determine whether residues from these incidents have led to ongoing heavy metals dispersal.

### Differences between North and South sides of road

Several factors favor greater dust dispersal and long-distance transport on the north side of the road than the south. First, the topography on the north side is generally flatter than the rolling landforms on the south. Second, the terrain generally drops in elevation to the north and rises fairly rapidly to the south. Third, the easterly winds that prevail during the 8 winter months tend to transport particles to the north for more of the time in a given year. Not surprisingly, the differences between elemental concentrations on the north vs. south side of the road noted in 2001 (Hasselbach et al. 2005) persisted in the 2006 data. For Stratum 1, the north to south differences in elemental concentration increased in 2006 from their 2001 levels for all elements both individually (S1 Table) and pooled (S2 Table). The opposite occurred in more distant strata though ratios remained above 1:1 (S2 Table). Theoretically, if the heavy metal deposition decreased toward zero, the N/S ratios at some future date would approach 1. The decrease in values of Strata 2 through 4 could signal exactly this sort of improvement trend. As the stratum with the highest Zn, Pb and Cd concentrations in the study area, Stratum 1 may receive too much ongoing localized contamination (and especially on the north side) to show any improvement in the north relative to the south. Dust control measures may have the potential to reduce the north:south differences in Stratum 1 over time: if dust-control measures continue a general abatement of releases, the north side should see more improvement, which could help equalize the north and south sides in Stratum 1.

### Technical improvements

Beginning in 2001, the Red Dog Mine made a number of technical alterations to its operations in order to reduce the amount of fugitive dusts escaping to the environment [24]. Among these measures were: replacing the tarp-covered concentrate haul trucks with a new fleet of trucks with hydraulically-covered lids, implementing a seasonal truck rinse station for use in summer months, keeping the large doors of the concentrate storage building doors closed by constructing enclosed unloading facilities, constructing negative-pressure bag houses in the concentrate loading and unloading facilities, enclosing the conveyor system that moves concentrates from the concentrate storage buildings to vessels at the Port Site, applying dust palliatives to the roadbed, and segregating road traffic to reduce concentrate tracking by vehicles. The noted decreases in fugitive dust deposition cannot be attributed to decreases in production during the 2001–2006 period. In 2001, the mine shipped 1.2 million metric tons of lead and zinc concentrates (pers. comm. Teck, Inc; S2 File). The quantities rose from this starting value by approximately 130,000–175,000 tons during the following 5 years.

Investments in road dust control and other emissions mitigation at the Red Dog Mine Site led to analogous reductions in fugitive dust emissions there. In an evaluation of heavy metals emissions at the mine site (about 30 miles from the CAKR boundary), SENES [24, 25] found that factors such as road dust control and a deepening pit that better contained emissions were key in these reductions. Only one period of slight increase of fugitive dust emissions at the mine site was noted (from 2004–2005), which appeared to correspond to increased truck traffic to transport a growing body of waste rock from the mine pit to a storage area nearby [25].

### Concentrations in Southern CAKR

There were no clear trends in deposition patterns of Zn, Pb or Cd in the reference area of southern CAKR (Stratum 5). The average and percent change values for Cd and Zn in Stratum 5 had only a 54% and 48% chance of positive change for Cd and Zn, respectively. Pb had an 85% probability of an 18% decrease, with a change from 1.9 mg/kg to 1.5 mg/kg. Uncertainty in these results stems partly from the large area included in the model, the variability of the sample concentrations, the relatively low sample size, and the low contaminant concentrations. Our main finding for Stratum 5 is that while no trend is evident, this area is still experiencing low levels of ongoing deposition of Zn and Pb. While concentrations in southern areas are still fairly low, concentrations for Pb and Zn remained above the arctic baseline values reported in Ford et al. [6]. The 2006 mean modeled concentrations of Pb and Zn were 1.5 and 49 mg/kg respectively, compared to Ford et al.’s Alaska background values of 0.6 and 37 mg/kg respectively. The 2006 value for Cd in this study (0.15 mg/kg) matched Ford et al.’s Alaska background value. The variability among raw samples was surprisingly high for the reference area. 2006 tissue samples ranged from 28 to 74 mg/kg for Zn, 0.8 to 5.7 mg/kg for Pb, and 0.13 to 0.30 mg/kg for Cd. The mean of the raw values of Cd (0.18 mg/kg) was slightly higher than the modeled value and Ford et al.’s background. There are no baseline measurements of the reference area prior to the mine’s opening in 1989, therefore natural sources of deposition such as weathering of mineralized outcrops cannot be ruled out. Elemental fingerprinting (e.g., via isotopes, speciation) could help to establish the source more definitively [26, 27].

There does not appear to be any obvious geographic basis to explain the variability in Zn, Pb or Cd concentrations or spatial patterns within the reference area (Stratum 5). Moreover, it is also conceivable that deposition of Zn, Pb and Cd in Stratum 5 decreased between 2001 and 2006 but that this change was too small to be reflected in the 3–5 year moss tissue sampled. Mosses are highly adapted to retain atmospheric deposition, especially at low levels of deposition beneath the higher thresholds at which they reach saturation or begin to leak. Brumelis and Brown [28] reported that heavy metals can be mobile between older and younger segments within *H*. *splendens* tissue and that metals can migrate from higher concentration source areas to lower concentration sink areas. Brumbaugh et al. [29] found significant differences in elemental accumulation between first year and 3–5 year tissue of *H*. *splendens*, with higher metals concentrations in the older tissue. Unlike the stressed feather mosses adjacent to the haul road, reference area mosses tended to retain green (and presumably absorptive) tissue in the range of 6–8 years before beginning to decompose and release retained elements. In environments with low inputs of heavy metals, high retention and within-tissue redistribution may make detection of short term temporal concentration changes more challenging. Small but consistent quantities of laboratory error can also lead to larger proportional error in tissue with low concentrations. Likewise, trend assessment in low deposition environments is inherently challenging because small changes in concentration represent a much higher proportional change than in areas of higher deposition.

In interpreting percent change maps for low deposition environments, it is important to consider that small changes of only a few mg/kg in actual concentrations can at times appear inflated in importance in percent change mapping because of low starting values. In areas of higher starting deposition, even lower percentage change values represent higher changes in actual concentrations. Maps of actual differences in concentration (Average Change) are presented to provide a cross-check on the importance of the changes presented (Fig 7, S3, S7, S10, S14 and S17 Figs).

### Environmental fate of contaminants

Moss biomonitoring enables an integration of moss accumulation of contaminants during a narrow window. The current analysis pertains only to contaminants in and on moss within a 3–5 year sampling period. The fugitive dusts that either deposited earlier than this timeframe, or dusts that previously washed into the organic soil profile over the past 24 years are likely to have resulted in a considerable pool of residual Zn, Pb and Cd. Accumulated contaminants in soils can be further dispersed by wind and storm events. This redistribution of sediments and contaminants will be particularly pronounced in low-precipitation environments such as this, characterized by sparse vegetation and low surface moisture during much of the year [30, 31]. Smaller particles will be more mobile and carried further away from the source. Mining-associated contaminants have been reported to be deposited kilometers to hundreds of kilometers from source previously [32, 31]. For small particle sizes such as those of the ore concentrates, factors important in determining metal fate will include wind-driven transport near the soil surface, surface creep and saltation, as well as suspension [32]. This study and Hasselbach et al. (2005) [1] provide evidence for the distance over which contaminants can be transported, with elevated levels observed across the studied northern section of the CAKR, up to 4 km from the road, and low levels of contaminants found tens of kilometers from the road in the southern CAKR (discussed above).

The Pb and Zn-enriched dusts deposited on the tundra initially contain these metals in reduced sulfide forms (Exponent 2007), which tend to exhibit low bioaccessibility [33]. Over time chemical weathering and exposure to air will oxidize these contaminants to more bioavailable forms. A 2007 risk assessment [3] assumed 100% bioavailability of a large suite of contaminants and concluded that the risks to a wide range of wildlife, plants and humans via ingestion and direct contact were to: 1) small mammals from elevated boron; 2) Willow Ptarmigan (*Lagopus lagopus*) from elevated Cd near the mine site (outside of CAKR); and 3) the cover of lichens and bryophytes out to 2,000 m from the haul road. Sublethal effects that could influence population numbers and health were not considered, however, and it is possible that the below-ground contaminant reservoir could contribute to these types of effects for a range of ecological receptors that live in or eat soil and vegetation and then introduce them into the broader food web [34]. Because of the early history of fugitive dust dispersion, the soil-based pool of contaminants could persist for many decades even if no further release of fugitive dusts occurred. The active layer of the soil profile is generally less than 1 m deep above permafrost in CAKR [13]. This suggests that contaminants have the potential to be trapped in the organic soil horizons unless released into perched water as they become more bioavailable. Within this century, Arctic warming is projected to deepen the active layer and warm permafrost temperatures to the thaw point in northern CAKR [35]. This is likely to bring changes in the hydrological regime and organic carbon fluxes that have the potential to change the rate of transfer of legacy contamination to surface and ground waters.

An area over 100 km^2^ in CAKR (approximately 2000 m on each side of the 32 km road easement) is likely to have some level of reduced lichen and bryophyte cover and extensive lichen mortality [3]. If the decreases in Zn, Pb and Cd noted in this study persist, there should be some level of lichen recovery in the more distant areas of the affected zone, though the timing and spatial patterns of recovery are difficult to predict. It is also unknown how the soil reservoir of Zn, Pb, Cd and sulfides may affect lichens with extensive underground tissue, e.g., the very common *Cladonia* spp. and *Cetraria* spp. Due to the slow growth rates of these nonvascular plants (6.2 mm/yr for *Cladonia stygia* (Fr.) Ruoss common in the study area [36], however, it is likely that their recovery will take many decades even at low future levels of contaminant input. A subsequent manuscript will use the simulations developed in the present study to make estimates of the terrestrial area above elemental concentration thresholds established for biological metrics such lichen species richness, lichen cover, lichen mortality, and the blackening of the moss *Hylocomium splendens*.

## Conclusions

The Red Dog Mine’s efforts to control fugitive dust emissions appear to have resulted in a decrease in the deposition of Zn, Pb and Cd along the DMTS corridor between 2001 and 2006. Decreases were greatest for all three elements immediately adjacent to the haul road (Stratum 1) and the Port Site. Decreases for Zn and Pb moderated with distance from the haul road, while trends for Cd were less clear beyond 100 m. Trends in the reference areas of southern CAKR were not evident. Continued monitoring is needed to determine whether this predominantly downward slope in fugitive dust deposition represents an ongoing trend.

Concentrations of Zn, Pb and Cd remain a concern both in the NANA easement and on adjacent NPS lands beyond the easement. The known biological effects of these contaminants on NPS lands include lichen mortality, decreased lichen species richness and cover, decreased bryophyte cover, blackening of *H*. *splendens*, the modeled potential for injury to small mammals adjacent to the road, and sub-lethal markers of exposure for certain birds and voles. Subsequent manuscripts based on now-completed analysis will present the spatial extent of contaminants associated with significant effects on lichens. The spatial models generated in the current study will be used to map these effects and generate area estimates. If downward trends continue or stabilize in the next monitoring round (2017), it is possible that we may begin to see lichen recovery of the areas near the edges of the contaminant effect zone (approximately 2000 m in [3]. Prediction of recovery is complicated by changing contaminant levels over time, changing spatial patterns of deposition, and a lag time in lichen recolonization, but a better estimate will be possible using continuing contaminant monitoring and the biological response thresholds generated for the 2006 data. Continuing contaminant monitoring will be important to document where new spatial patterns of deposition fall relative to biological thresholds.

The Red Dog Mine plans to continue operations for another 20 years in the current deposit, and then to move into lower grade deposits for another 10 years [5]. Because the life of the mine is so long, the continuing deposition of moderate amounts of fugitive dusts is likely to accumulate into larger pools in the organic soil horizons over time. While there has been a decrease in deposition, NPS is concerned about the potential for a growing and increasingly bioavailable soil contaminant layer. NPS is scheduled to resume active management of substantially contaminated easement lands once the easement period expires in 2084. Research on concentrations of Zn, Pb and Cd in organic soils is recommended.

## Supporting information

S1 Fig Modeled 2001 Cd moss tissue concentrations in CAKR. The 2.5^th^ and 97.5^th^ percentiles of the modeled concentrations are shown at right. Dots on the main graph are sized proportionally in four classes by the quartile distributions of the reciprocal of the CV.
(TIF)

S2 Fig Modeled 2006 Cd moss tissue concentrations in CAKR. The 2.5^th^ and 97.5^th^ percentiles of the modeled concentrations are shown at right. Dots on the main graph are sized proportionally in four classes by the quartile distributions of the reciprocal of the CV.
(TIF)

S3 Fig Average change between modeled 2001 and 2006 Cd moss tissue concentrations in CAKR. The 2.5^th^ and 97.5^th^ percentiles of the modeled concentrations are shown at right. Dots on the main graph are sized proportionally in four classes by the quartile distributions of the reciprocal of the CV.
(TIF)

S4 Fig Percent change between modeled 2001 and 2006 Cd moss tissue concentrations in CAKR. The 2.5^th^ and 97.5^th^ percentiles of the modeled concentrations are shown at right. Dots on the main graph are sized proportionally in four classes by the quartile distributions of the reciprocal of the CV.
(TIF)

S5 Fig Modeled 2001 Cd moss tissue concentrations along the DMTS. The 2.5^th^ and 97.5^th^ percentiles of the modeled concentrations are shown at right. Dots on the main graph are sized proportionally in four classes by the quartile distributions of the reciprocal of the CV.
(TIF)

S6 Fig Modeled 2006 Cd moss tissue concentrations along the DMTS. The 2.5^th^ and 97.5^th^ percentiles of the modeled concentrations are shown at right. Dots on the main graph are sized proportionally in four classes by the quartile distributions of the reciprocal of the CV.
(TIF)

S7 Fig Average change between modeled 2001 and 2006 Cd moss tissue concentrations along the DMTS. The 2.5^th^ and 97.5^th^ percentiles (lower and upper bound of the 95% interval) of the modeled concentrations are shown at right. Dots on the main graph are sized proportionally in four classes by the quartile distributions of the reciprocal of the CV.
(TIF)

S8 Fig Modeled 2001 Pb moss tissue concentrations in CAKR. The 2.5^th^ and 97.5^th^ percentiles (lower and upper bound of the 95% interval) of the modeled concentrations are shown at right. Dots on the main graph are sized proportionally in four classes by the quartile distributions of the reciprocal of the CV.
(TIF)

S9 Fig Modeled 2006 Pb moss tissue concentrations in CAKR. The 2.5^th^ and 97.5^th^ percentiles (lower and upper bound of the 95% interval) of the modeled concentrations are shown at right. Dots on the main graph are sized proportionally in four classes by the quartile distributions of the reciprocal of the CV.
(TIF)

S10 Fig Average change between modeled 2001 and 2006 Pb moss tissue concentrations in CAKR. The 2.5^th^ and 97.5^th^ percentiles (lower and upper bound of the 95% interval) of the modeled concentrations are shown at right. Dots on the main graph are sized proportionally in four classes by the quartile distributions of the reciprocal of the CV.
(TIF)

S11 Fig Percent change between modeled 2001 and 2006 Pb moss tissue concentrations in CAKR. The 2.5^th^ and 97.5^th^ percentiles (lower and upper bound of the 95% interval) of the modeled concentrations are shown at right. Dots on the main graph are sized proportionally in four classes by the quartile distributions of the reciprocal of the CV.
(TIF)

S12 Fig Modeled 2001 Pb moss tissue concentrations along the DMTS. The 2.5^th^ and 97.5^th^ percentiles (lower and upper bound of the 95% interval) of the modeled concentrations are shown at right. Dots on the main graph are sized proportionally in four classes by the quartile distributions of the reciprocal of the CV.
(TIF)

S13 Fig Modeled 2006 Pb moss tissue concentrations along the DMTS. The 2.5^th^ and 97.5^th^ percentiles (lower and upper bound of the 95% interval) of the modeled concentrations are shown at right. Dots on the main graph are sized proportionally in four classes by the quartile distributions of the reciprocal of the CV.
(TIF)

S14 Fig Average change between modeled 2001 and 2006 Pb moss tissue concentrations along the DMTS. The 2.5^th^ and 97.5^th^ percentiles (lower and upper bound of the 95% interval) of the modeled concentrations are shown at right. Dots on the main graph are sized proportionally in four classes by the quartile distributions of the reciprocal of the CV.
(TIF)

S15 Fig Modeled 2001 Zn moss tissue concentrations in CAKR. The 2.5^th^ and 97.5^th^ percentiles (lower and upper bound of the 95% interval) of the modeled concentrations are shown at right. Dots on the main graph are sized proportionally in four classes by the quartile distributions of the reciprocal of the CV.
(TIF)

S16 Fig Modeled 2006 Zn moss tissue concentrations in CAKR. The 2.5^th^ and 97.5^th^ percentiles (lower and upper bound of the 95% interval) of the modeled concentrations are shown at right. Dots on the main graph are sized proportionally in four classes by the quartile distributions of the reciprocal of the CV.
(TIF)

S17 Fig Average change between modeled 2001 and 2006 Zn moss tissue concentrations in CAKR. The 2.5^th^ and 97.5^th^ percentiles (lower and upper bound of the 95% interval) of the modeled concentrations are shown at right. Dots on the main graph are sized proportionally in four classes by the quartile distributions of the reciprocal of the CV.
(TIF)

S18 Fig Percent change between modeled 2001 and 2006 Zn moss tissue concentrations in CAKR. The 2.5^th^ and 97.5^th^ percentiles (lower and upper bound of the 95% interval) of the modeled concentrations are shown at right. Dots on the main graph are sized proportionally in four classes by the quartile distributions of the reciprocal of the CV.
(TIF)

S19 Fig Plot of empirical observations of moss tissue concentrations on top of prediction, Zn 2001. (TIF)

S20 Fig Plot of empirical observations of moss tissue concentrations on top of prediction, Zn 2006. (TIF)

S21 Fig Modeled 2006 Cd moss tissue concentrations in CAKR. The 2.5^th^ and 97.5^th^ percentiles of the modeled concentrations are shown at right. Dots on the main graph are sized proportionally in four classes by the quartile distributions of the reciprocal of the CV. The DMTS easement is plotted on top of Cd concentrations in moss.
(TIF)

S22 Fig Modeled 2006 Pb moss tissue concentrations in CAKR. The 2.5^th^ and 97.5^th^ percentiles of the modeled concentrations are shown at right. Dots on the main graph are sized proportionally in four classes by the quartile distributions of the reciprocal of the CV. The DMTS easement is plotted on top of Pb concentrations in moss.
(TIF)

S23 Fig Modeled 2006 Zn moss tissue concentrations in CAKR. The 2.5^th^ and 97.5^th^ percentiles of the modeled concentrations are shown at right. Dots on the main graph are sized proportionally in four classes by the quartile distributions of the reciprocal of the CV. The DMTS easement is plotted on top of Zn concentrations in moss.
(TIF)

S1 File Laboratory Analysis Methods Used for 2006 Moss Tissue Samples. (PDF)

S2 File Tonnes of Pb and Zn concentrate shipped by Red Dog Mine, 2001–2006. Provided by Teck, Inc., March 31, 2017.
(PDF)

S1 Table Differences between north (N) and south (S) sides of DMTS haul road in 2006 modeled elemental concentrations and % change between 2001 and 2006. % N>S represents the percentage by which the north side exceeds the south side for a given metric. Strata for which the 95% credible interval in average change spanned zero for the north or south side have higher uncertainty are shown in blue with a smaller font.
(PDF)

S2 Table Ratios of N:S side of road modeled concentrations by stratum in 2001 and 2006. Strata 5 exists only in the south, and it has no N:S ratio but is still included in the overall mean. Strata for which the 95% credible interval in average change spanned zero for the north or south side have higher uncertainty are shown in blue with a smaller font.
(PDF)
