# Supplementary figures and images for: Trends in spatial patterns of heavy metal deposition on national park service lands along the Red Dog Mine haul road, Alaska, 2001–2006
Source: PLoS One. 2017 May 18;12(5):e0177936. doi: 10.1371/journal.pone.0177936 (PMC5436859; doi:10.1371/journal.pone.0177936)

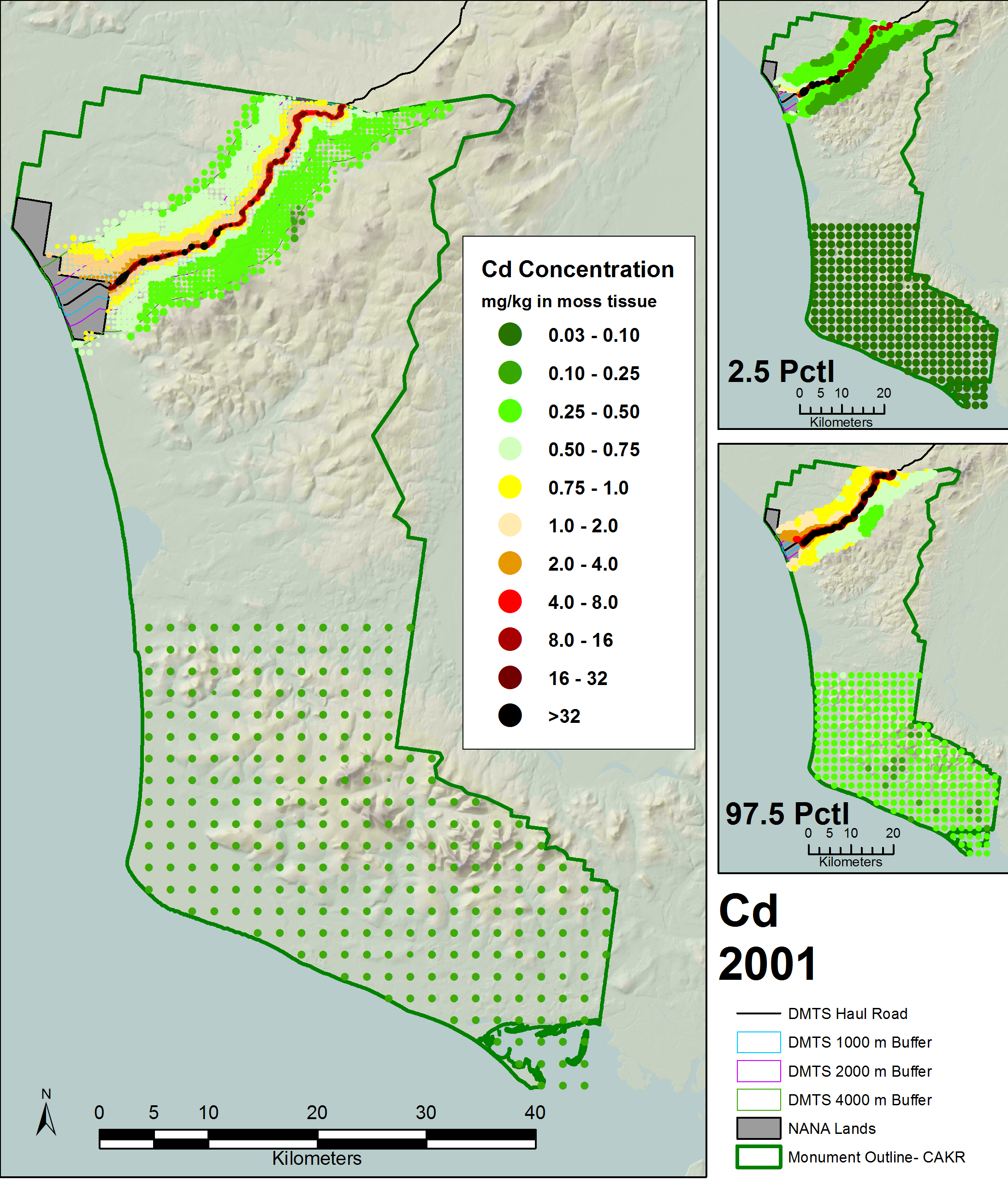

Supplement: S1 Fig — The 2.5th and 97.5th percentiles of the modeled concentrations are shown at right. Dots on the main graph are sized proportionally in four classes by the quartile distributions of the reciprocal of the CV. (TIF) [file pone.0177936.s001.tif]

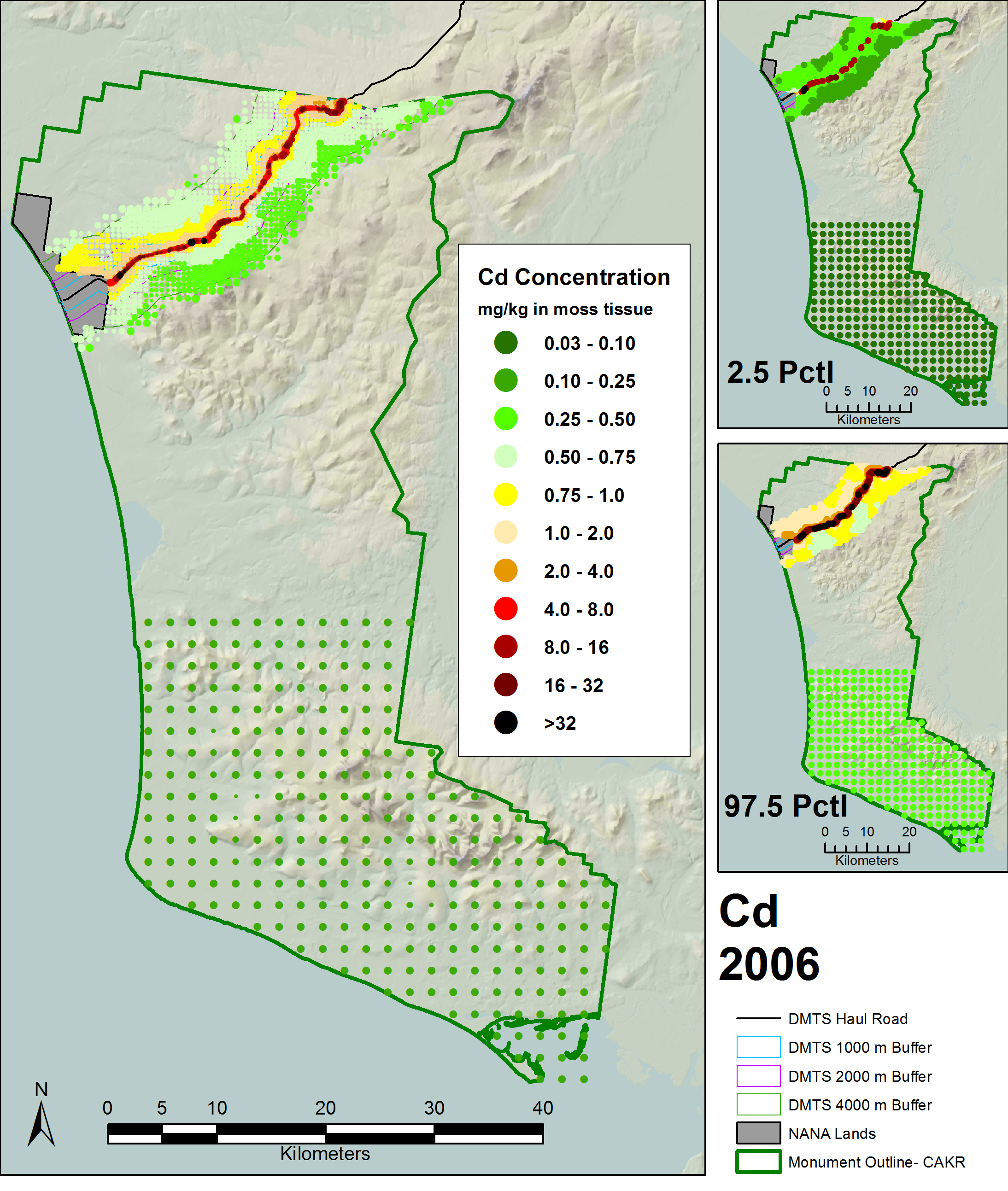

Supplement: S2 Fig — The 2.5th and 97.5th percentiles of the modeled concentrations are shown at right. Dots on the main graph are sized proportionally in four classes by the quartile distributions of the reciprocal of the CV. (TIF) [file pone.0177936.s002.tif]

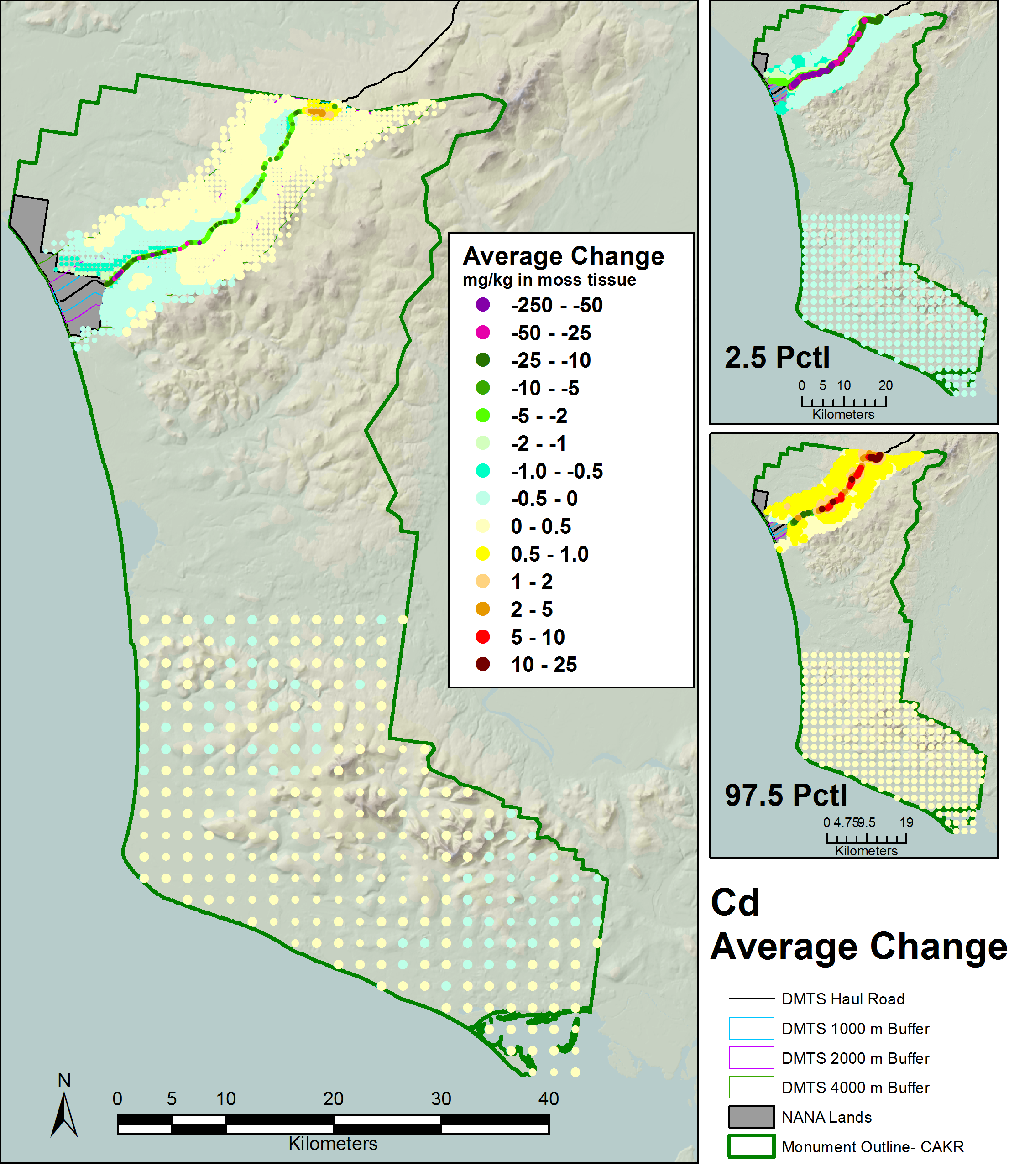

Supplement: S3 Fig — The 2.5th and 97.5th percentiles of the modeled concentrations are shown at right. Dots on the main graph are sized proportionally in four classes by the quartile distributions of the reciprocal of the CV. (TIF) [file pone.0177936.s003.tif]

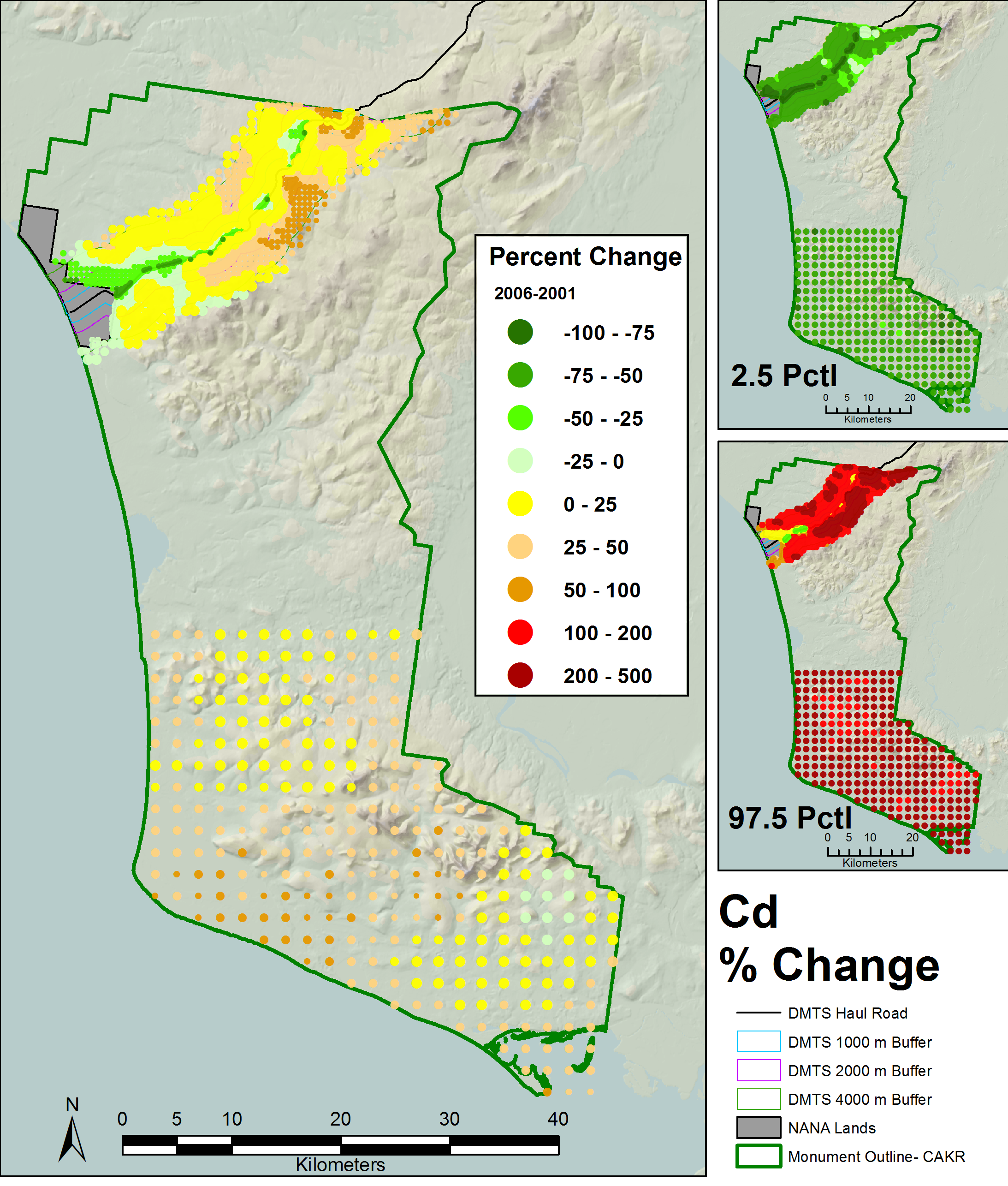

Supplement: S4 Fig — The 2.5th and 97.5th percentiles of the modeled concentrations are shown at right. Dots on the main graph are sized proportionally in four classes by the quartile distributions of the reciprocal of the CV. (TIF) [file pone.0177936.s004.tif]

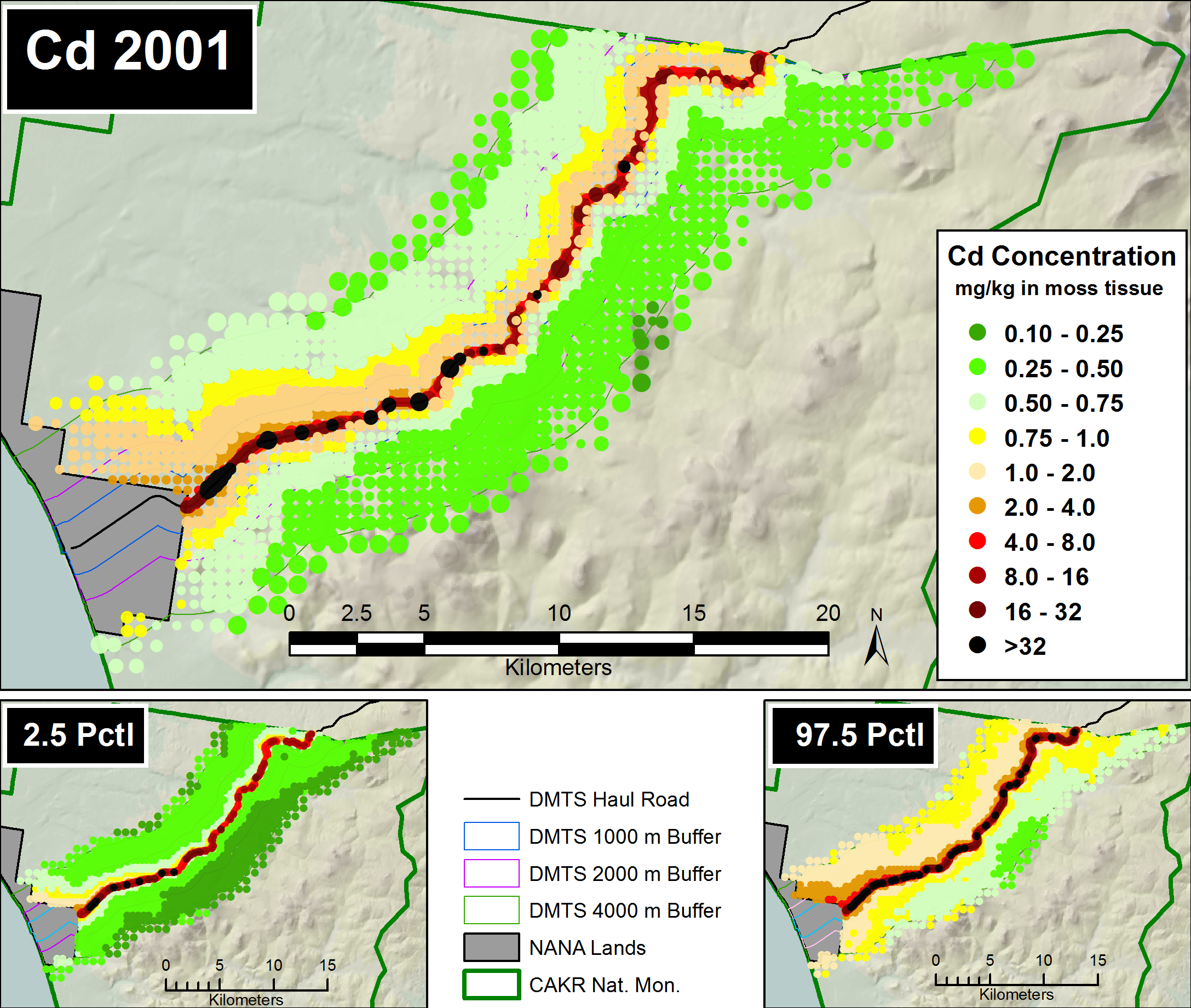

Supplement: S5 Fig — The 2.5th and 97.5th percentiles of the modeled concentrations are shown at right. Dots on the main graph are sized proportionally in four classes by the quartile distributions of the reciprocal of the CV. (TIF) [file pone.0177936.s005.tif]

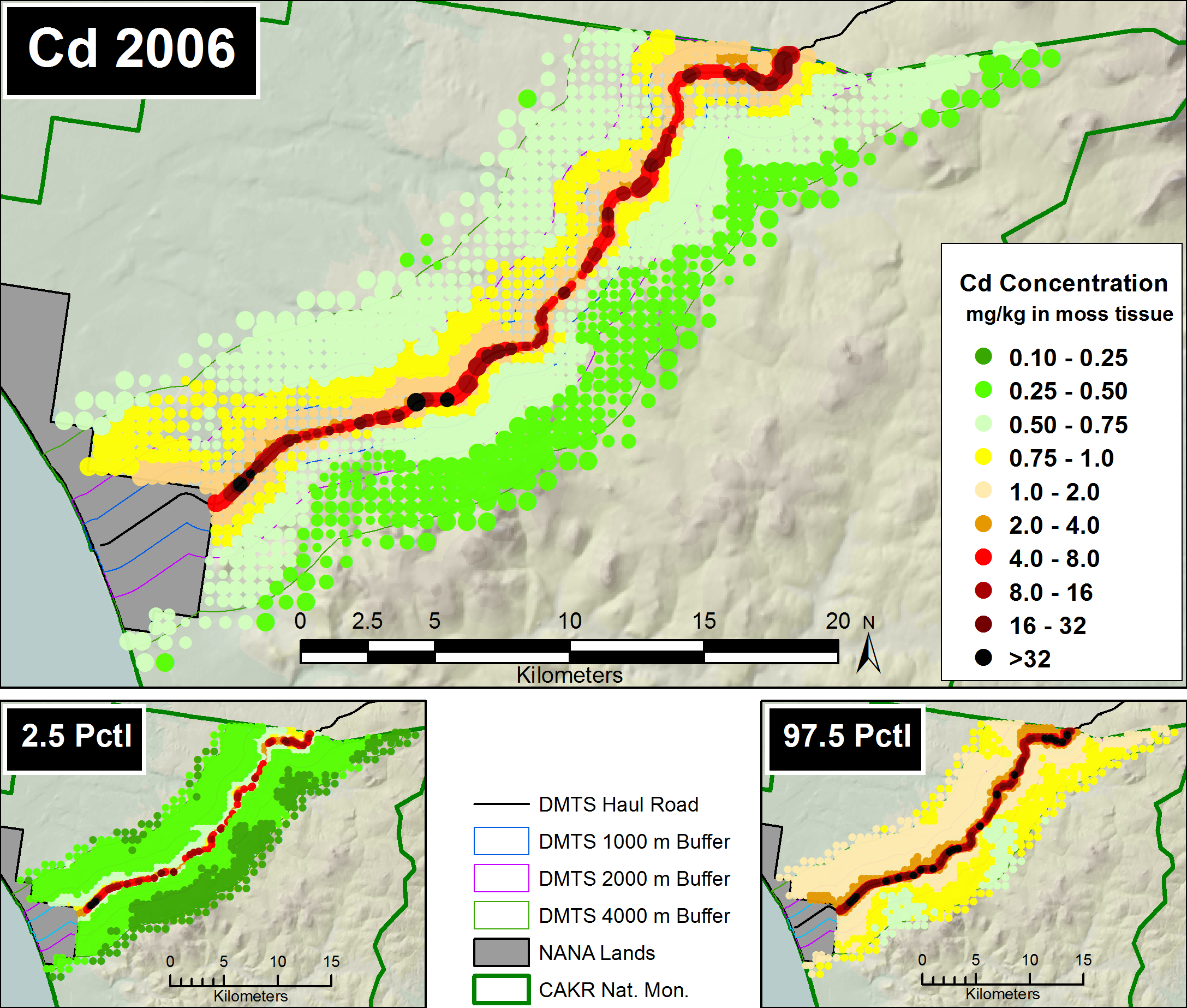

Supplement: S6 Fig — The 2.5th and 97.5th percentiles of the modeled concentrations are shown at right. Dots on the main graph are sized proportionally in four classes by the quartile distributions of the reciprocal of the CV. (TIF) [file pone.0177936.s006.tif]

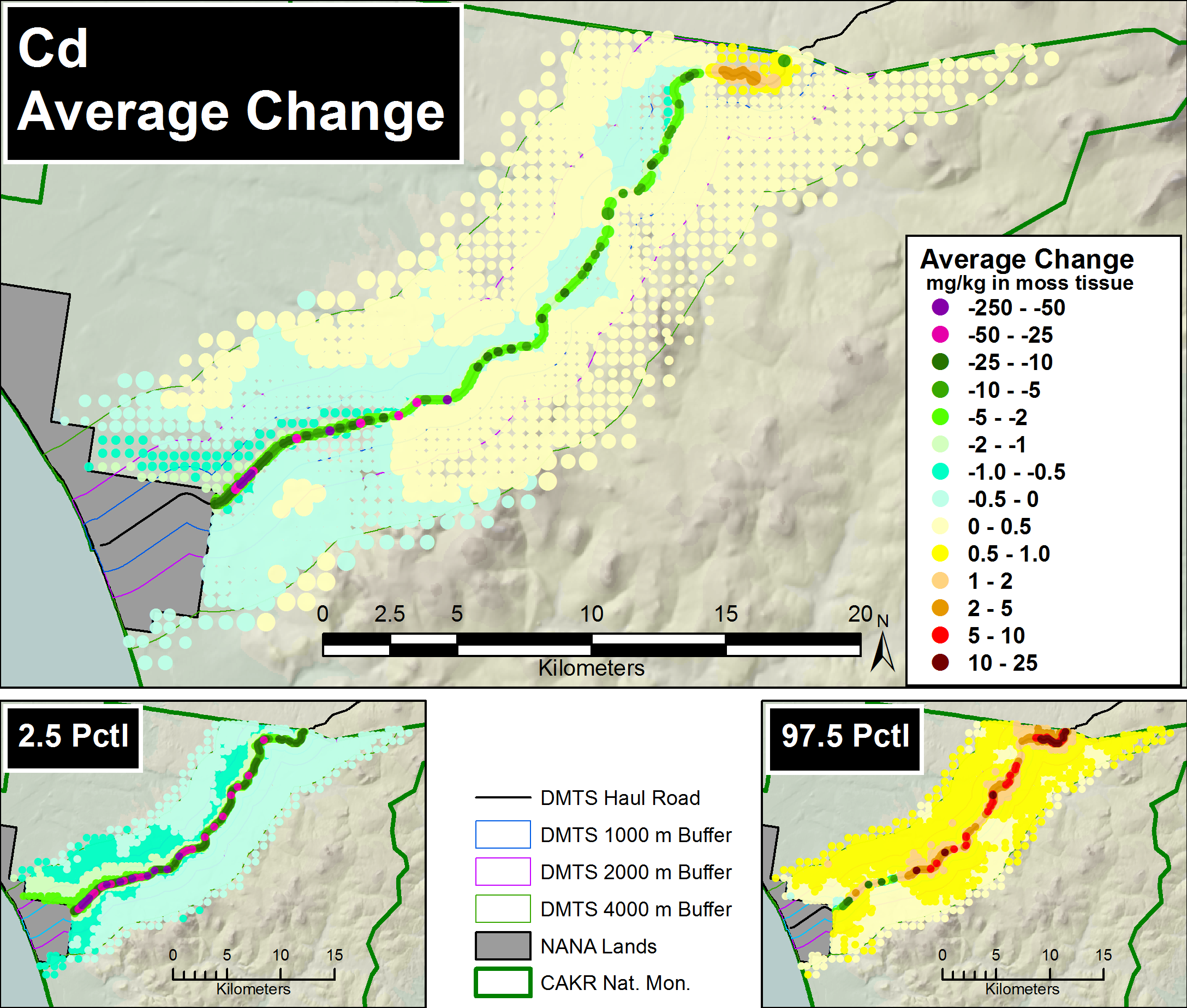

Supplement: S7 Fig — The 2.5th and 97.5th percentiles (lower and upper bound of the 95% interval) of the modeled concentrations are shown at right. Dots on the main graph are sized proportionally in four classes by the quartile distributions of the reciprocal of the CV. (TIF) [file pone.0177936.s007.tif]

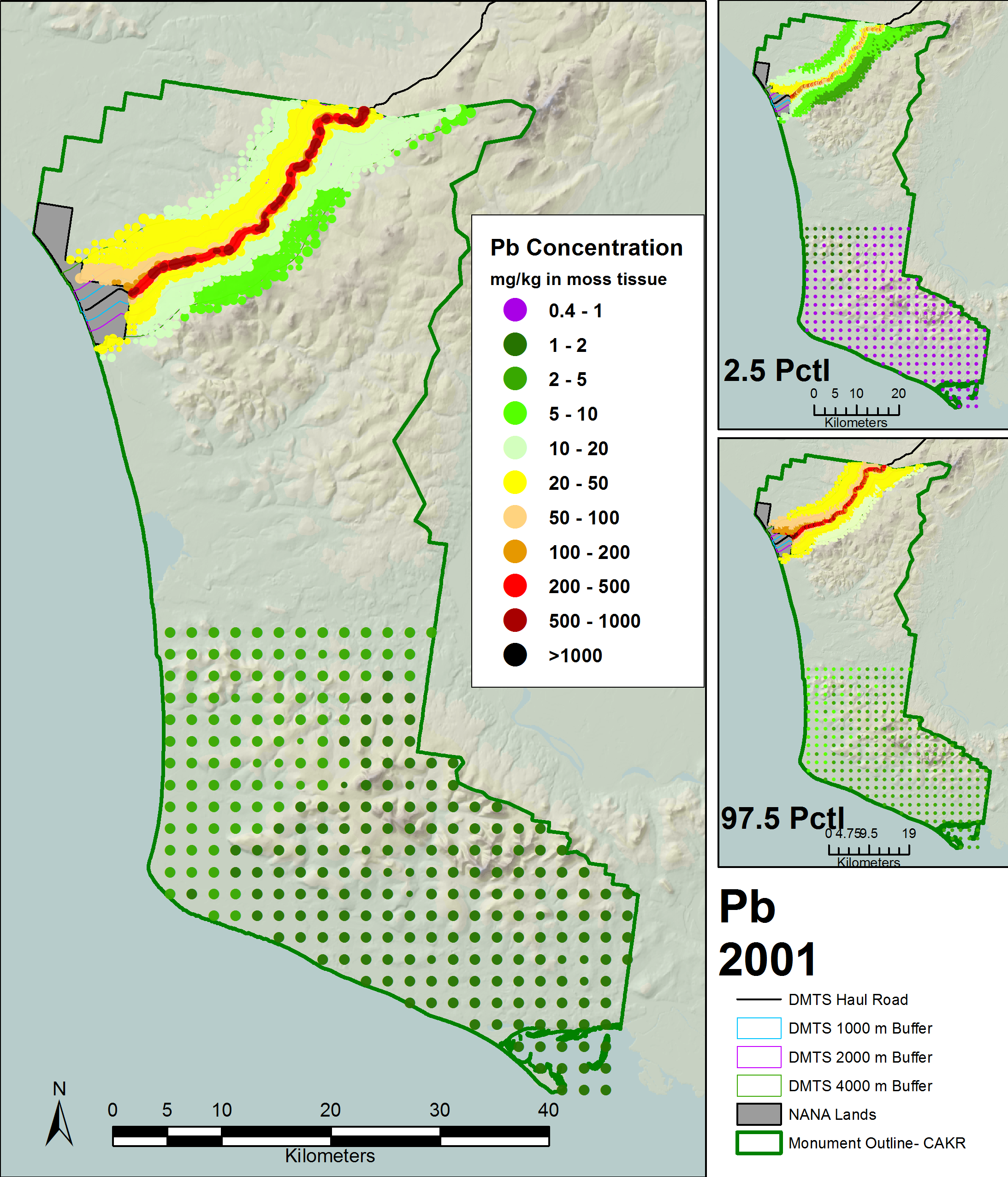

Supplement: S8 Fig — The 2.5th and 97.5th percentiles (lower and upper bound of the 95% interval) of the modeled concentrations are shown at right. Dots on the main graph are sized proportionally in four classes by the quartile distributions of the reciprocal of the CV. (TIF) [file pone.0177936.s008.tif]

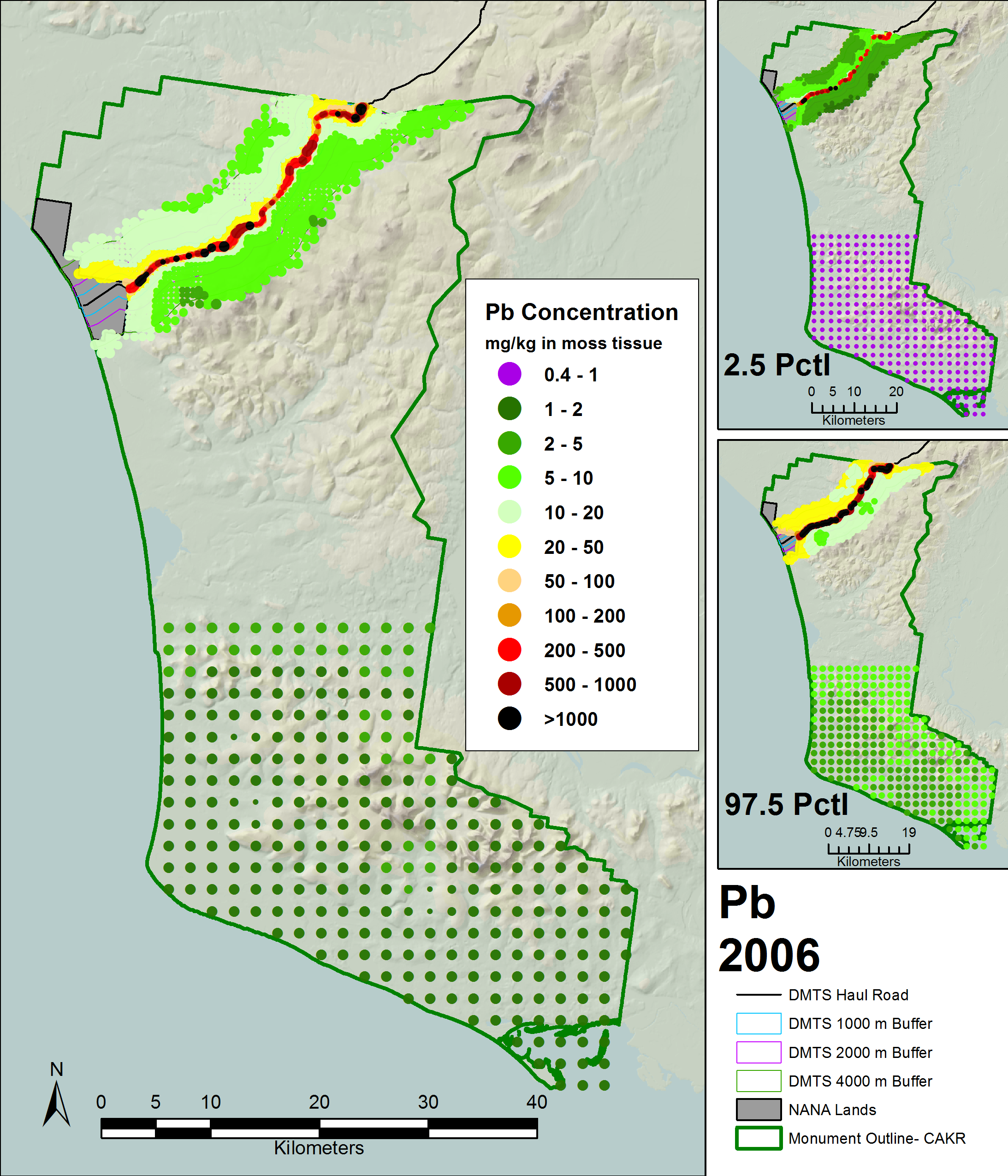

Supplement: S9 Fig — The 2.5th and 97.5th percentiles (lower and upper bound of the 95% interval) of the modeled concentrations are shown at right. Dots on the main graph are sized proportionally in four classes by the quartile distributions of the reciprocal of the CV. (TIF) [file pone.0177936.s009.tif]

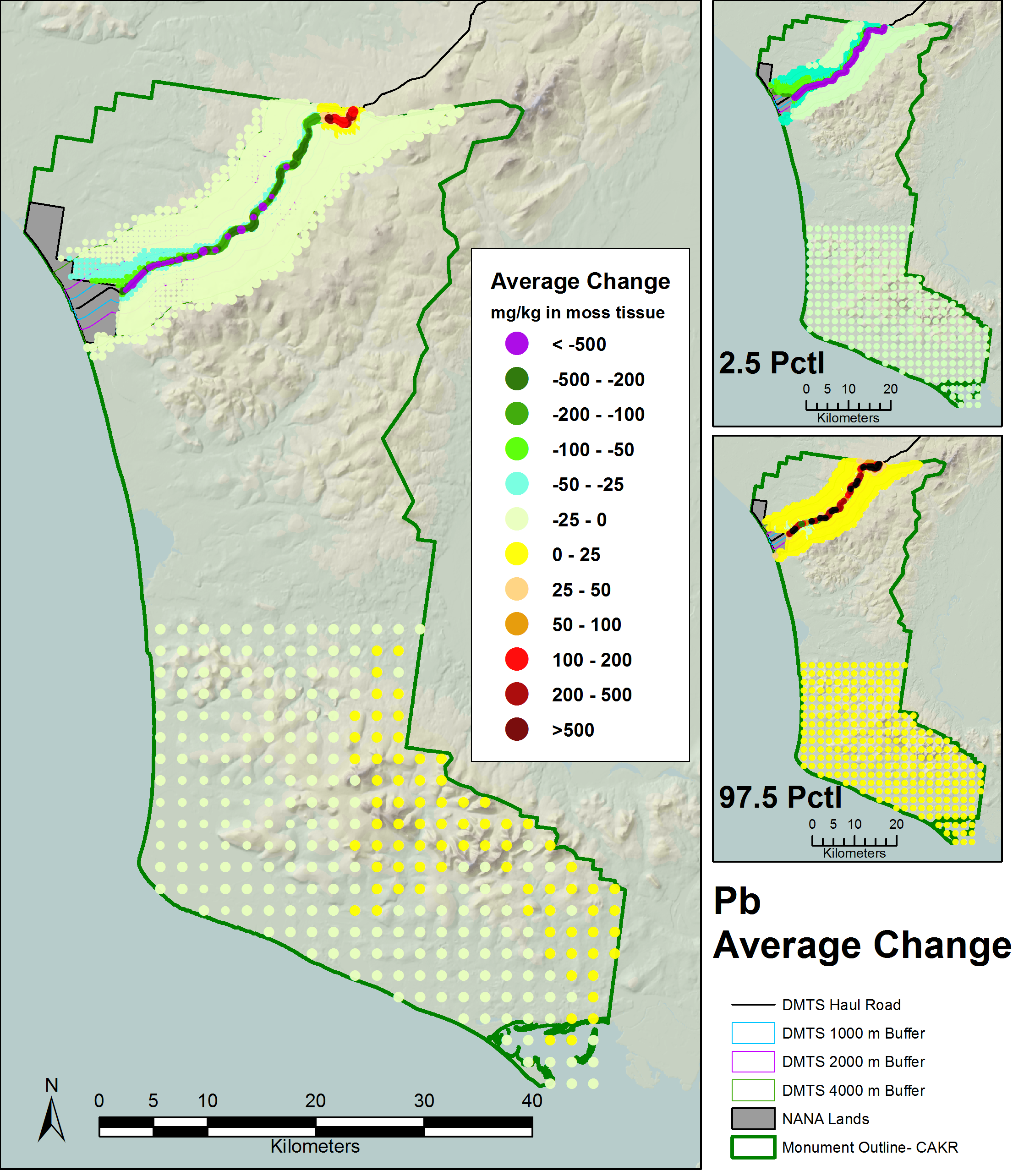

Supplement: S10 Fig — The 2.5th and 97.5th percentiles (lower and upper bound of the 95% interval) of the modeled concentrations are shown at right. Dots on the main graph are sized proportionally in four classes by the quartile distributions of the reciprocal of the CV. (TIF) [file pone.0177936.s010.tif]

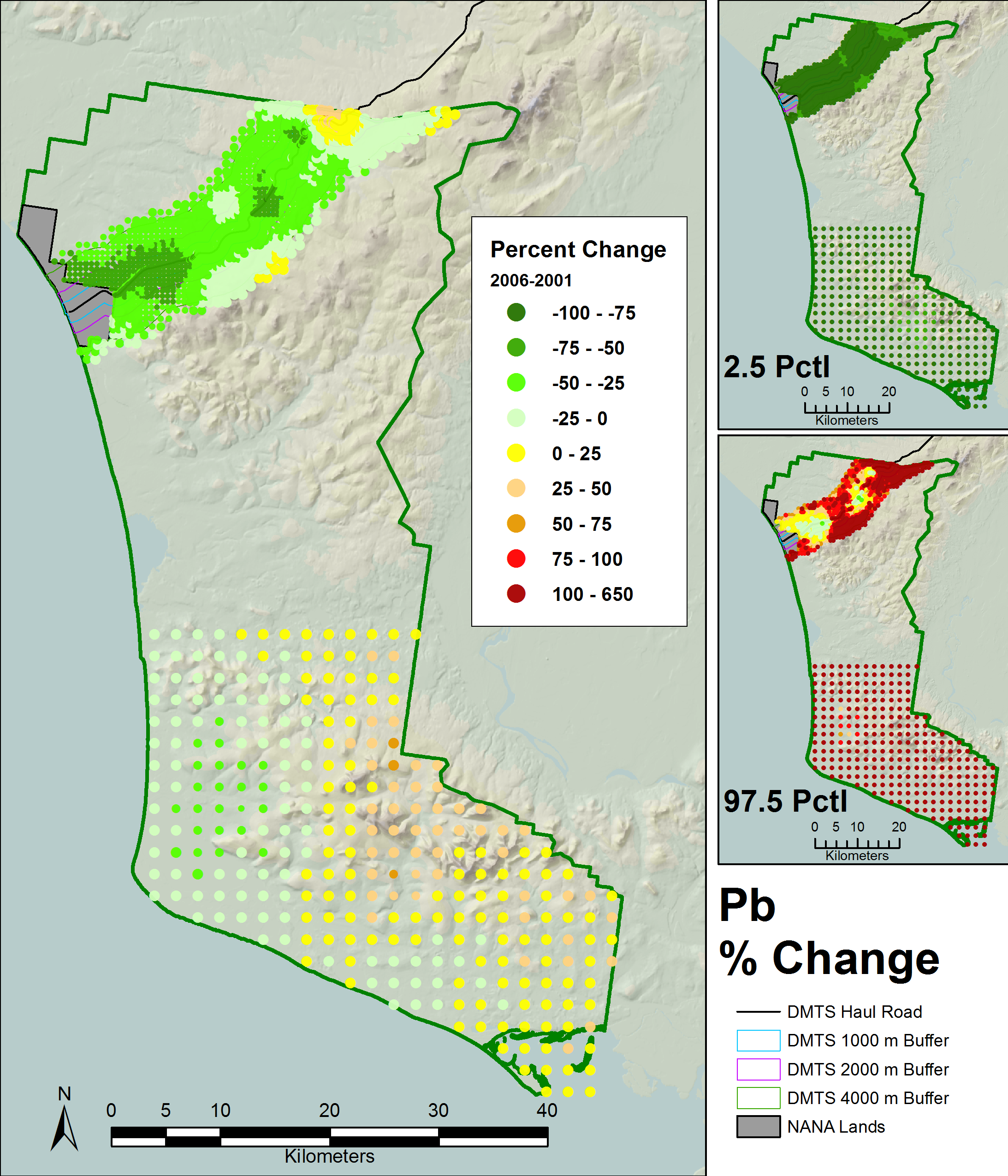

Supplement: S11 Fig — The 2.5th and 97.5th percentiles (lower and upper bound of the 95% interval) of the modeled concentrations are shown at right. Dots on the main graph are sized proportionally in four classes by the quartile distributions of the reciprocal of the CV. (TIF) [file pone.0177936.s011.tif]

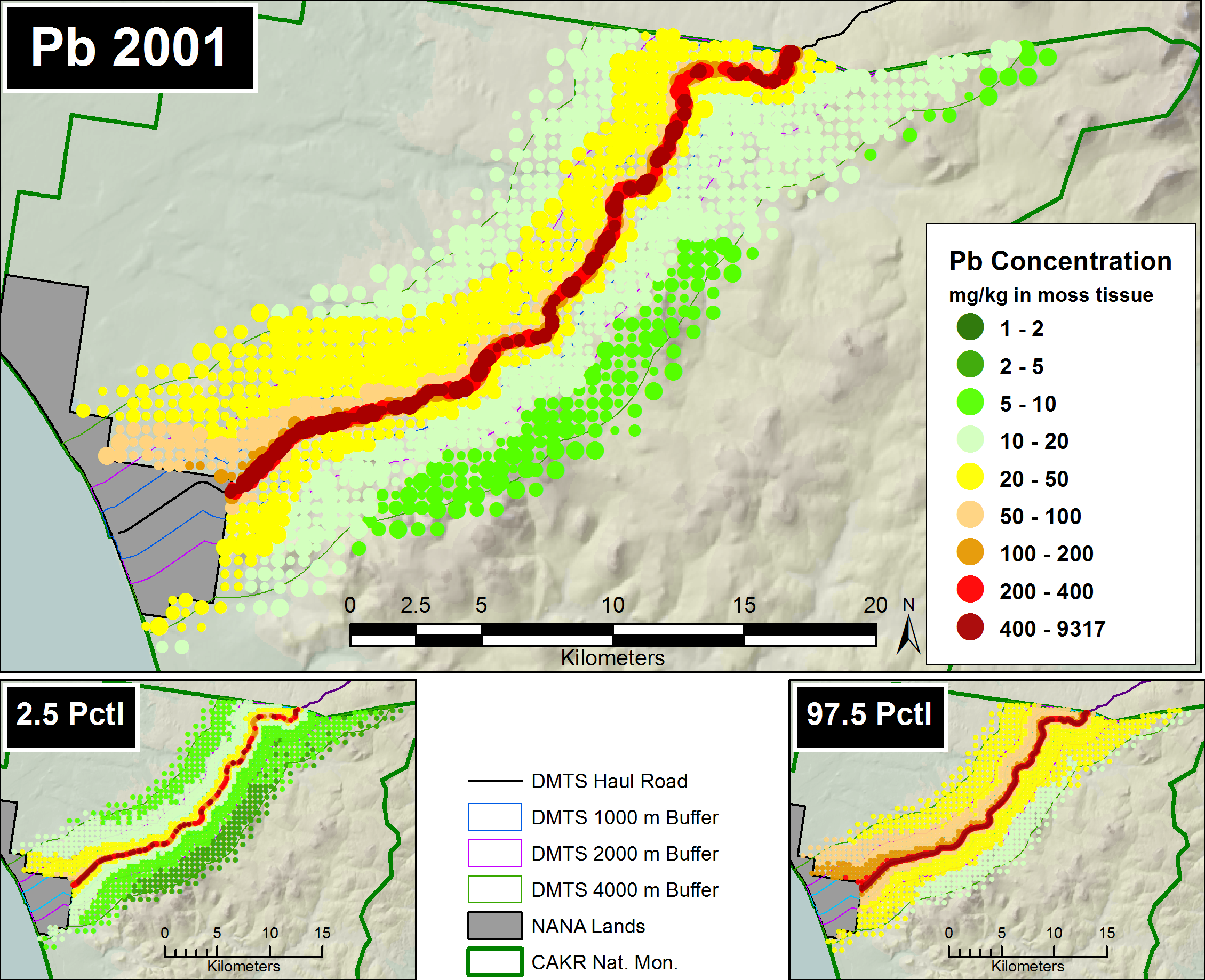

Supplement: S12 Fig — The 2.5th and 97.5th percentiles (lower and upper bound of the 95% interval) of the modeled concentrations are shown at right. Dots on the main graph are sized proportionally in four classes by the quartile distributions of the reciprocal of the CV. (TIF) [file pone.0177936.s012.tif]

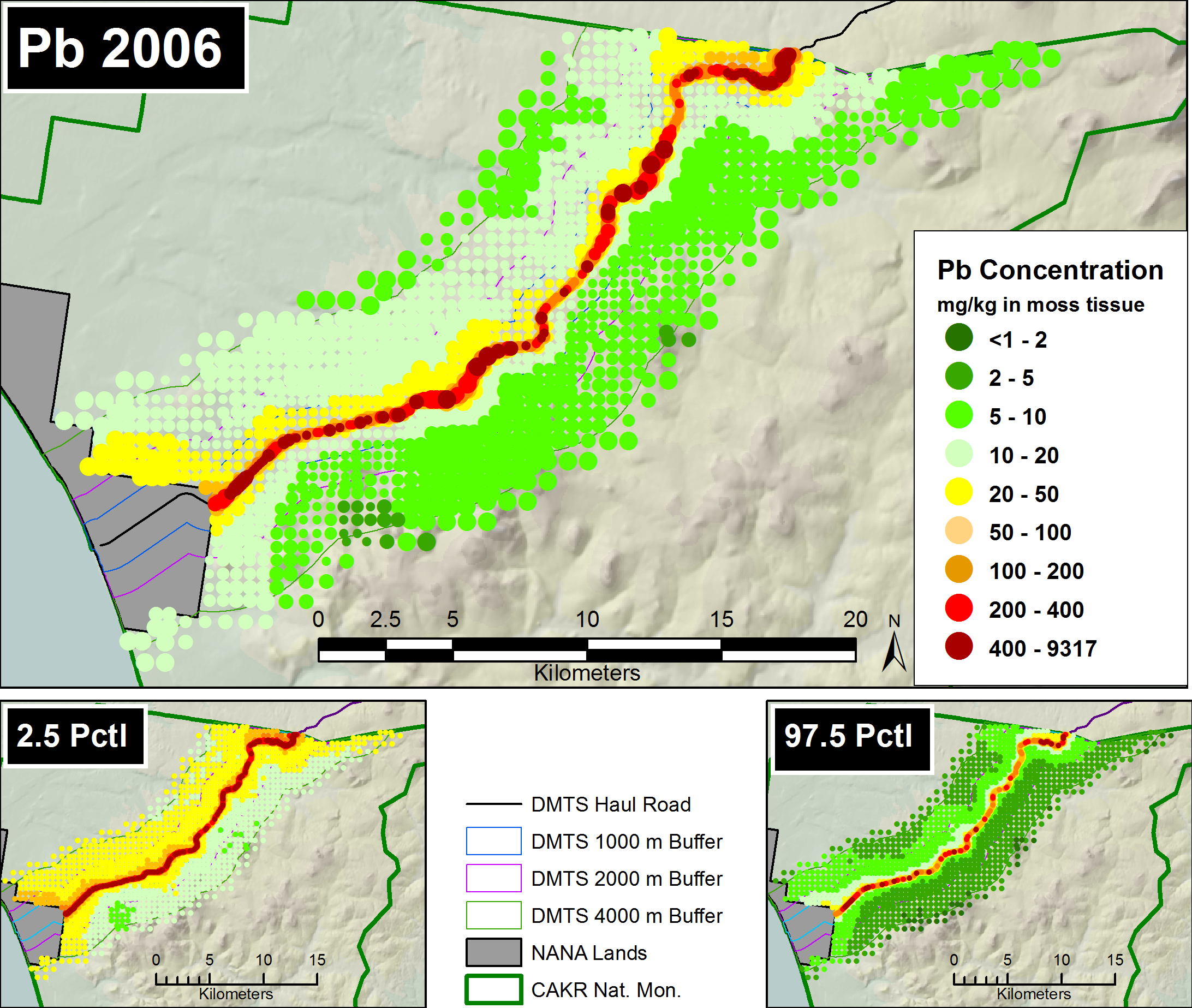

Supplement: S13 Fig — The 2.5th and 97.5th percentiles (lower and upper bound of the 95% interval) of the modeled concentrations are shown at right. Dots on the main graph are sized proportionally in four classes by the quartile distributions of the reciprocal of the CV. (TIF) [file pone.0177936.s013.tif]

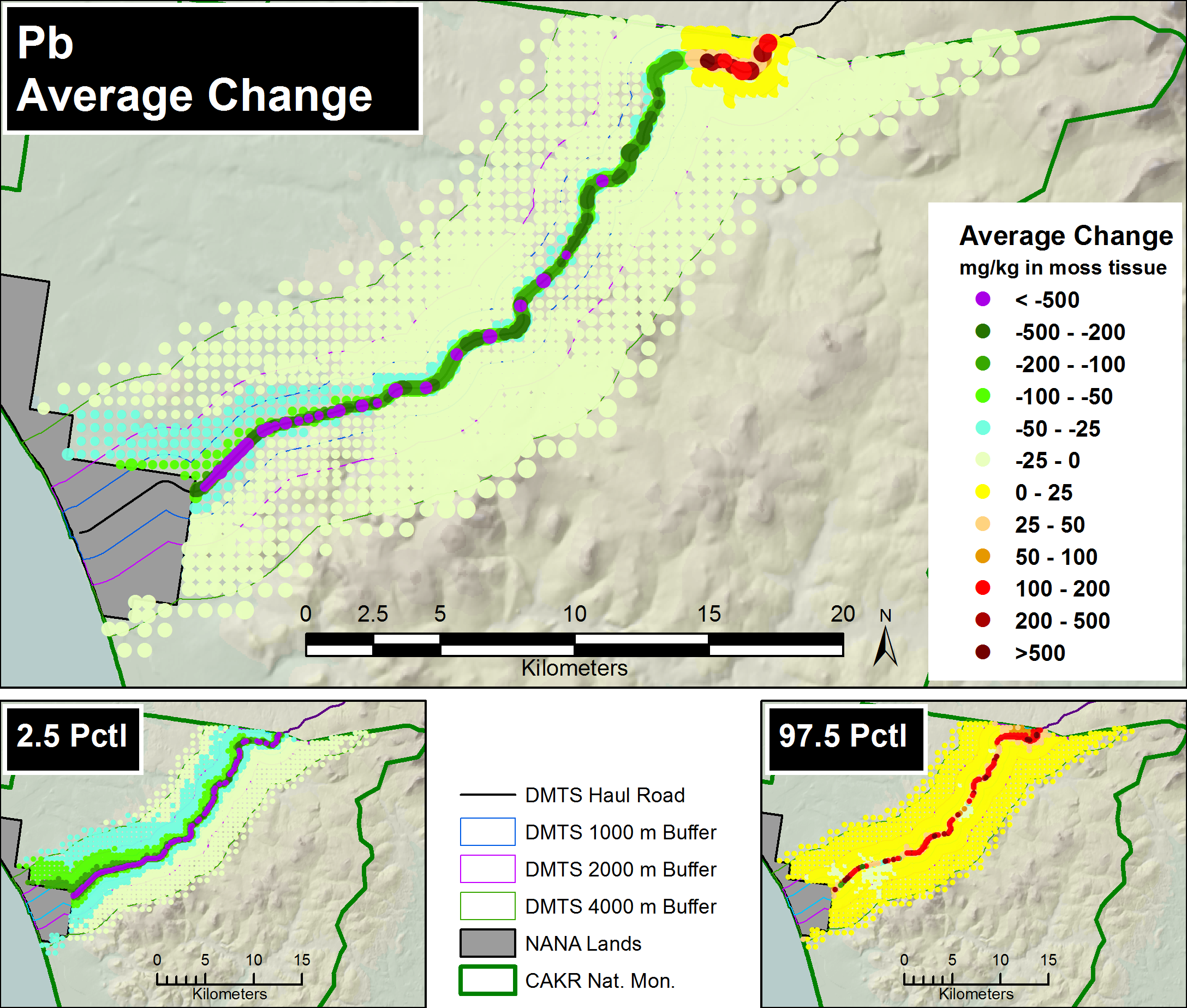

Supplement: S14 Fig — The 2.5th and 97.5th percentiles (lower and upper bound of the 95% interval) of the modeled concentrations are shown at right. Dots on the main graph are sized proportionally in four classes by the quartile distributions of the reciprocal of the CV. (TIF) [file pone.0177936.s014.tif]

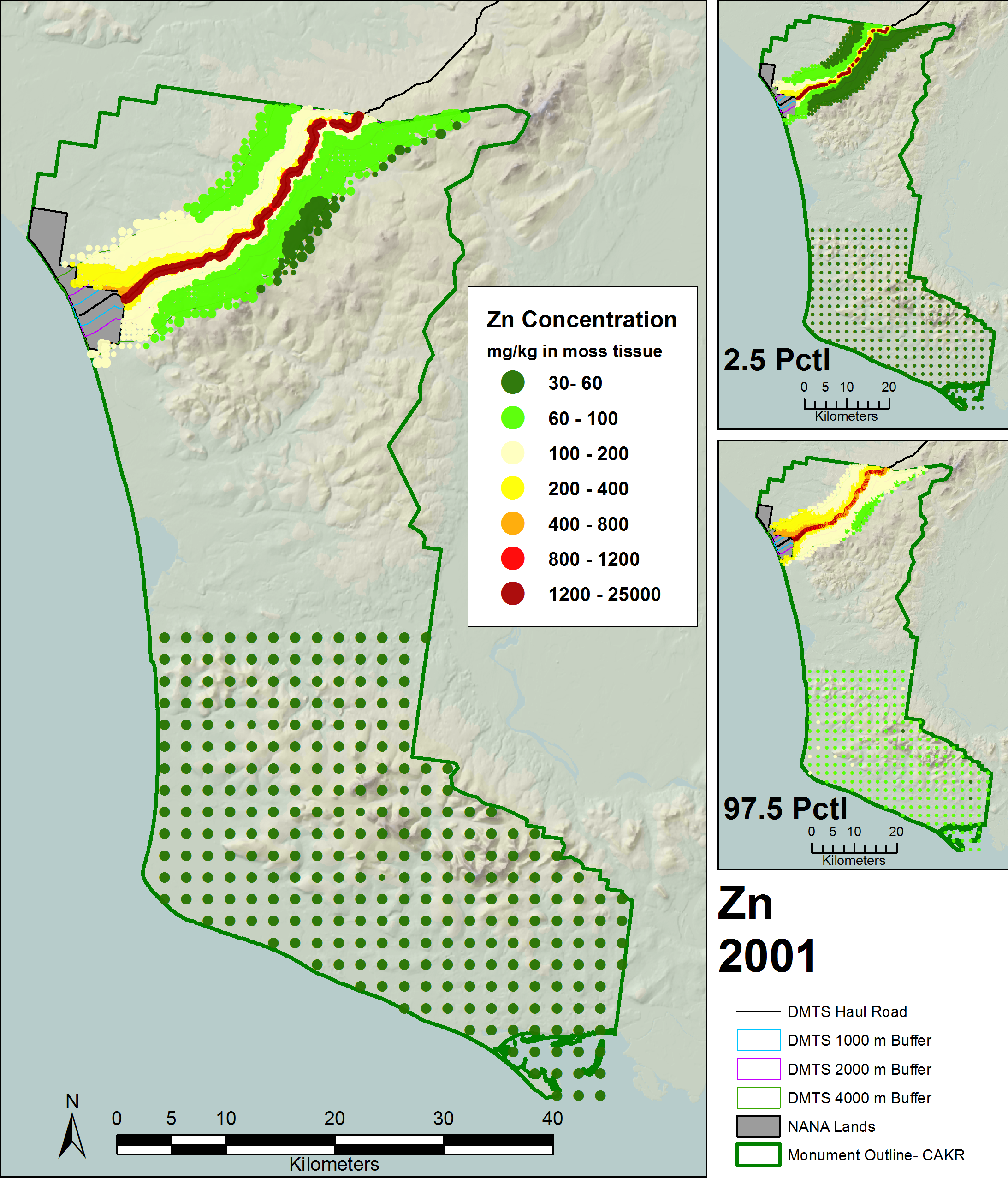

Supplement: S15 Fig — The 2.5th and 97.5th percentiles (lower and upper bound of the 95% interval) of the modeled concentrations are shown at right. Dots on the main graph are sized proportionally in four classes by the quartile distributions of the reciprocal of the CV. (TIF) [file pone.0177936.s015.tif]

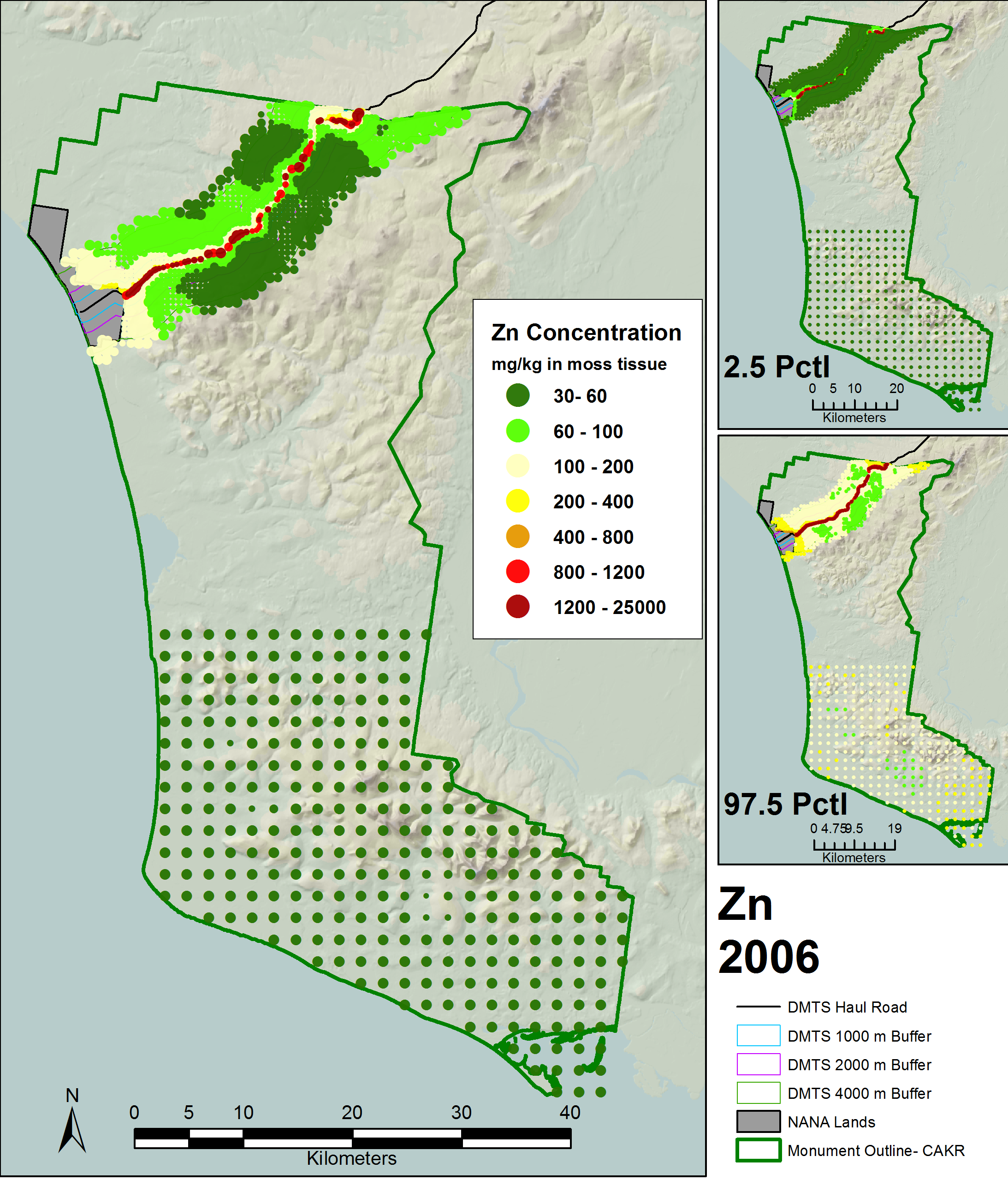

Supplement: S16 Fig — The 2.5th and 97.5th percentiles (lower and upper bound of the 95% interval) of the modeled concentrations are shown at right. Dots on the main graph are sized proportionally in four classes by the quartile distributions of the reciprocal of the CV. (TIF) [file pone.0177936.s016.tif]

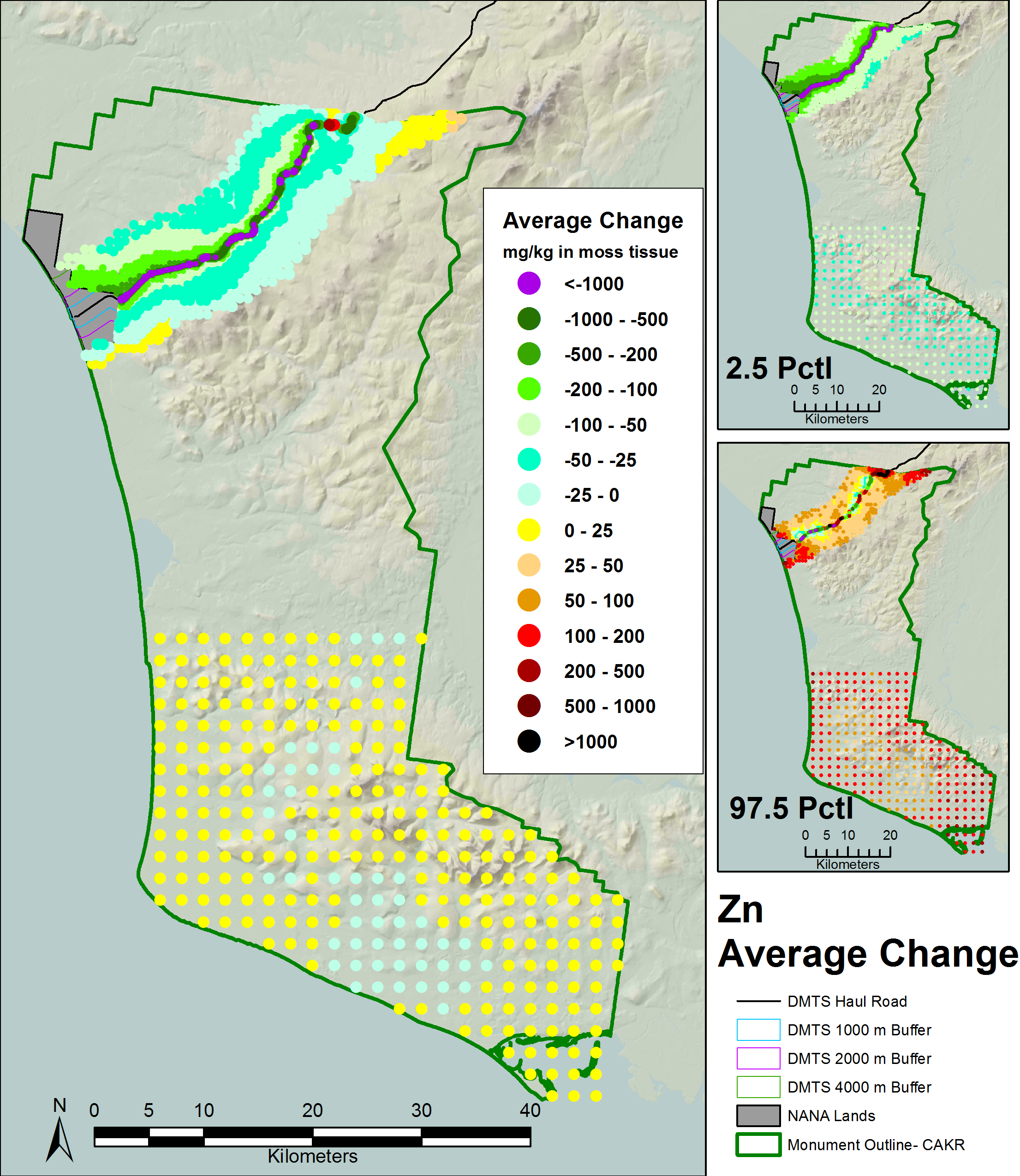

Supplement: S17 Fig — The 2.5th and 97.5th percentiles (lower and upper bound of the 95% interval) of the modeled concentrations are shown at right. Dots on the main graph are sized proportionally in four classes by the quartile distributions of the reciprocal of the CV. (TIF) [file pone.0177936.s017.tif]

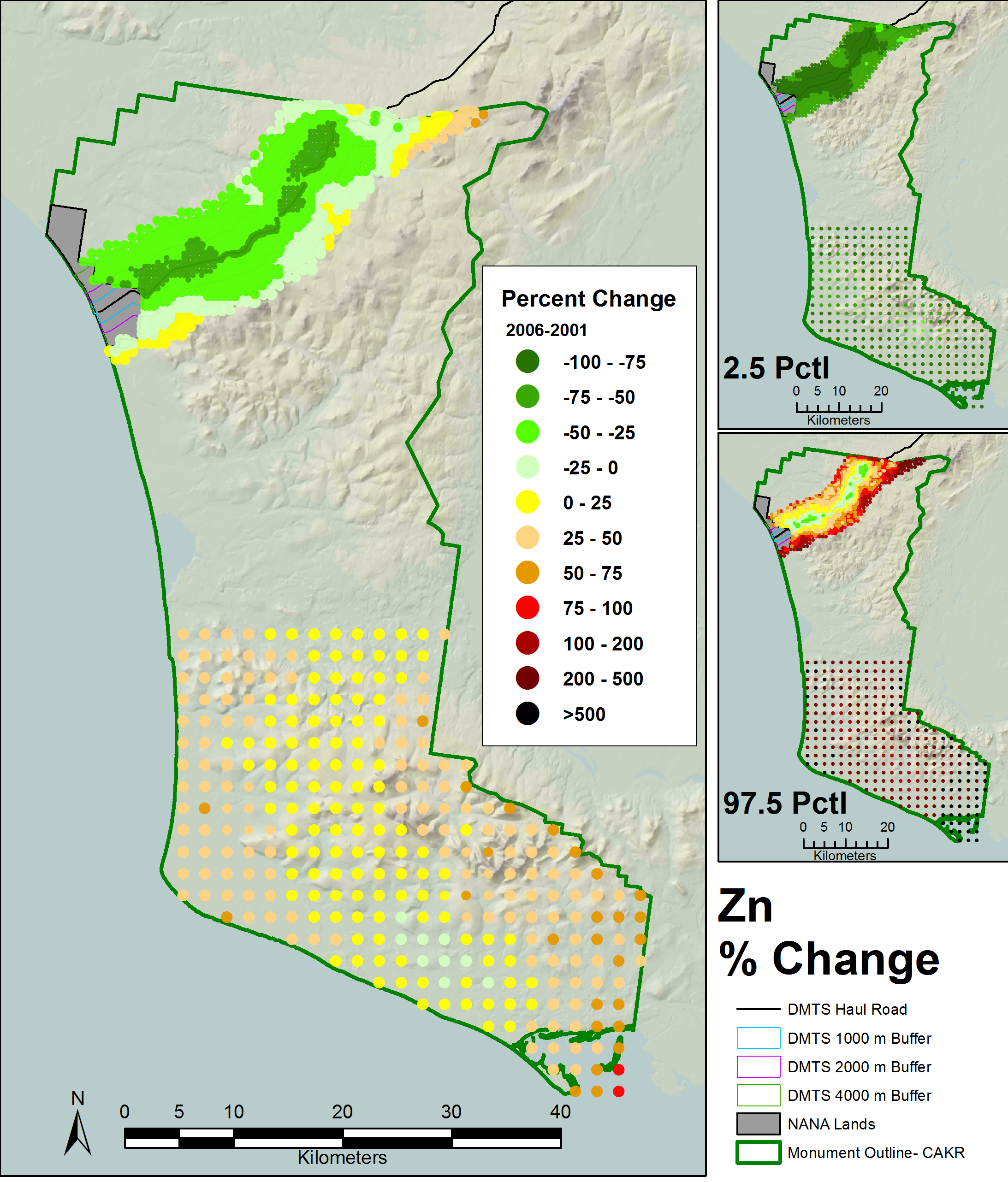

Supplement: S18 Fig — The 2.5th and 97.5th percentiles (lower and upper bound of the 95% interval) of the modeled concentrations are shown at right. Dots on the main graph are sized proportionally in four classes by the quartile distributions of the reciprocal of the CV. (TIF) [file pone.0177936.s018.tif]

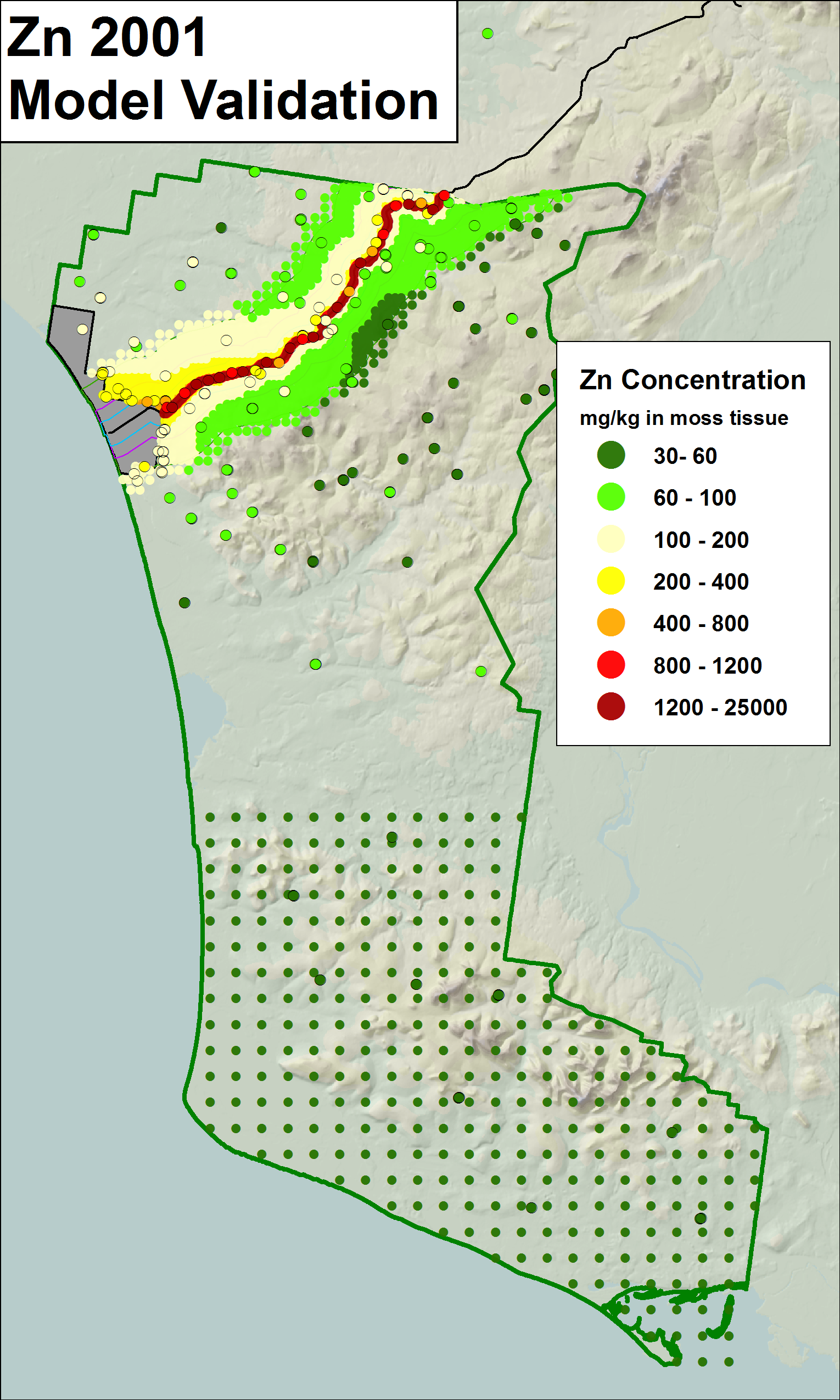

Supplement: S19 Fig — (TIF) [file pone.0177936.s019.tif]

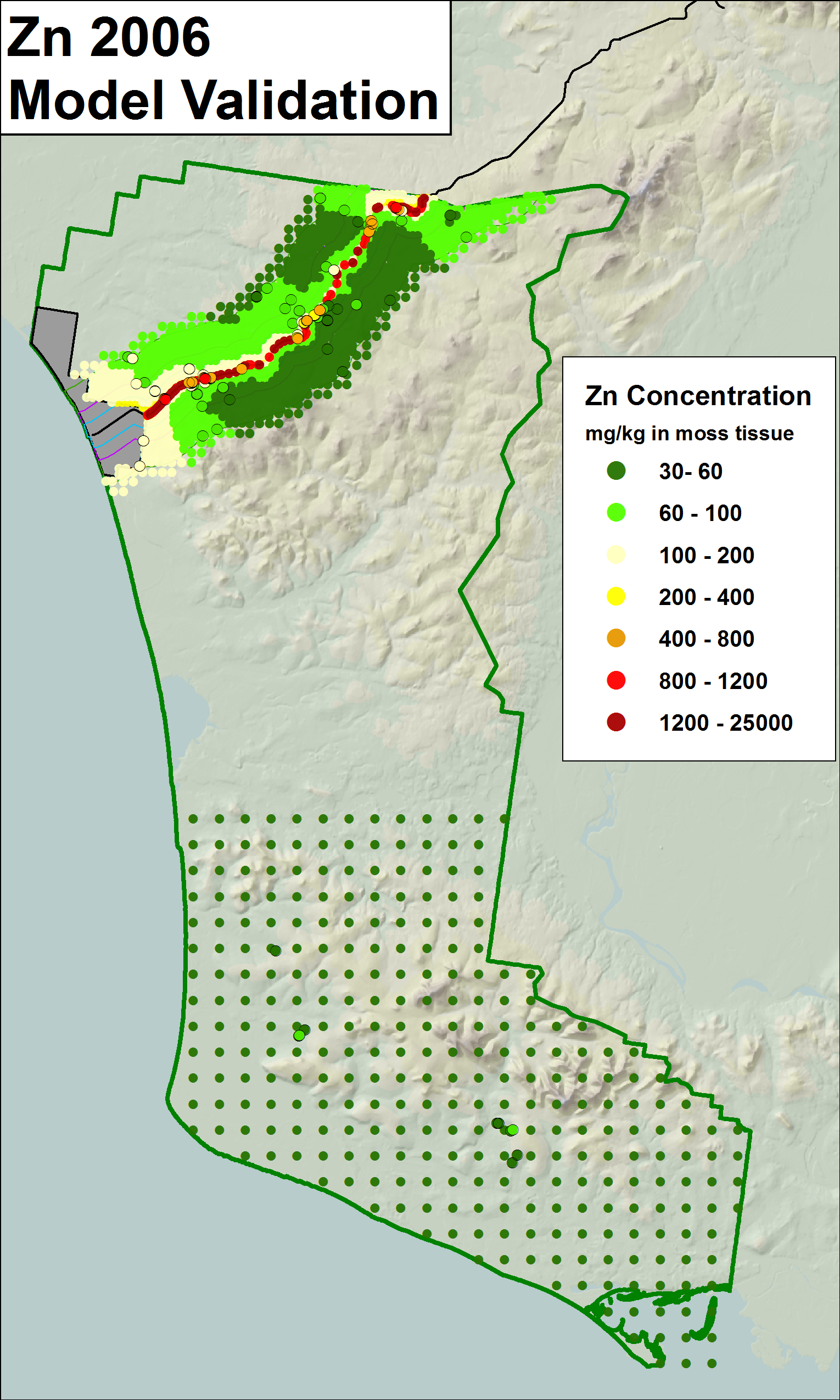

Supplement: S20 Fig — (TIF) [file pone.0177936.s020.tif]

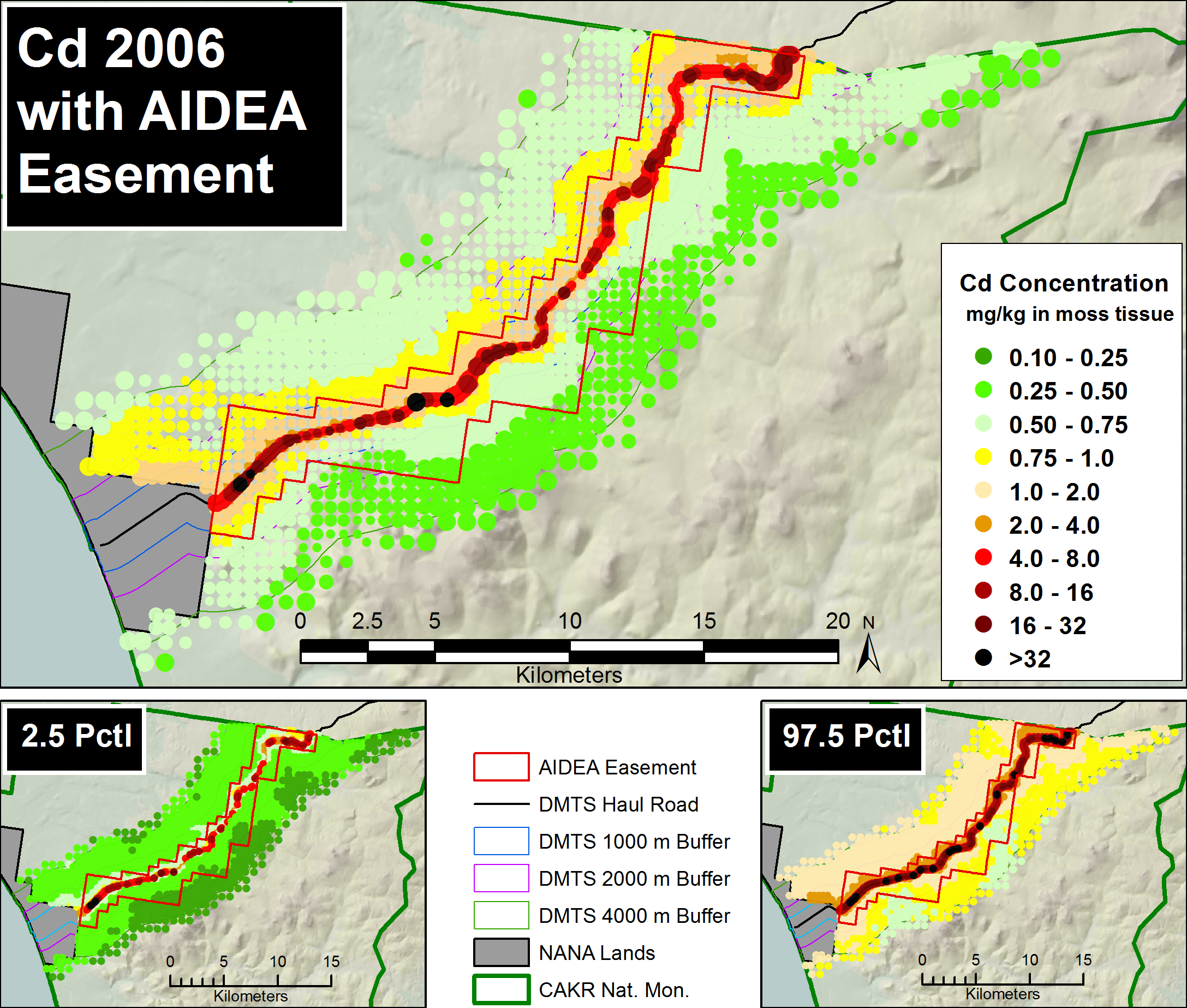

Supplement: S21 Fig — The 2.5th and 97.5th percentiles of the modeled concentrations are shown at right. Dots on the main graph are sized proportionally in four classes by the quartile distributions of the reciprocal of the CV. The DMTS easement is plotted on top of Cd concentrations in moss. (TIF) [file pone.0177936.s021.tif]

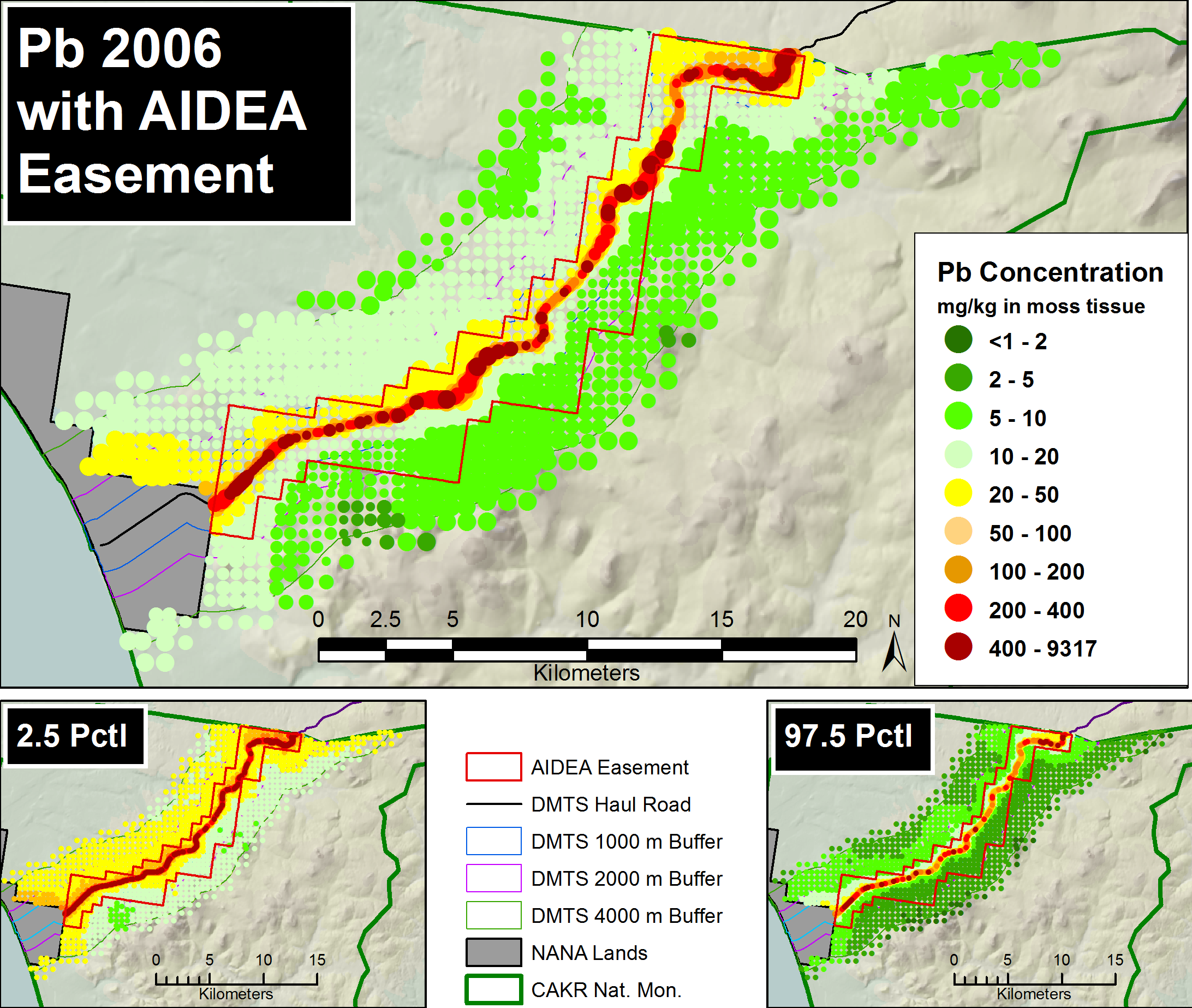

Supplement: S22 Fig — The 2.5th and 97.5th percentiles of the modeled concentrations are shown at right. Dots on the main graph are sized proportionally in four classes by the quartile distributions of the reciprocal of the CV. The DMTS easement is plotted on top of Pb concentrations in moss. (TIF) [file pone.0177936.s022.tif]

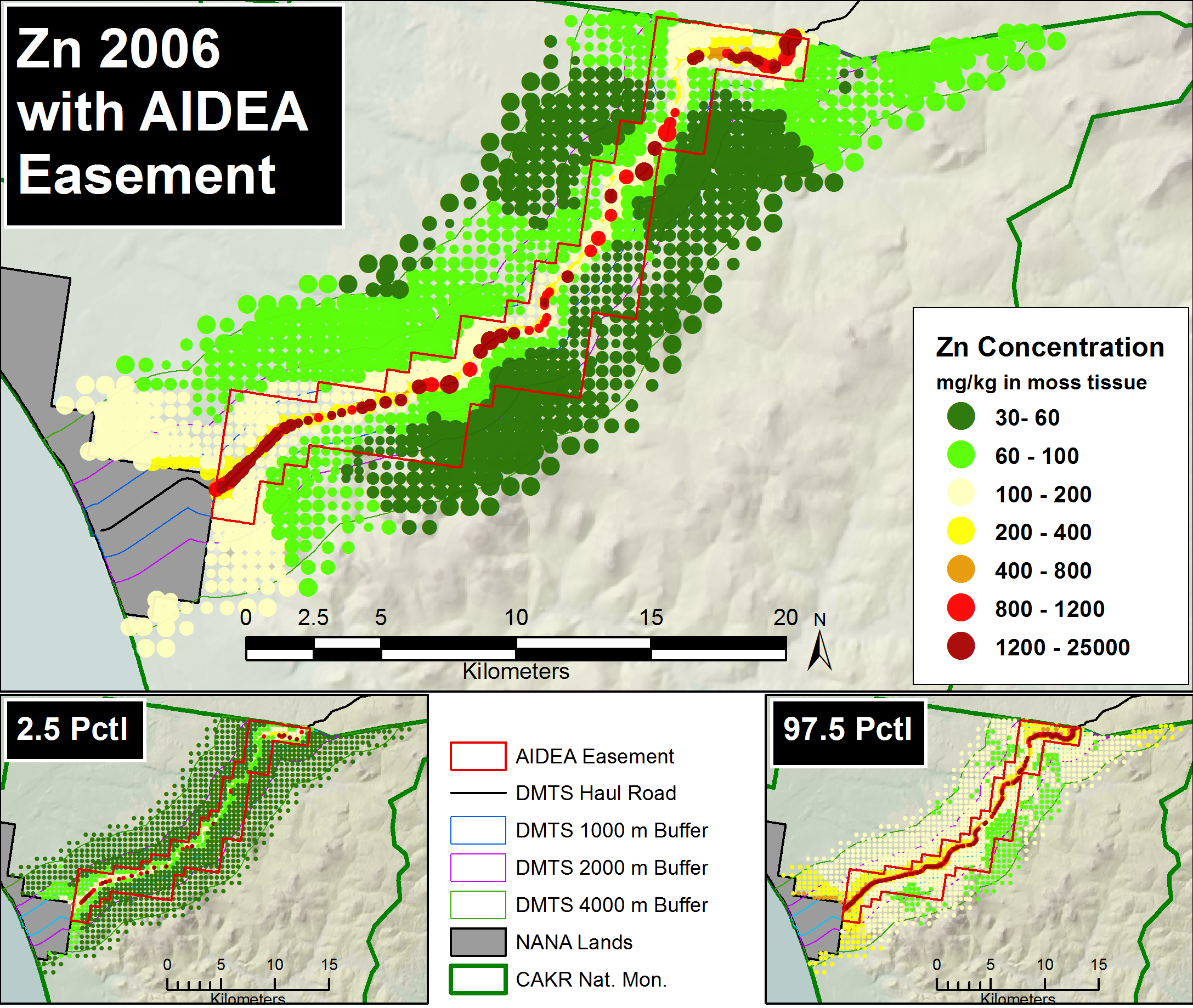

Supplement: S23 Fig — The 2.5th and 97.5th percentiles of the modeled concentrations are shown at right. Dots on the main graph are sized proportionally in four classes by the quartile distributions of the reciprocal of the CV. The DMTS easement is plotted on top of Zn concentrations in moss. (TIF) [file pone.0177936.s023.tif]

| Year | Total       |         |           |
|------|-------------|---------|-----------|
|      | Concentrate | Lead    | Zinc      |
| 2001 | 1,200,552   | 170,512 | 1,030,040 |
| 2002 | 1,368,436   | 210,047 | 1,158,389 |
| 2003 | 1,361,375   | 239,148 | 1,122,227 |
| 2004 | 1,366,293   | 236,273 | 1,130,020 |
| 2005 | 1,332,720   | 218,097 | 1,114,623 |
| 2006 | 1,376,804   | 249,706 | 1,127,098 |

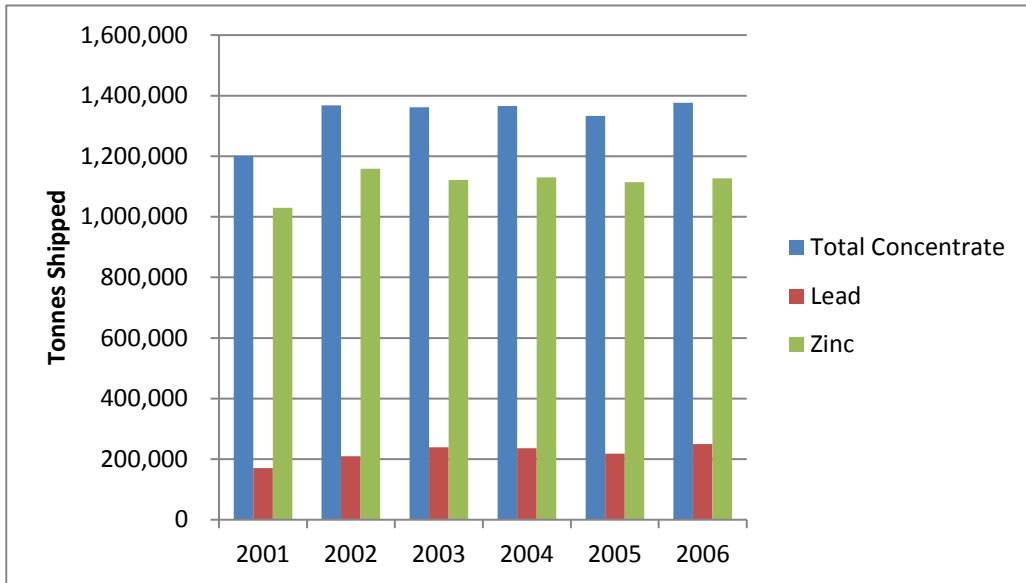

Supplement: S2 File — Provided by Teck, Inc., March 31, 2017. (PDF) [file pone.0177936.s025.pdf]
