# Supplementary material for: Trends in spatial patterns of heavy metal deposition on national park service lands along the Red Dog Mine haul road, Alaska, 2001–2006
Source: PLoS One. 2017 May 18;12(5):e0177936. doi: 10.1371/journal.pone.0177936 (PMC5436859; doi:10.1371/journal.pone.0177936)
Supplement: S1 File — (PDF) [file pone.0177936.s024.pdf]

S1 File. Laboratory Analysis Methods Used for 2006 Moss Tissue Samples.

Drying and Grinding

Air-dried samples were first passed through a stainless steel grinder with a 20-mesh sieve and mixed thoroughly. Ground samples were dried at 65°C for two (2) hours and cooled in a desiccator before weighing for analysis.

Total Sulfur

Total Sulfur was determined by combusting 150 mg of sample in a ceramic boat after mixing with Com-Cat Accelerator (LECO trade name for tungsten oxide compound), in an oxygen atmosphere at 1290° C in a LECO Sulfur Determinator, Model No. S144-DR. The SO_2 evolved from the sample was determined by a nondispersive infrared detector empirically calibrated with LECO plant reference materials (LECO Corporation, 3000 Lakeview Dr., St. Joseph, MI 49085).

Total Nitrogen

Total nitrogen was determined by combustion. Total Kjeldahl nitrogen was determined by converting the various forms of nitrogen to NH4+, measuring NH4+ concentration, and subtracting the weight of hydrogen. To accomplish this, 0.150 g of dry, ground plant material was digested in 3.5 ml concentrated H_2SO_4 with 1.5 mg K_2SO_4 and 7.5 mg selenium. This mixture was placed in an electrically heated aluminum block at 400°C and digested for 1 hour. The NH_4+ formed was reacted with salicylate in the presence of hypochlorite and nitro-prusside to form an emerald-green complex. Color intensity was measured spectrophotometrically on a Technicon AutoAnalyzer at 660 nm. The method converted only partial amounts of nitrate, thus samples containing high concentrations of nitrates were pretreated with salicylic acid to ensure complete conversion.

The combustion method for total nitrogen used a LECO FP-528 Nitrogen Analyzer. A 150-500 mg sample was weighed into a gel capsule and dropped into an 850° C furnace purged with O_2 gas. The combustion products (CO_2, H_2O and NO_x) were filtered, cooled by a thermoelectric cooler to condense most of the water, and collected into large ballast. A 3 cc aliquot of the ballast combustion product was integrated into a helium carrier stream. The stream first passed through a hot copper column to remove O_2 and convert NO_x to N_2. A reagent tube then scrubbed the remaining CO_2 and H_2O from the stream. N_2 content was measured by a thermal conductivity cell against a helium background and the result was displayed as a weight percentage of nitrogen.

Metals

The elements P, K, Ca, Mg, Na, Al, Fe, Mn, Zn, Cu, B, Pb, Ni, Cr, Cd were determined using a Perken-Elmer model Optima 3000 DV simultaneous inductively coupled-atomic emission spectrometer (ICP-AES, [S1]). For this analysis, a 0.25-0.50 g sample of dried plant material

was digested with 2 mL H_2O_2 and 0.5 mL trace metal grade HNO_3 in a microwave digestion vessel for 4 min at 296 watts and 8 min at 565 watts.

Each sample was rerun using co-axial viewing for Pb, Cr and Cd to increase detection limits. Co-axial values were substituted for normal ICP analysis values when the normal values fell at or under detection limits.

Laboratory QA/QC

Calibration checks, blanks, National Institute of Standards and Technology (NIST) standard reference materials, and International Atomic Energy Agency (IAEA) reference check samples were analyzed to check accuracy and precision of moss results. At least one QA sample was run after every 10 unknowns. IAEA-336 (the lichen *Evernia prunastri*) reference material [S2] was used for outside reference check samples. The mean percent difference between UMN's analysis of IAEA-336 and either the recommended or information values for Cd, Cr, Cu, Fe, K, Mn, Na, P, Pb, Zn was -15.6% in two checks. Al was excluded from this analysis as it had a large outlier value of -60%. The mean percent difference between lab samples and lab duplicates or field duplicates for Zn, Cd and Pb was 1.0%. The mean percent difference of the absolute values of the differences between samples (i.e., the mean differences without regard to positive or negative deviation) and their duplicates for Zn, Cd, and Pb was 11.7%.

References

S1. Dahlquist, R. L., and J. W.Knoll. 1978. Inductively coupled plasma-atomic emission spectrometry: Analyses of biological materials and soils for trace and ultra-trace elements. Applied Spectroscopy 32:1–30.

S2. Heller-Zeisler, S. F., R. Zeisler, E. Zeiller, R. M. Parr, Z. Radecki, K. I. Burns, P. De Regge. 1999. Report on the intercomparison run for the determination of trace and minor elements in lichen material IAEA-336. Section for Nutritional and Health-related Environmental Studies, Division of Human Health and Analytical Quality Control Services, Agency's Laboratories, Seibersdorf International Atomic Energy Agency P.O. Box 100 A-1400 Vienna, Austria June 1999. http://www.iaea.org/programmes/aqcs/pdf/al_079.pdf
