# Supplementary material for: Trends in spatial patterns of heavy metal deposition on national park service lands along the Red Dog Mine haul road, Alaska, 2001–2006
Source: PLoS One. 2017 May 18;12(5):e0177936. doi: 10.1371/journal.pone.0177936 (PMC5436859; doi:10.1371/journal.pone.0177936)
Supplement: S1 Table — % N>S represents the percentage by which the north side exceeds the south side for a given metric. Strata for which the 95% credible interval in average change spanned zero for the north or south side have higher uncertainty are shown in blue with a smaller font. (PDF) [file pone.0177936.s026.pdf]

		2001 Concentration				2006 Concentration			% Change 2001-2006		
**Element**	**Stratum**	**N**	**S**	**% N>S**		**N**	**S**	**% N>S**	**N**	**S**	**% N>S**
Cd	1	9.96	6.94	**44**		6.43	4.05	**59**	-35	-41	**-15**
Cd	2	1.43	0.75	**91**		1.19	0.8	**49**	-16	6.4	**-350**
Cd	3	0.7	0.42	**67**		0.68	0.48	**42**	-2.6	14	**-119**
Cd	4	0.56	0.4	**40**		0.58	0.43	**35**	4.3	10	**-57**
Cd	5	-	0.14	**-**	-	-	0.15	-	-	5.5	-
Pb	1	411	321	**28**		249	148	**68**	-39	-52	**-25**
Pb	2	53	25	**112**		28	14	**100**	-47	-42	**12**
Pb	3	26	12	**117**		13	7.6	**71**	-49	-35	**40**
Pb	4	21	10	**110**		11	7.1	**55**	-47	-30	**57**
Pb	5	-	1.9	**-**		-	1.5	-	-	-18	-
Zn	1	1490	965	**54**		724	400	**81**	-51	-58	**-12**
Zn	2	244	136	**79**		122	78	**56**	-50	-42	**19**
Zn	3	128	78	**64**		74	64	**16**	-42	-18	**133**
Zn	4	107	70	**53**		70	70	**0**	-35	-0.4	**8650**
Zn	5	-	41	**-**		-	49	-	-	19	-
**Means**											
Cd				**60**				**46**			**-135**
Pb				**56**				**74**			**21**
Zn				**62**				**38**			**2198**
Total				**72**				**53**			**694**
