# Supplementary material for: Trends in spatial patterns of heavy metal deposition on national park service lands along the Red Dog Mine haul road, Alaska, 2001–2006
Source: PLoS One. 2017 May 18;12(5):e0177936. doi: 10.1371/journal.pone.0177936 (PMC5436859; doi:10.1371/journal.pone.0177936)
Supplement: S2 Table — Strata 5 exists only in the south, and it has no N:S ratio but is still included in the overall mean. Strata for which the 95% credible interval in average change spanned zero for the north or south side have higher uncertainty are shown in blue with a smaller font. (PDF) [file pone.0177936.s027.pdf]

| Stratum | N:S 2001 | N:S 2006 |
| --- | --- | --- |
| 1 | 1.48 | 1.77 |
| 2 | 1.85 | 1.63 |
| 3 | 1.71 | 1.22 |
| 4 | 1.60 | 1.05 |
| **All (1-4)** | **1.49** | **1.54** |
